# Single-cell proteo-genomic reference maps of the hematopoietic system enable the purification and massive profiling of precisely defined cell states

**DOI:** 10.1038/s41590-021-01059-0

**Published:** 2021-11-22

**Authors:** Sergio Triana, Dominik Vonficht, Lea Jopp-Saile, Simon Raffel, Raphael Lutz, Daniel Leonce, Magdalena Antes, Pablo Hernández-Malmierca, Diana Ordoñez-Rueda, Beáta Ramasz, Tobias Boch, Johann-Christoph Jann, Daniel Nowak, Wolf-Karsten Hofmann, Carsten Müller-Tidow, Daniel Hübschmann, Theodore Alexandrov, Vladimir Benes, Andreas Trumpp, Malte Paulsen, Lars Velten, Simon Haas

**Affiliations:** 1 Structural and Computational Biology Unit, European Molecular Biology Laboratory, Heidelberg, Germany; 2 Collaboration for Joint PhD degree between European Molecular Biology Laboratory and Heidelberg University, Faculty of Biosciences, Heidelberg, Germany; 3 Centre for Genomic Regulation (CRG), The Barcelona Institute of Science and Technology, Barcelona, Spain; 4 Heidelberg Institute for Stem Cell Technology and Experimental Medicine (HI-STEM gGmbH), Heidelberg, Germany; 5 Division of Stem Cells and Cancer, Deutsches Krebsforschungszentrum (DKFZ) and DKFZ–ZMBH Alliance, Heidelberg, Germany; 6 Faculty of Biosciences, Heidelberg University, Heidelberg, Germany; 7 Berlin Institute of Health (BIH), Charité Universitätsmedizin Berlin, Berlin, Germany; 8 Max Delbrück Center for Molecular Medicine in the Helmholtz Association, Berlin Institute for Medical Systems Biology, Berlin, Germany; 9 Department of Internal Medicine V, Heidelberg University Hospital, Heidelberg, Germany; 10 Genome Biology Unit, European Molecular Biology Laboratory, Heidelberg, Germany; 11 Flow Cytometry Core Facility, European Molecular Biology Laboratory, Heidelberg, Germany; 12 Department of Hematology and Oncology, Medical Faculty Mannheim, University of Heidelberg, Mannheim, Germany; 13 Computational Oncology, Molecular Diagnostics Program, National Center for Tumor diseases (NCT) Heidelberg and German Cancer Research Center (DKFZ), Heidelberg, Germany; 14 German Cancer Consortium (DKTK), Heidelberg, Germany; 15 Department of Pediatric Immunology, Hematology and Oncology, University Hospital Heidelberg, Heidelberg, Germany; 16 Skaggs School of Pharmacy and Pharmaceutical Sciences, University of California, San Diego, La Jolla, CA USA; 17 Genomics Core Facility, European Molecular Biology Laboratory, Heidelberg, Germany; 18 Novo Nordisk Foundation Center for Stem Cell Biology, DanStem. Faculty of Health and Medical Sciences Blegdamsvej, Copenhagen, Denmark; 19 Universitat Pompeu Fabra (UPF), Barcelona, Spain; 20 Charité-Universitätsmedizin, Berlin, Germany

**Keywords:** Gene expression analysis, Haematopoietic stem cells, Leukaemia, Haematopoiesis

## Abstract

Single-cell genomics technology has transformed our understanding of complex cellular systems. However, excessive cost and a lack of strategies for the purification of newly identified cell types impede their functional characterization and large-scale profiling. Here, we have generated high-content single-cell proteo-genomic reference maps of human blood and bone marrow that quantitatively link the expression of up to 197 surface markers to cellular identities and biological processes across all main hematopoietic cell types in healthy aging and leukemia. These reference maps enable the automatic design of cost-effective high-throughput cytometry schemes that outperform state-of-the-art approaches, accurately reflect complex topologies of cellular systems and permit the purification of precisely defined cell states. The systematic integration of cytometry and proteo-genomic data enables the functional capacities of precisely mapped cell states to be measured at the single-cell level. Our study serves as an accessible resource and paves the way for a data-driven era in cytometry.

## Abstract (web summary)

Haas, Velten and colleagues use single-cell multiomics of human blood and bone marrow to generate a reference map allowing the quantitative linking of cytometry and proteo-genomic information.

## Main

Single-cell transcriptomic technologies have revolutionized our understanding of tissues^1–3^. The systematic construction of whole-organ and whole-organism single-cell atlases has revealed an unanticipated diversity of cell types and cell states, and has provided detailed insights into cellular development and differentiation processes^4–7^. However, strategies for the prospective isolation of cell populations newly identified by single-cell genomics are needed to enable their functional characterization or therapeutic use. Furthermore, single-cell genomics technologies remain cost-intense and scale poorly, impeding their integration into clinical routine.

Unlike single-cell transcriptomics, flow cytometry offers a massive throughput in terms of samples and cells, is commonly used in routine clinical diagnostics^8^ and remains unrivaled in the ability to prospectively isolate live populations of interest for downstream applications. However, flow cytometry provides low-dimensional measurements and relies on predefined sets of surface markers and gating strategies that have evolved historically in a process of trial and error. Hence, single-cell transcriptomics (scRNA-seq) approaches have demonstrated that flow cytometry gating schemes frequently yield impure or heterogeneous populations^9,10^, and flow strategies for the precise identification of cell types defined by scRNA-seq are lacking. Conversely, the precision and efficiency of commonly used cytometry gating schemes are largely unknown, and the exact importance of many surface markers remains unclear. Together, these findings highlight a disconnect between single-cell genomics-based molecular cell type maps and data generated by widely used cytometry assays.

The differentiation of hematopoietic stem cells (HSCs) in the bone marrow (BM) constitutes a particularly striking example of this disconnect^11–14^. The classical model of hematopoiesis, which is based mainly on populations defined by flow cytometry^15–17^, has recently been challenged in several aspects by single-cell transcriptomic^9,10,18–20^, functional^21,22^ and lineage tracing^23^ approaches. These studies revealed that hematopoietic lineage commitment occurs earlier than previously anticipated, that putative oligopotent progenitors isolated by fluorescence activated cell sorting (FACS) consist of heterogeneous mixtures of progenitor populations and that lineage commitment is represented most accurately by a continuous process of differentiation trajectories rather than by a stepwise differentiation series of discrete progenitor populations^12–14,24^. The frequency of functionally oligopotent progenitors in immunophenotypic hematopoietic stem and progenitor cell (HSPC) gates remains controversial^9,25,26^. These discrepancies have contributed to conflicting results between studies that employ scRNA-seq for the definition of progenitor populations^9,10,18,19,27^ and studies that use FACS^15,16,28^. As a consequence, flow-based assays that accurately reflect the molecular and cellular complexity of the hematopoietic system are urgently needed.

Recently, methods to simultaneously measure mRNA and surface protein expression in single cells have been developed^29,30^. Here, we demonstrate that ultrahigh content single-cell proteo-genomic reference maps, alongside appropriate computational tools, can be used to systematically design and analyze cytometry assays that accurately reflect scRNA-seq-based molecular tissue maps at the level of cell types and differentiation states. For this purpose, we have generated proteo-genomic datasets encompassing 97–197 surface markers across 122,004 cells representing the cellular landscape of young, aged and leukemic human BM and blood, as well as all states of HSC differentiation. We demonstrate how such data can be used in an unbiased manner to evaluate and automatically design cytometry gating schemes for individual populations and entire biological systems without previous knowledge. We show that, compared with existing approaches, such optimized schemes are superior in the identification of cell types and more accurately reflect molecular cell states. Projecting datasets from malignant hematopoiesis on our reference atlases enables the fine-mapping of the exact stage of differentiation arrest in leukemias, the identification of leukemia-specific surface markers and an unsupervised classification of disease states. Finally, we demonstrate how such data resources can be used to project low-dimensional cytometry data on single-cell genomic atlases to enable functional analysis of precisely defined states of cellular differentiation. Our data resource and bioinformatic advances enable the efficient identification and isolation of any molecularly defined cell state from blood and BM while laying the grounds for reconciling flow cytometry and single-cell genomics data across human tissues.

## Results

### A single-cell proteo-genomic reference map of BM

**Fig. 1:**
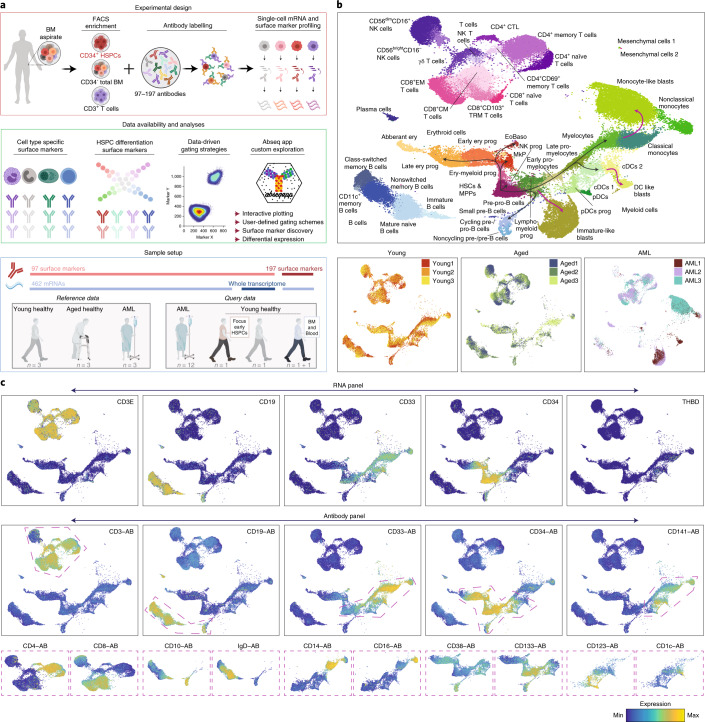
A comprehensive single-cell proteo-genomic map of young, aged and malignant BM. **a**, Overview of the study. See Methods and main text for details. **b**, Top: UMAP display of single-cell proteo-genomics data of human BM from healthy young, healthy aged and AML patients (*n* = 70,017 single cells, 97 surface markers), integrated across *n* = 9 samples and data modalities. Clusters are color-coded. ery, erythroid; prog, progenitor. Bottom: UMAPs highlighting sample identities. See Supplementary Note 5 for details of cluster annotation. The whole transcriptome Abseq data is presented in Supplementary Note 2, the Abseq experiments with measurements of 197 surface markers are presented in Extended Data Fig. 4. **c**, Normalized expression of selected mRNAs and surface proteins highlighted on the UMAP space from **b**. Top: expression of mRNAs encoding surface markers widely used to identify main cell types. Middle: expression of the corresponding surface proteins. Bottom: expression of markers widely used to stratify main cell types into subtypes. Only the parts of the UMAPs highlighted by dashed polygons in the middle row are shown. For all data shown throughout the manuscript, BM mononuclear cells from iliac crest aspirations from healthy adult donors or AML patients were used unless stated otherwise.

**Extended Data Fig. 1:**
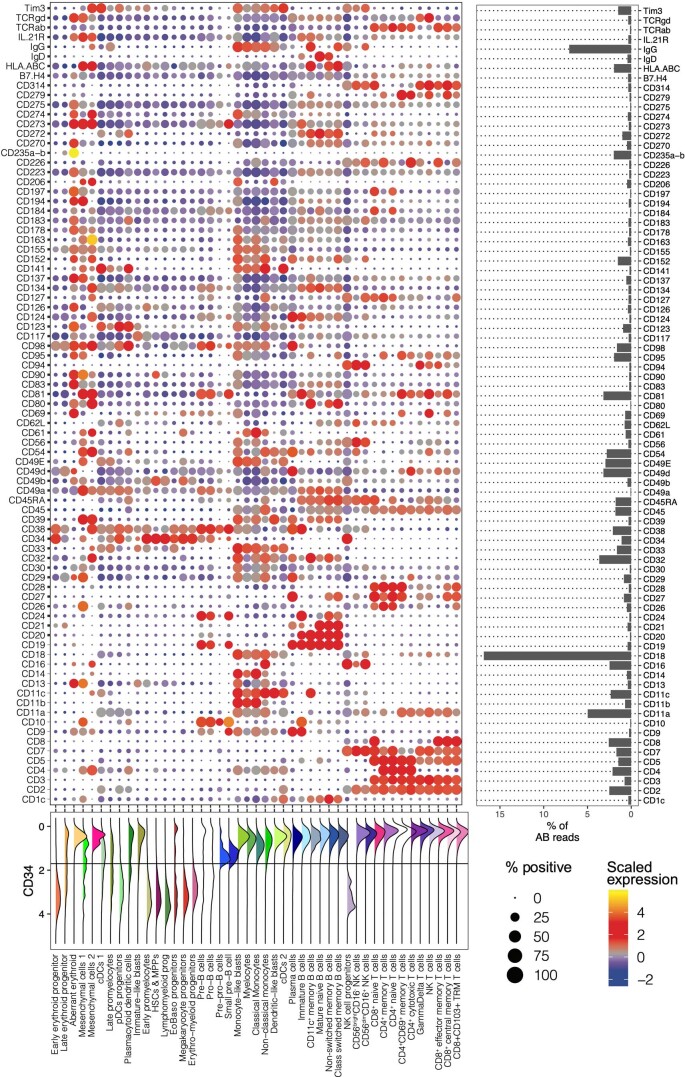
A proteo-genomic single-cell map of 97 surface markers in human bone marrow. *Related to* Fig. 1. Dot plot depicting the expression of all surface markers by cell type. Color indicates mean normalized expression, point size indicates the fraction of cells positive for the marker. Automatic thresholding was used to identify positive cells, see Methods, section *‘Thresholding of surface marker expression’* for details. The panel on the right depicts the fraction of total reads obtained for each marker as a proxy for absolute expression levels. Bottom panel illustrates the distribution of CD34 + expression across populations, similar plots can be generated for any marker using the Abseq App.

**Extended Data Fig. 2:**
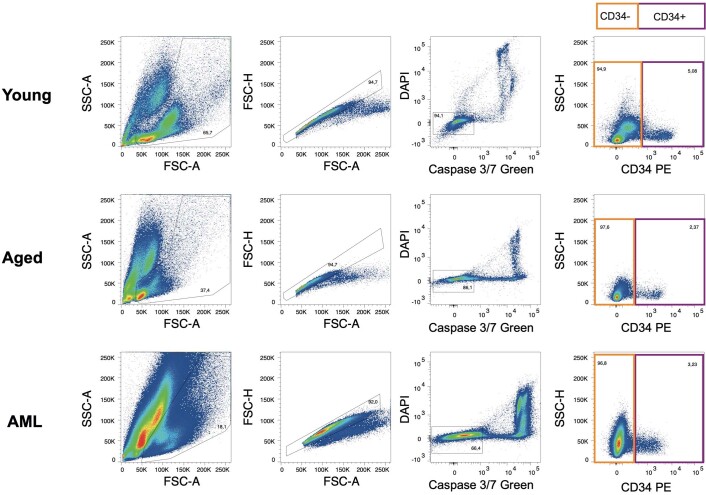
Representative gating schemes used for the enrichment of CD34+ cells. Related to Fig. 1. For additional information on cell sorting setups, see Methods, section ‘Cell sorting for Abseq’.

To establish a comprehensive single-cell transcriptomic and surface protein expression map of human BM, we performed a series of Abseq experiments in which mononuclear BM cells from hip aspirates were labeled with 97–197 oligo-tagged antibodies, followed by targeted or whole transcriptome scRNA-seq on the BD Rhapsody platform (Fig. 1a). For targeted single-cell transcriptome profiling, we established a custom panel, consisting of 462 mRNAs covering all HSPC differentiation stages, cell type identity genes, mRNAs of surface receptors and additional genes that permit the characterization of cellular states. These genes were selected systematically to capture all relevant layers of RNA expression heterogeneity observed in this system (Supplementary Note 1 and Supplementary Table 1). Whole transcriptome single-cell proteo-genomics confirmed that no populations were missed due to the targeted nature of the assay (Supplementary Note 2). Using this panel, in combination with 97 surface markers (Supplementary Table 2), we analyzed the BM of three young healthy donors, three aged healthy donors and three acute myeloid leukemia (AML) patients at diagnosis (Fig. 1a, Extended Data Fig. 1 and Supplementary Table 3). For samples from healthy donors, CD34^+^ cells were enriched to enable a detailed study of HSC differentiation (Extended Data Fig. 2). For samples from AML patients, CD3^+^ cells were enriched in some cases to ensure sufficient coverage of T cells.

**Extended Data Fig. 3:**
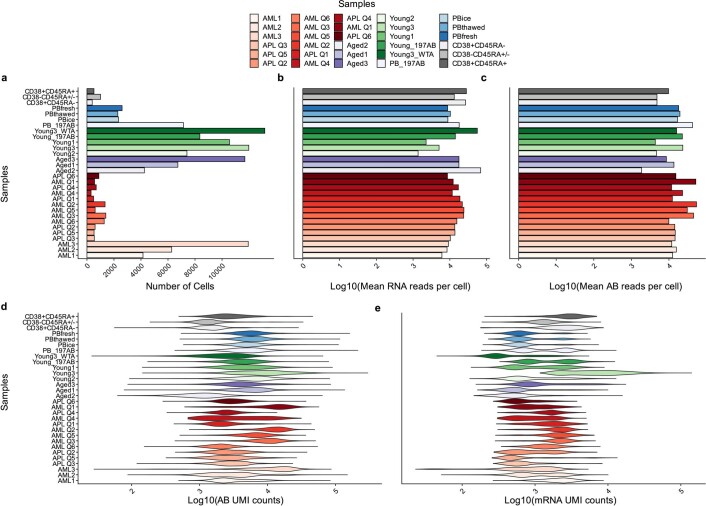
Sequencing statistics. Related to Fig. 1. Plots depict **a**. The number of cells passing filters. Note that samples AML Q1-Q6 and APQ1–6 were multiplexed (hashed) into one experiment. **b**, **c**. The sequencing depth on the surface and mRNA level and **d**, **e**. The number of surface and mRNA molecules per cell observed. Note that targeted mRNA sequencing was performed as described in the main text.

Since single-cell proteo-genomic approaches are not commonly performed at this level of antibody multiplexing, we designed a series of control experiments. First, we performed matched Abseq experiments in the presence or absence of antibodies to ensure that highly multiplex antibody stains do not effect the transcriptome of single cells (Supplementary Note 3). We further performed a series of Abseq experiments on fresh and frozen samples to demonstrate that the freeze–thawing process has no great impact on the data (Supplementary Note 3). Finally, we evaluated the sequencing requirements for optimal cell type classification in high-parametric single-cell proteo-genomic experiments (Supplementary Note 4). In the main reference data set, 70,017 high-quality BM cells were profiled with combined RNA and high-parametric surface protein information, and an average of ~7,500 surface molecules per cell were detected (Extended Data Fig. 3). Following data integration across experiments and measurement modalities, we identified 45 cell types and cell stages covering the vast majority of previously described hematopoietic cell types of the BM and peripheral blood (PB), including all stages of HSC differentiation in the CD34^+^ compartment, all T cell and natural killer (NK) cell populations of the CD3^+^ and CD56^+^ compartments, several dendritic cell and monocyte subpopulations from the CD33^+^ compartment and all main B cell differentiation states across CD10^+^, CD19^+^ and CD38^high^ compartments (Fig. 1b,c, Supplementary Note 5 and Supplementary Table 4). In addition, poorly characterized populations, such as cytotoxic CD4^+^ T cells and mesenchymal stem or stromal cells (MSCs) are covered. Cells from young and aged BM occupied the same cell states in all individuals, whereas cell states in AML differed (Fig. 1b and see below). Importantly, the combined RNA and surface protein information provided higher resolution and revealed cell types that are not readily identified by one of the individual data layers alone (Supplementary Note 6).

**Extended Data Fig. 4:**
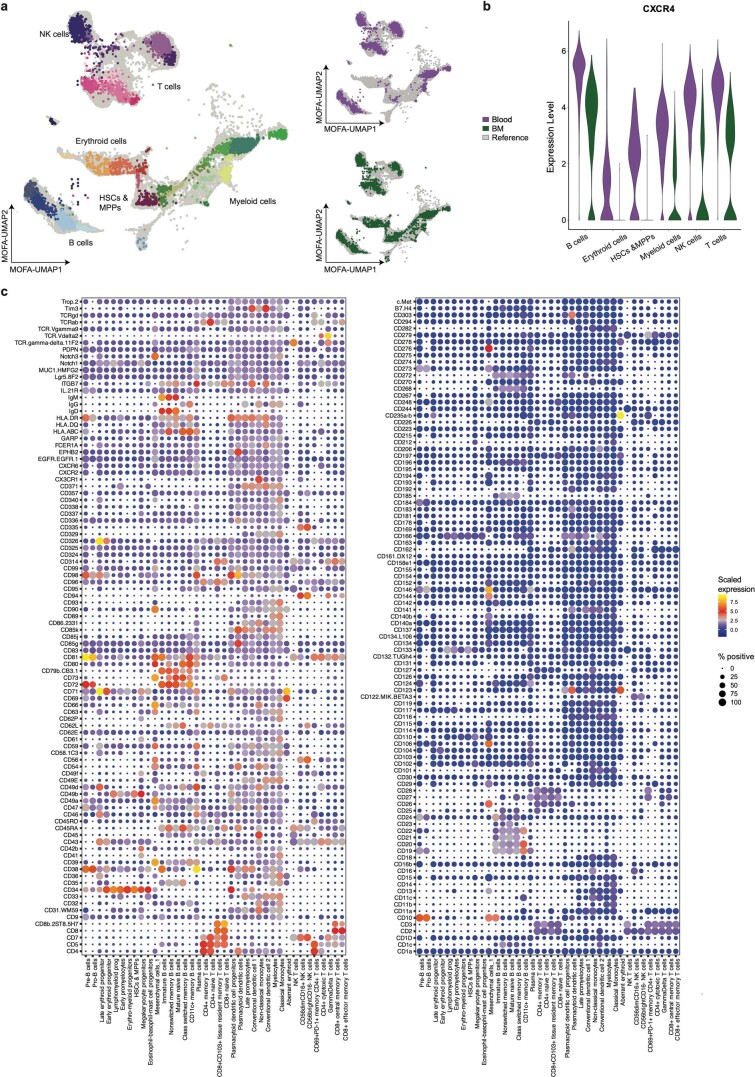
A single-cell proteo-genomic map of 197 surface markers in human bone marrow and blood. *Related to* Fig. 1. **a**. Left: UMAP projection on the original coordinate system from the healthy dataset (see Supplementary Note 7). Cells are colored by the mapped cell type. Right: UMAP colored by sample origin (blood and bone marrow). **b**. Violin plot depicting the expression of the bone marrow homing receptor CXCR4 on matching cell types of the blood and bone marrow. **c**. Dot plot depicting the expression of all surface markers by cell type. Color indicates mean normalized expression, point size indicates the fraction of cells positive for the marker. Automatic thresholding was used to identify positive cells, see Methods, *section ‘Thresholding of surface marker expression’* for detail.

Besides our main reference dataset, we generated ‘query‘ single-cell proteo-genomic datasets, which are displayed in the context of the main reference (Supplementary Note 7). These include, first, the analyses of healthy BM and matched PB samples using a 197-plex antibody panel to query the expression of additional surface markers in the context of our reference (Extended Data Fig. 4 and Supplementary Table 2). Second, the analyses of healthy BM analyzed with a 97-plex antibody panel in combination with whole transcriptome profiling to query any gene’s expression in the space defined by our reference (Supplementary Note 2). Third, the profiling of the CD34^+^CD38^−^ BM compartment with a 97-plex antibody panel to provide higher resolution of immature HSPCs (see below and Extended Data Fig. 9c,d) and fourth, a cohort of 12 AML patients (see below and Fig. 4). To make our comprehensive resource accessible, we developed the Abseq App, a web-based application that permits visualization of gene and surface marker expression, differential expression testing and the data-driven identification of gating schemes across all datasets presented in this manuscript. A demonstration video of the app is available in the supplement (Supplementary Video 1). The Abseq App is accessible at: https://abseqapp.shiny.embl.de/.

### A directory of the biological importance of surface markers

**Fig. 2:**
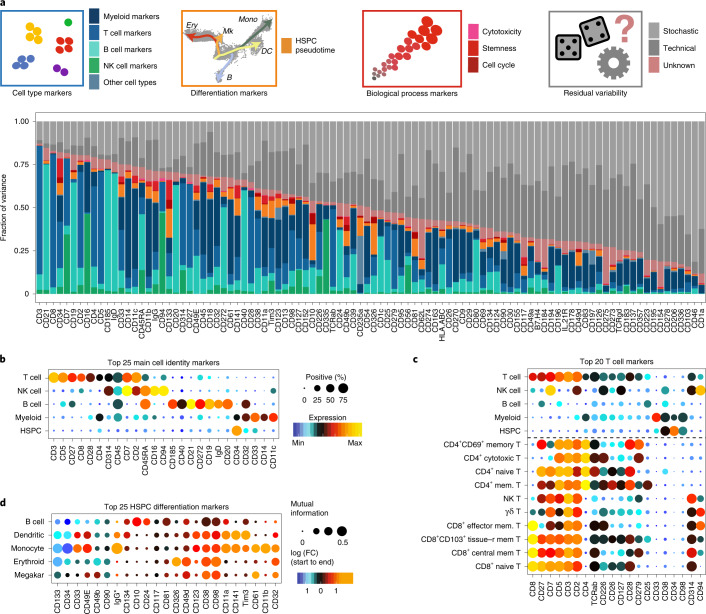
Association of surface marker expression with cell type identities, cellular differentiation and biological processes. **a**, For each surface marker measured in our 97-plex Abseq data, the fraction of variance explained by different covariates (colored insets in top row) is displayed. For this, every single cell from healthy young individuals (*n* = 3 samples, 28,031 single cells) was assigned to a cell type identity (blue inset, see Fig. 1b), and cytotoxicity, stemness and cell cycle scores (red inset, see Extended Data Fig. 5e) as well as technical covariate scores were determined. Additionally, pseudotime analyses were used to assign differentiation scores to HSPCs (orange inset, see Fig. 3a). These covariates were then used to model surface marker expression in a linear model. The fraction of variance explained by each of the processes was quantified. See Methods, section Modeling variance in surface marker expression for details. **b**, Cell type identity markers. Dot plot depicting the expression of the 25 surface markers with the highest fraction of variance explained by cell type across main populations. Colors indicate mean normalized expression, point size indicates the fraction of cells positive for the marker. Automatic thresholding was used to identify positive cells, see Methods, section Thresholding of surface marker expression for details. **c**, T cell subtype markers. The expression of the 20 surface markers with the highest fraction of variance explained by T cell subtype is displayed, legend as in **b**. mem, memory; tissue-r, tissue-resident. **d**, HSPC differentiation markers. Megakar, megakaryocytic. Dot plot depicting expression changes of markers across pseudotime in CD34^+^ HSPCs. Color indicates logarithmic fold change (FC) between the start and the end of each pseudotime trajectory. Point size indicates the mutual information in natural units of information between pseudotime and marker expression. The 25 surface markers with the highest fraction of variance explained by pseudotime covariates are displayed.

**Extended Data Fig. 5:**
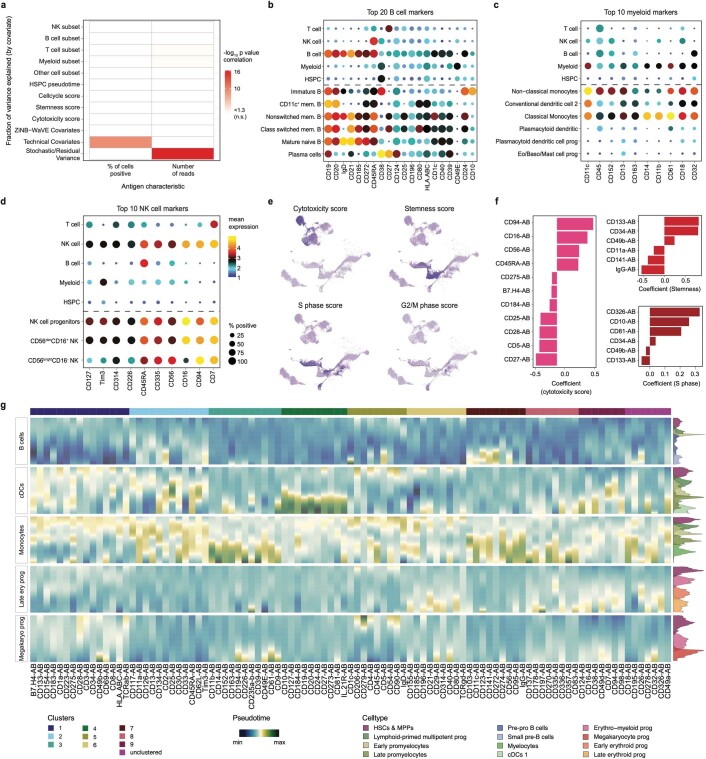
Markers of cell types and biological processes. *Related to* Fig. 2. **a**. Heatmap investigating if the fraction of variance explained by the different covariates is correlated to antigen-level technical covariates. P values were calculated from Pearson correlation using a one-sided test based on the t-distribution. **b**-**d**. Dot plot depicting the expression of the 10–20 surface markers with the highest fraction of variance explained by B cell subtype *(b)*, myeloid subtype *(c)* and NK cell subtype *(d)*. Color indicates mean normalized expression, point size indicates the fraction of cells positive for the marker. Automatic thresholding was used to identify positive cells, see Methods, section *‘Thresholding of surface marker expression’* for details. **e**. UMAPs highlighting the scores for various biological processes, as computed using the gene lists from Supplementary Table 7. **f**. Bar charts depicting the markers with the highest fraction of variance explained by cytotoxicity score (pink), stemness score (red) and S-phase score (dark red), and the corresponding model coefficients. See Supplementary Table 7 for the gene lists used for calculating these scores. **g**. Pseudotime of all 97 surface proteins for the five trajectories (B cells, cDCs, Monocytes, Late erythroid progenitor and Megakaryocyte progenitor). Markers were clustered according to their expression pattern using tradeseq (van den Berge, 2020). The density plots indicate the differentiation stages along the pseudotime.

While surface markers are widely used in immunology, stem-cell biology and cancer research to identify cell types, cell stages and biological processes, the exact importance of individual markers frequently remains ambiguous. To link surface marker expression quantitatively with biological processes, we assigned each cell in our data set to its respective cell type, and determined its differentiation stage, its stemness score, its cytotoxicity score and its current cell cycle phase as well as technical covariates (see Methods and below). Moreover, we included covariates representing unknown biological processes that were defined in an unsupervised manner using a factor model. Nontechnical covariates were not affected by marker expression level (Extended Data Fig. 5a and Methods). For each surface marker, we then quantified the fraction of variance of expression that is determined by any of these processes (Fig. 2a). This model identified markers that represent cell type identities or differentiation stages, as well as stemness, cytotoxicity and cell cycle properties (Fig. 2b–d and Extended Data Fig. 5b–f).

**Fig. 3:**
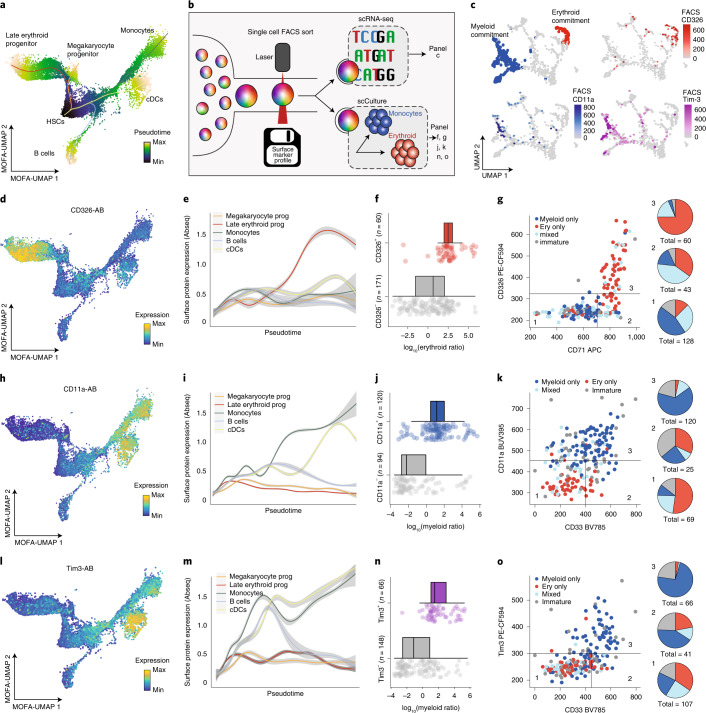
Validation of novel stage-specific HSPC differentiation markers. **a**, UMAP plot depicting CD34^+^ HSPCs and their pseudotime scores along five differentiation trajectories, see Methods, section Pseudotime analysis. The normalized pseudotime score across all lineages is color-coded. **b**, Scheme illustrating the experiments performed to validate the importance of selected markers. See main text and Supplementary Note 8 for details. **c**, UMAP display of mRNA expression of *n* = 630 CD34^+^ cells from a single-cell Smart-seq2 experiment where surface markers were recorded using FACS. For a detailed description of the experiment, see Supplementary Note 8. Upper left panel: cells with myeloid and erythroid gene expression signatures are highlighted on the UMAP. Remaining panels: surface protein expression (FACS data) of indicated markers is shown. **d**, UMAP display highlighting the normalized CD326 surface protein expression (Abseq data). **e**, Line plots depicting normalized CD326 surface protein expression (Abseq data) smoothed over the different pseudotime trajectories illustrated in **a**. Error ribbon indicates 95% confidence interval from the smoothing GAM model. **f**, Boxplots depicting the ratio in erythroid cells produced in single-cell cultures in relation to the CD326 expression of the founder cell (*n* = 231 single-cell derived colonies). See Methods, section Data visualization for a definition of boxplot elements. **g**, Left panel: scatter plots depicting the differentiation potential of single founder cells in relation to their CD326 and CD71 surface expression. The founder cell potential was categorized by its ability to give rise to (red) erythroid only progeny, (skyblue) a mix of erythroid, myeloid or any other progeny, (blue) only myeloid progeny or (gray) remaining, immature cells. Right panel: founder cells were subset according to their CD326 and CD71 surface expression status and relative fractions of their respective potential are summarized as pie charts. **h**–**o**, Analysis of CD11a and Tim3. **h**–**k** as in **d**–**g** except that CD11a is shown in the UMAP (**h**), line plot (**i**), boxplot (**j**) and scatter plot (**k**). **l**–**o**, Panels are analogous to **d**–**g**, except that Tim3 expression is shown in the UMAP (**l**) line plot (**m**), boxplot (**n**) and scatter plot (**o**). For scatter plots in **k** and **o**, CD11a or Tim3 expression was plotted against the myeloid differentiation marker CD33. For **j**,**k**,**n**,**o**, *n* = 214 single-cell derived colonies. Source data

**Extended Data Fig. 6:**
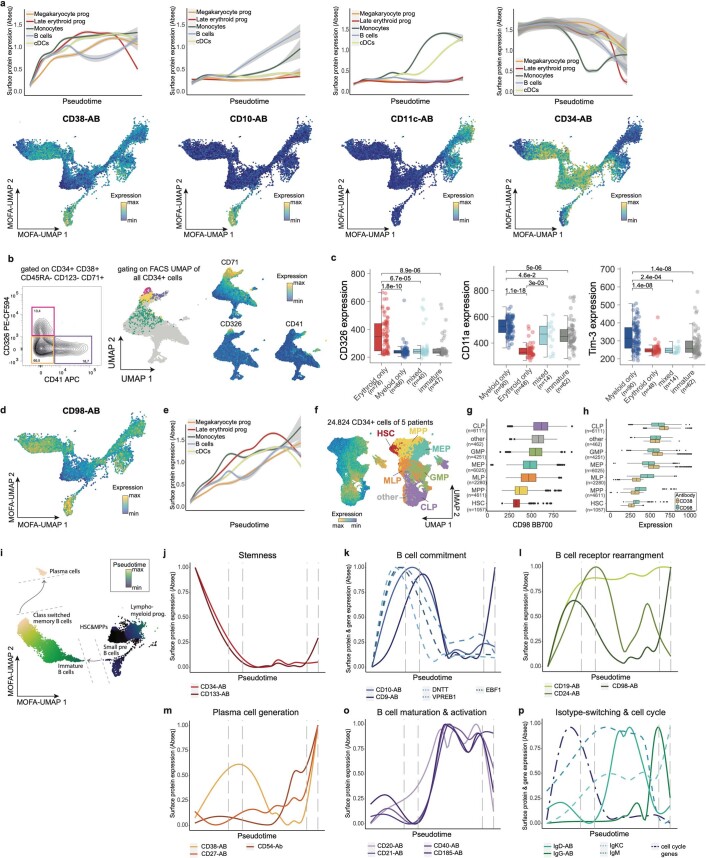
Surface markers associated with HSC and B cell differentiation. Related to Figs. 2 and 3. See methods, section *Data visualization* for a definition of boxplot elements. **a**. Top: Line of surface protein expression smoothened over pseudotime (see Fig. 3a). Error ribbon indicates 95% confidence interval from the smoothing GAM model. Bottom: UMAP display of marker expression in CD34 + HSPCs. **b**. Left: Gating strategy for subsetting CD71 + erythroid/megakaryocytic HSPCs into CD41 + megakaryocyte and CD326 + erythroid progenitors. Right: UMAP display of flow cytometric data from CD34 + cells from a healthy donor analyzed with a 12-color FACS panel for erythroid/megakaryocytic differentiation (Supplementary Table 6). Feature plots of CD71, CD326 and CD41 expression highlight the bifurcation within CD71 + HSPCs. **c**. Culture outcome categories described in Fig. 3g were analyzed with regards to their CD326, CD11a or Tim3 surface expression. A two-sided Wilcoxon rank sum test was used for comparison of individual groups and significance levels between groups. P-values were adjusted for multiple comparisons using the Holm method. **d**, **e**. Like Fig. 3d, e, except that CD98 expression is shown. **f**. UMAP display of flow cytometric data from CD34 + cells from five healthy donors analyzed with a 12-color FACS stem and progenitor panel (Supplementary Table 6). Left: shows CD98 surface expression, right panel shows assignment of individual gates to the UMAP according, as follows: HSC: CD34 + CD38-CD45RA-CD90 + ; MPP: CD34 + CD38-CD45RA-CD90-; MLP: CD34 + CD38-CD45RA + ; MEP: CD34 + CD38 + CD10-CD45RA-; GMP: CD34 + CD38 + CD10-CD45RA + ; CLP: CD34 + CD38 + CD10 + CD45RA + . **g**. Boxplots showing CD98 expression in individual cell populations mentioned in *f*. **h**. Boxplots showing co-expression of CD98 and CD38 markers. **i**. Like Fig. 3a, UMAP depicting the pseudotime score along the B cell differentiation trajectory emanating from CD34 + HSCs & MPPs and Lymphomyeloid progenitors. **j**–**p**. Line plots depicting surface expression representative for different biological processes smoothened over the B cell pseudotime trajectory. Source data

To characterize new markers identified by this analysis, we focused initially on the evaluation of surface molecules that specifically mark distinct stages of HSC differentiation, since a lack of specific markers currently impedes the accurate representation of lineage commitment by flow cytometry^9,10,18,21,27^. For this purpose, we performed pseudotime analyses within the CD34^+^ HSPC compartment and identified surface markers that correlate with the progression of HSCs towards erythroid, megakaryocytic, monocyte, conventional dendritic cell or B cell differentiation trajectories (Methods; Figs. 2d and 3a and Extended Data Fig. 5g). Of note, the monocyte trajectory also includes neutrophil progenitor stages, but mature neutrophils are not included in the datasets due to the use of density gradient centrifugation of samples. Moreover, trajectory analyses were not performed for plasmacytoid dendritic and eosinophil/basophil lineages due to a low number of intermediate cells impeding the unanimous identification of branch points. Pseudotime analyses quantified the exact expression dynamics of many well-established markers, such as CD38 as a pandifferentiation marker, as well as CD10 and CD11c as early B cell and monocyte-dendritic cell lineage commitment markers, respectively (Fig. 2d and Extended Data Fig. 6a). Importantly, our analyses revealed new surface markers that specifically demarcate distinct stages of lineage commitment, including CD326, CD11a and Tim3 (Figs. 2d and 3). To confirm the high specificity of these markers for erythroid and myeloid commitment, respectively, we used FACS-based indexing of surface markers coupled to single-cell RNA-seq (‘index scRNA-seq’, see also Supplementary Note 8), or coupled to single-cell cultures (‘index cultures’) (Fig. 3b). As suggested by our proteo-genomic single-cell data, CD326 expression was associated with molecular priming and functional commitment into the erythroid lineage (Fig. 3c–g and Extended Data Fig. 6b,c). By contrast, Tim3 and CD11a were identified as panmyeloid differentiation markers and were associated with transcriptomic priming and functional commitment into the myeloid lineage (Fig. 3c,h–o and Extended Data Fig. 6c). Finally, CD98 was identified as a new pandifferentiation marker of HSCs, which we confirmed by classical flow cytometry (Fig. 2d and Extended Data Fig. 6d–h). Beyond the progression of HSCs to lineage-committed cells, we also analyzed the surface marker dynamics throughout B cell differentiation, allowing us to identify markers specific to their lineage commitment, maturation, isotype switching and final plasma cell generation (Extended Data Fig. 6i–p).

Our model provides a global and quantitative understanding of how well cell type identities, differentiation stages and biological processes are related to the expression of individual surface markers. A comprehensive overview of surface markers associated with these processes is depicted in the supplement (Supplementary Data 1 and Extended Data Fig. 5).

### Surface protein expression in healthy aging and cancer

**Fig. 4:**
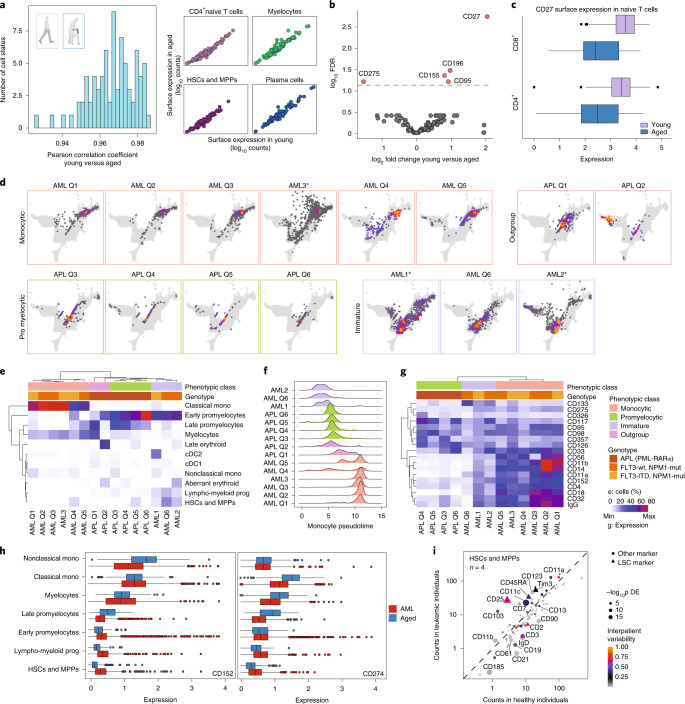
Adaptation of surface protein expression in healthy aging and cancer. **a**, Correlation of surface marker expression between matched cell types from aged and young BM donors. For each cell type, mean surface marker expression across all cells was computed, separately for all ‘young’ and ‘aged’ samples. Left panel: histogram of Pearson correlation coefficients. Right panel: sample scatter plots depicting the mean surface expression of all measured markers in indicated cell types. **b**, Volcano plot depicting log_2_ fold change and false discovery rate (FDR) for a test for differential surface marker expression between cells from young and aged individuals, while accounting for cell types as covariates. See Methods for details. **c**, Boxplots depicting CD27 surface expression in naïve T cell populations from young and aged individuals. Sample size is provided as Figure Source Data. See Methods, section Data visualization for a definition of boxplot elements. **d**, Projection of AML samples onto healthy reference. See Supplementary Note 7 for details. **e**, Clustering of leukemia samples by their projected cell type composition. Lymphoid cells are excluded from the clustering. **f**, Density plots of monocyte pseudotime, resulting from projection on the healthy reference. See Methods for details. **g**, Heatmap depicting surface markers with differential expression between the phenotypic classes defined in **e**. The eight markers with the most significant *P* values from DESeq2 were selected for each comparison between classes. Average expression across all nonlymphoid cells is shown. ITD, internal tandem duplication; mut, mutation; wt, wild type. **h**, Surface expression of immunotherapy targets CTLA4 (CD152) and PD-L1 (CD274) in different myeloid compartments of healthy donors and AMLs. Sample size is provided as Figure Source Data. **i**, Scatter plot depicting the average expression of all surface markers in healthy HSCs and MPPs (*x* axis) and leukemic stem cells (LSC) projecting to the HSC and MPP cell state (*y* axis). Cells from four patients where the HSC/MPP class was covered with more than 20 cells are included (AML1, AML2, AML3 and AML Q6). *P* values for differential expression were computed using DESeq2 and are encoded in the symbol size, and previously described LSC markers are depicted as a triangle. Interpatient variability is color-coded, see Methods, for details. See also Supplementary Data 2. Source data

**Extended Data Fig. 7:**
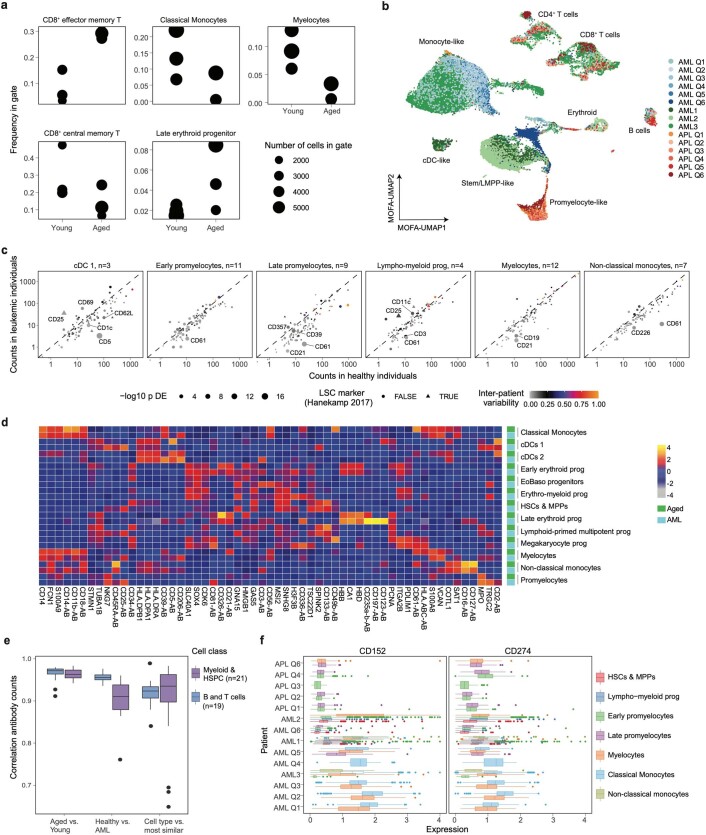
Changes in surface protein expression and cell type abundance induced by ageing and leukemia. Related to Fig. 4. **a**. Frequency of selected cell types in young and aged individuals. Only cell types with the highest significant changes are shown, see Methods, section ‘*Changes in cell type abundance between experimental groups*’. **b**. UMAP display of all AML patients. Data were integrated using scanorama and MOFA (see Method *‘Data analysis of Abseq data’* and *‘MOFA integration, Clustering, and identification of cell type markers’*). **c**. For every myeloid cell state with sufficient representation of ≥ 20 cells in at least three patients, surface marker expression between AML (x-axis) and healthy individuals (y-axis) is compared. AML cell types were defined using a projection as in Fig. 4d, e. P-values for differential expression were computed using DESeq2 and encoded in the symbol size. Inter-patient variability is color-coded (n = number of patients included), see Methods, section ‘*Differential expression testing between experimental groups and estimation of inter-patient variability’* and Supplementary Data 2. **d**. Heatmap depicting cell state specific gene expression in leukemic and healthy individuals. Five most significantly overexpressed markers were identified for each cell state, using only leukemic cells. The expression of all markers selected is shown and compared to their expression in the corresponding healthy cell states. **e**. Correlations of surface marker expression are shown for matching cell types from young versus aged individuals, from healthy individuals versus AML patients, and for cell types versus the transcriptomically most similar cell type available in the dataset. See Methods, section *Data visualization for a definition of boxplot elements*. **f**. Boxplot depicting the expression of CD152 and CD274 in different cell states from different patients. Only populations covered with ≥ 50 cells per patient are included (Fig. 4h) and see source data (*Source Data* Extended Data Fig. 7) for sample size. Source data

To investigate surface protein expression throughout healthy aging, we compared Abseq data of BM from young and aged healthy individuals. These analyses revealed that the expression of surface molecules was highly similar across all BM populations between age groups (Fig. 4a,b and Supplementary Data 1), suggesting unexpectedly stable and highly regulated patterns of surface protein expression that are affected only modestly by aging. While cell type frequencies were also affected only modestly by aging, a substantial accumulation of cytotoxic effector CD8^+^ T cells was observed^31^ (Extended Data Fig. 7a). Moreover, the expression of several immune regulatory molecules showed age-related changes in surface presentation, including the death receptor FAS (CD95), the poliovirus receptor (CD155) and the ICOS ligand (CD275) (Fig. 4b). In particular, naive CD8^+^ and CD4^+^ T cell subsets displayed an aging-associated decline in surface expression of CD27, a costimulatory molecule required for generation and maintenance of long-term T cell immunity^32^ (Fig. 4b,c). Together, these analyses suggest that the overall pattern of surface protein expression is widely maintained upon healthy aging, whereas specific changes, most prominently in the surface presentation of immune regulatory molecules, occur.

We next explored surface marker remodeling in AML—a blood cancer characterized by the accumulation of immature, dysfunctional myeloid progenitors, also called blasts. While the cellular BM of healthy donors displayed highly similar topologies across six individuals, initial analysis of three AML patients demonstrated that leukemic cells showed patient-specific alterations and a large degree of interpatient variability (Fig. 1b). To develop a generically applicable workflow to interpret data from hematological diseases in the context of our reference, we generated single-cell proteo-genomics datasets from a total of 15 AML patients, covering six t(15;17) translocated acute promyelocytic leukemias and nine normal karyotype AMLs with *NPM1* mutations, of which four patients carried an additional *FLT3* internal tandem duplication (Supplementary Table 3). While an unsupervised integration of these data highlighted primarily patient-to-patient variability (Extended Data Fig. 7b), projecting cells onto our healthy reference enabled a fine-mapping of the differentiation stages of leukemia cells (Fig. 4d and Supplementary Note 7). Unsupervised clustering of patients on the basis of relative abundancies of differentiation stages revealed three main categories: ‘monocytic AMLs’ that displayed an extensive accumulation of blasts with classical monocyte phenotype, acute promyelocytic leukemias that were blocked in early and late promyelocyte states, and ‘immature AMLs’ that showed high numbers of immature blasts resembling HSC, multipotent progenitors (MPP), early lymphomyeloid progenitor and early promyelocyte states (Fig. 4e,f). In general, leukemic blasts retained many features reminiscent of the cell stage they were blocked in (Extended Data Fig. 7c–e). Accordingly, differential expression analyses revealed that many surface markers that distinguish the different AML states also mark their corresponding healthy counterparts, such as CD133 for immature AMLs or CD14 and CD11b for monocytic AMLs (Fig. 4g). This also translated into differential surface expression of potential drug targets, such as PD-L1 (CD274) and CTLA4 (CD152) (Fig. 4h and Extended Data Fig. 7f), suggesting that the myeloid differentiation program of the AML might be essential in the treatment choice of targeted immune therapies.

By contrast, differential analyses between AML and healthy cells from the same differentiation stage revealed markers specifically overexpressed in leukemic cells (Fig. 4i, Extended Data Fig. 7c and Supplementary Data 2). Interestingly, these analyses readily identified several previously described leukemia stem-cell markers, including CD25, Tim3, CD123 and CD45RA^33^, supporting the validity of our approach. Quantifying the degree of interpatient heterogeneity of each marker while accounting for cell state revealed that many known leukemia stem-cell markers vary strongly in their expression between patients (Fig. 4i). Together, this workflow of projection to a well-annotated healthy reference in combination with cell-state-specific differential expression testing might become a standard in scRNA-seq analyses of hematological diseases. Our computational routines are available online at https://git.embl.de/triana/nrn.

### Data-driven flow cytometry for immunology

**Fig. 5:**
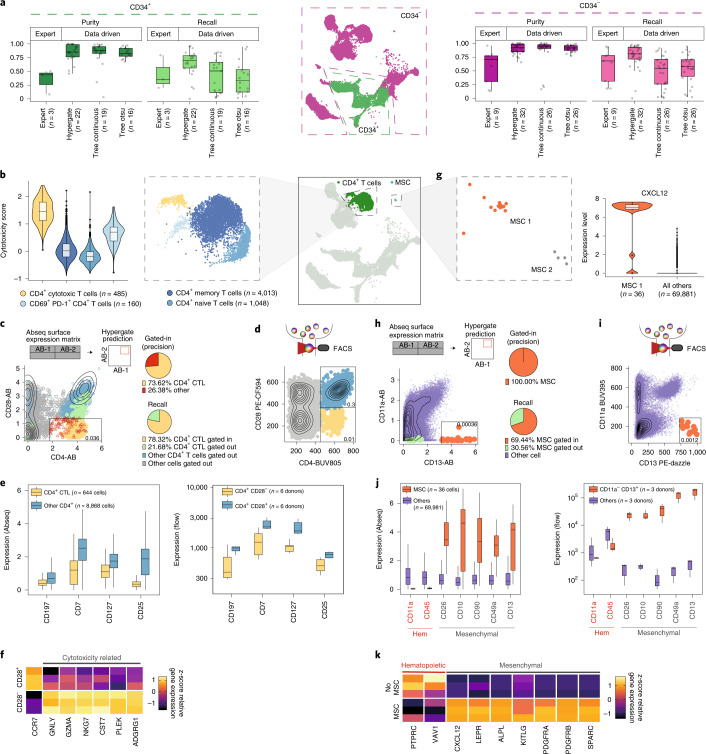
Data-driven definition of gating schemes for rare cell types. Boxplot sample sizes are provided in the figure. See Methods, section Data visualization for a definition of boxplot elements. **a**, Purity and recall of published or data-driven gating schemes for cell populations within CD34^+^ and CD34^−^ compartments, see also Extended Data Fig. 8. **b**, Different CD4^+^ T cell subsets are highlighted (central and right panels) and the corresponding distributions of cytotoxicity scores for every subset are displayed (left panel). **c**, Hypergate^34^ was used to identify a gating scheme for the isolation of cytotoxic CD4^+^ T cells. The suggested gate is highlighted on a scatter plot of CD4 and CD28 expression as identified from pregated CD45^+^ CD3^+^ Abseq data. Pie charts indicate precision and recall. **d**, FACS plot displaying the expression of CD4 and CD28 on pregated CD45^+^ CD3^+^ cells, and respective gates. **e**, Boxplot depicting the expression of surface markers with differential expression between CD4^+^ cytotoxic T cells and other CD4^+^ subsets, as identified from Abseq data (left panel) and validated with FACS using the gating strategy from **d** (right panel). **f**, Heatmap depicting gene expression of cytotoxicity-related genes in FACS-sorted CD4^+^ CD28^−^ and CD4^+^ CD28^+^ cells, as quantified by qPCR (*n* = 3 patients). **g**–**j**, Analogous to **b**–**e**. MSCs were identified via high CXCL12 expression (**g**) and a CD11a^−^CD13^+^ gate on total BM cells was predicted for the isolation of CXCL12^+^ mesenchymal stem cells (**h**), which was confirmed using flow cytometry (**i**). **j**, Confirmation of differentially expressed surface markers on MSCs, derived from Abseq data, by flow cytometry. **k**, Heatmap depicting gene expression of common hematopoietic and MSC signature genes in FACS-sorted CD11a^−^CD13^+^ MSCs and total BM cells outside the gate, as quantified by qPCR (*n* = 3 patients). Source data

**Extended Data Fig. 8:**
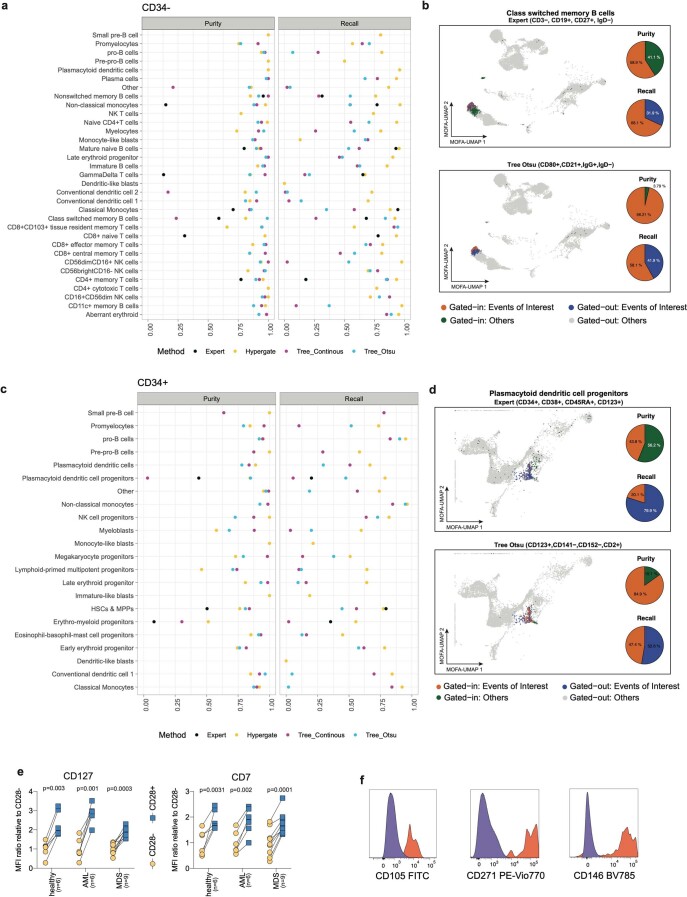
Comparison of data-defined and state-of-the-art (expert-defined) gating schemes. *Related to* Fig. 5. **a**. Performance of different methods for the definition of gates of CD34- populations. Gates for each cell type were defined from CD34- Abseq data as follows: Black dots correspond to gates identified from literature (Supplementary Table 5). Yellow dots correspond to gates that were set using the hypergate algorithm (Becht et al., 2019). Light blue and violet dots correspond to gates that were set using a decision tree with or without predefined thresholds, respectively. See also Methods. For each gating scheme, precision (purity) and recall were calculated. **b**. Automated and expert-defined gates of class switched memory B cells. Orange and blue dots on the UMAP correspond to class switched memory B cells located within and outside of the selected gate, respectively (that is true positives and false negatives). Green and gray dots correspond to other cells located inside and outside the gate, respectively (that is false positives and true negatives). Pie charts indicate precision and recall. Top: Shows an expert-defined state of the art gating scheme (CD3-CD19 + CD27 + IgD-). Bottom: Shows a data-defined gating scheme (CD80 + CD21 + IgG+IgD-). **c**. Like *a*, except that CD34 + populations are shown. **d**. Like *b*, except that gating schemes to define pDC progenitors are shown. **e**. Paired scatter plot depicting the mean fluorescence intensities (MFI) of CD127 and CD7 in CD4 + CD28- cytotoxic CD4 + T cells (yellow) and CD4 + CD28 + other CD4 + T cells (blue) in BM samples from healthy, AML and MDS patients. n = 6, 6 and 9 patients in the respective groups. **f**. Representative FACS histograms showing surface expression of well-known MSC surface markers. No significance = ns, P < 0.05 *, P < 0.01 **, P < 0.001 ***, P < 0.0001 ****. CD4 + CD28- and CD4 + CD28 + paired cell populations within the same BM donors from different disease entities were compared using paired two-tailed t-test. P-values were adjusted for multiple comparisons using the Bonferroni method. Source data

Gating strategies for flow cytometry have evolved historically in a process of trial and error. In particular, the isolation of rare and poorly characterized cell subsets using flow cytometry remains challenging, whereas commonly used gating schemes are not necessarily optimal in purity (precision) and efficiency (recall). To tackle these problems, we explored different machine learning approaches for the data-driven definition of gating schemes. For all populations in our dataset, gating schemes defined by machine learning approaches provided higher precision (purity) when compared with classical gating schemes from the literature (Fig. 5a, Extended Data Fig. 8a–d and Supplementary Table 5). While different machine learning methods tested achieved similar purities, gates defined by the hypergate algorithm^34^ offered a higher recall (Fig. 5a and Extended Data Fig. 8a–d).

To validate and demonstrate this approach, we focused on determining new gating strategies for rare and poorly characterized BM cell types, such as cytotoxic CD4^+^ T cells (Fig. 5b) and MSCs (Fig. 5g). Cytotoxic CD4^+^ T cells represent a rare T cell population characterized by the expression of cytotoxicity genes typically observed in their well-characterized CD8^+^ T cell counterparts^35^. While this cell type has been suggested to be involved in several physiological and pathophysiological processes, no coherent gating strategy for their prospective isolation exists^36^. Hypergate suggested that cytotoxic CD4^+^ T cells display an immunophenotype of CD4^+^CD28^−^, and differential expression analyses of surface markers revealed that cytotoxic CD4^+^ T cells express significantly lower levels of CD7, CD25, CD127 and CD197 when compared with other CD4^+^ T cell subsets (Fig. 5b–e). Flow cytometric analyses of CD4^+^CD28^−^ T cells confirmed the expected immunophenotype in BM from healthy donors and patients with different hematological cancers, suggesting a robust and efficient prospective isolation of this rare cell type (Fig. 5d and Extended Data Fig. 8e). Finally, FACS-based sorting of CD4^+^CD28^−^ T cells followed by gene expression analysis confirmed the expression of cytotoxicity genes in this population (Fig. 5f).

MSCs constitute a rare and heterogeneous group of cells in the BM^37,38^. While ex vivo expanded MSCs have been phenotyped extensively, primary human MSCs remain poorly characterized, in particular due to their extremely low frequency. In our dataset, we captured a small number of heterogeneous MSCs, with one subset (MSC-1) expressing high levels of the key BM-homing cytokine CXCL12 (Fig. 5g). Hypergate suggested *CXCL12*-expressing MSCs to be isolated most efficiently by expression of CD13 and absence of CD11a (Fig. 5h). Indeed, flow cytometric analyses of CD13^+^CD11a^−^ MSCs validated the immunophenotype suggested by our Abseq data and confirmed known and new MSC surface markers identified by our approach (Fig. 5i,j and Extended Data Fig. 8f). Moreover, FACS-based isolation of CD13^+^CD11a^−^ cells followed by transcriptomic analyses revealed a high enrichment of *CXCL12* and other key MSC signature genes (Fig. 5k).

Together, these analyses demonstrate the utility of our approach for deriving gating schemes from data and mapping the surface marker expression of poorly characterized populations. In combination with our single-cell proteo-genomic reference map, the Abseq App allows users to define new data-driven gating schemes for any population of interest.

### A data-defined gating scheme for human hematopoiesis

Gating schemes for complex biological systems, such as the HSPC compartment, are improving steadily. However, there is strong evidence from single-cell transcriptomics^9,10,18,19^, lineage tracing^22,23^ and single-cell functional experiments^21^ that even the most advanced gating schemes do not recapitulate the molecular and cellular heterogeneity observed by single-cell genomics approaches. This has contributed to several misconceptions in the understanding of the hematopoietic system, most notably incorrect assumptions on the purity of cell populations and inconsistent views on lineage commitment hierarchies^11–14^.

**Fig. 6:**
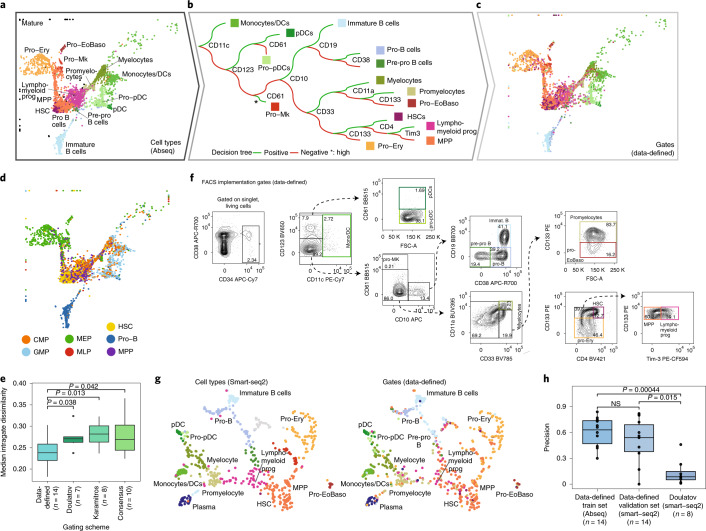
Data-driven definition of gating schemes for HSPCs. **a**, UMAP depicting all CD34^+^ HSPCs cells from one healthy young individual. See **b** for color scheme. **b**, Decision tree using surface marker expression from the Abseq data to classify cells into cell types. See Methods and main text for details. **c**, UMAP highlighting cell type classification obtained from the decision tree. Colors correspond to ‘gates’ applied to the expression levels of the 12 markers shown in **b**, not gene expression clusters. **d**, UMAP highlighting classification obtained from a decision tree recapitulating the classical gating scheme used in the field^17^. Since CD135 was not part of the Abseq panel, the expression of *FLT3* was smoothed using MAGIC^48^. **e**, Boxplot depicting the intragate dissimilarity for cell classification with panels from Doulatov et al.^17^, the gating scheme from Karamitros et al.^25^, a ‘consensus gating’ scheme (*see* Extended Data Fig. 9) and the data-driven gating scheme (**c**). Intragate dissimilarity is defined as one minus the average Pearson correlation of normalized gene and surface antigen expression values of all cells within the gate. *P* values are from a two-sided Wilcoxon test. Sample size is shown in the figure. See Methods, section Data visualization for a definition of boxplot elements. **f**, Implementation of FACS gating scheme from **b**. **g**, UMAP display of mRNA expression of *n* = 630 CD34^+^ HSPCs from an indexed single-cell Smart-seq2 experiment where the expression of relevant surface markers was recorded using FACS. Left panel: color indicates gene expression cluster, see Supplementary Note 8 for details. Right panel: color indicates classification by the FACS scheme from **f**. **h**, Precision of the classification scheme shown in **b**, computed on the training data (Abseq) and the test data (Smart-seq2). Precision was computed per gate as the fraction of correctly classified cells. For comparison with the Doulatov gating scheme, the dataset from Velten et al.^9^ was used. NS, not significant. *P* values are from a two-sided Wilcoxon test. Sample size is shown in the figure.

**Extended Data Fig. 9:**
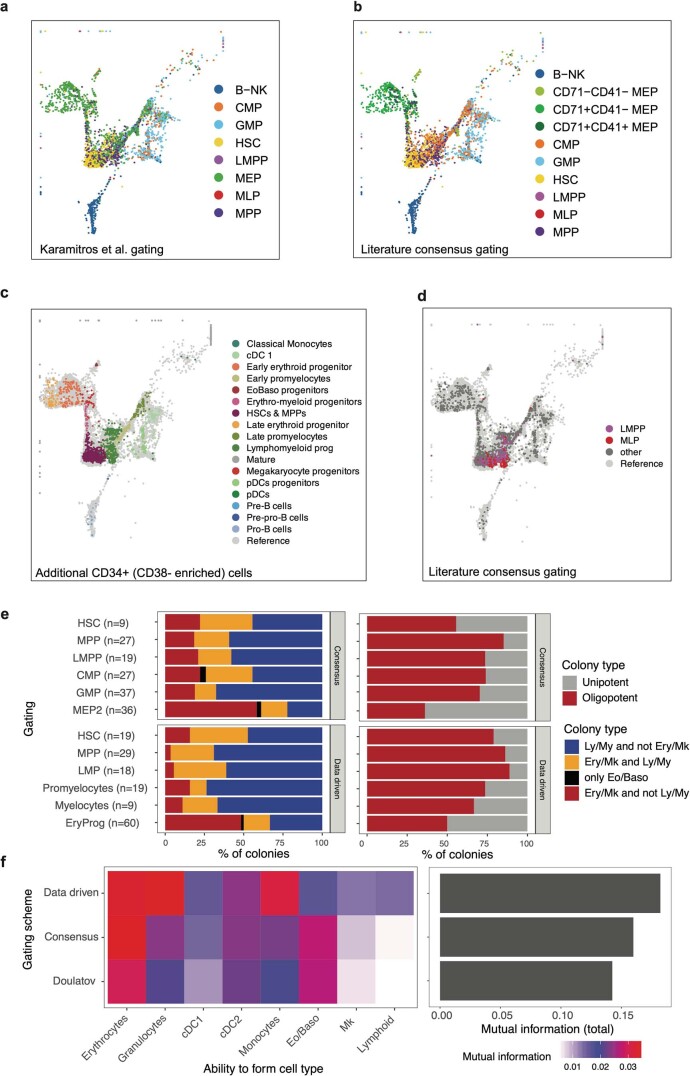
Evaluation of different gating schemes. Related to Fig. 6. **a**. UMAP highlighting classification obtained from the gating scheme described by Karamitros et al., 2018, that is HSC: CD34 + CD38-CD10-CD45RA-CD90 + ; MPP: CD34 + CD38-CD10-CD45RA-CD90-; LMPP:CD34 + CD38-CD10-CD45RA + ; MLP: CD34 + CD38-CD10 + ; MEP: CD34 + CD38 + CD10-CD45RA-CD123-; CMP: CD34 + CD38 + CD10-CD45RA-CD123 + ; GMP: CD34 + CD38 + CD10-CD45RA + CD123 + ; B-NK: CD34 + CD38 + CD10 + . **b**. UMAP highlighting classification obtained from a consensus scheme combining the schemes of Doulatov et al., Karamitros et al. and Psaila et al., HSC: CD34 + CD38-CD10-CD45RA-CD90 + ; MPP:CD34 + CD38-CD10-CD45RA-CD90-; LMPP:CD34 + CD38-CD10-CD45RA + ; MLP: CD34 + CD38-CD10 + ; CD71-CD41- MEP: CD34 + CD38 + CD10-CD45RA-*FLT3*-*ITGA2B*-*TFRC-*; CD71 + CD41- MEP: CD34 + CD38 + CD10-CD45RA-*FLT3*-*ITGA2B*-*TFRC* + ; CD71 + CD41 + MEP: CD34 + CD38 + CD10-CD45RA-*FLT3*-*ITGA2B* + ; CMP: CD34 + CD38 + CD10-CD45RA-*FLT3* + ; GMP: CD34 + CD38 + CD10-CD45RA + ; B-NK: CD34 + CD38 + CD10 + . The marker CD135, CD41, CD71 were not part of the 97 Abseq panel. The expression of the corresponding genes, *FLT3*, *ITGA2B* and *TFRC*, were smoothened using MAGIC respectively (van Dijk et al., 2018). **c**. UMAP of additional CD34 + cells with specific enrichment of CD34 + CD38- cells, projected on the original coordinate system, colored by mapped cell types **d**. Same as *c* but colored by immunophenotypic classification obtained from a consensus scheme recapitulating the scheme of Karamitros et al. and Psaila et al. (see above). **e**. Separation of functional potential by the data-driven and the literature ‘consensus gating’ scheme. Single cells were sorted according to the two gating schemes and cultured for 19 days. Colonies were scored as Ery/Mk if they contained at least 5 erythroid or megakaryocytic cells, and as Ly/My if they contained at least 5 cells of types Neutrophil, cDC, Monocyte, or B/NK. Unipotent: Only one of these cell types was formed with at least 5 cells; oligopotent: At least two of these cell types were formed. Only gates for which at least 9 colonies were observed are shown. **f**. Mutual information (in nats) between the gate identity and the ability to form any of the cell types, or the total mutual information across all cell types.

To generate flow cytometric gating schemes that most adequately reflect the transcriptomic states associated with HSC differentiation, we used the Abseq dataset of CD34^+^ cells from one BM sample (‘Young1’) to train a decision tree. Thereby, we obtained a gating scheme that uses 12 surface markers to define 14 leaves representing molecularly defined cell states with high precision (Fig. 6a–c). The data-derived scheme excelled in the identification of lineage-committed progenitors—a principal shortcoming of many current gating strategies (Fig. 6a–c)^9,10,21,22^. Importantly, cell populations defined by the data-defined gating scheme were transcriptionally more homogenous, compared with a widely used gating scheme^17^ (Fig. 6d,e), a state-of-the-art gating scheme focusing on lymphomyeloid differentiation^25^ (Fig. 6e and Extended Data Fig. 9a–d) and a ‘consensus gating’ scheme generated in silico to combine the latter with a scheme focusing on erythroid-myeloid differentiation^26^ (Fig. 6e and Extended Data Fig. 9b). Of note, individual populations from the data-defined scheme displayed a functional output comparable with that of populations of the ‘consensus gating’ scheme, while the data-defined scheme overall provided a higher level of information on functional lineage commitment (Extended Data Fig. 9e,f).

To validate this new gating scheme, we implemented the suggested surface marker panel in a classical flow cytometry setup and performed Smart-seq2-based single-cell RNA-seq while simultaneously recording surface marker expression (index scRNA-seq) (Fig. 6f,g and Supplementary Note 8). This approach demonstrated that the new gating strategy efficiently separated molecularly defined cell states (Fig. 6g). Quantitatively, the data-defined gating scheme performed equally well at resolving molecularly defined cell states on the Abseq training data as on the Smart-seq2 validation data, and significantly outperformed the expert-defined gating scheme (Fig. 6h). A limitation of the low cellular throughput of the Smart-seq2 analysis is that the signature-based identification might result in the ‘over-identification’ of certain cell states. Together, our results demonstrate that high-content single-cell proteo-genomic maps can be used to derive data-defined cytometry panels that describe the molecular states of complex biological systems with high accuracy. Moreover, our gating scheme permits a faithful identification and prospective isolation of transcriptomically defined progenitor states in the human hematopoietic hierarchy using cost-effective flow cytometry.

### Mapping flow cytometry data on single-cell reference maps

**Fig. 7:**
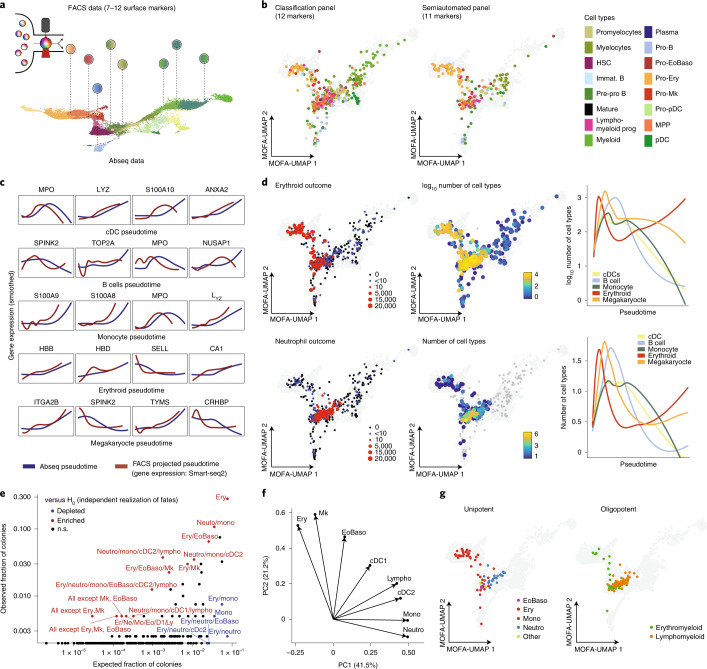
Systematic integration of single-cell genomics, flow cytometry and functional data. **a**, Illustration of the concept. **b**, Projection of indexed Smart-seq2 data onto a reference UMAP. Single cells with recorded FACS measurements of surface markers were subjected to Smart-Seq2 based scRNA-seq. FACS measurements of surface markers were used to project cells onto the UMAP (Methods). Colors denote cell type identified from RNA-seq. See Supplementary Table 6 for composition of the FACS panels. **c**, FACS-based projection of indexed Smart-seq2 data onto reference pseudotime trajectories. Line plots depict the RNA expression of differentiation markers smoothed over projected pseudotime values (red). For comparison, expression values determined from Abseq data are shown (blue). The selected genes correspond to the five genes with the strongest statistical association with the respective trajectory. **d**, Projection of indexed single-cell culture data onto a reference UMAP. Single cells with available FACS measurements of 12 surface markers were projected onto the UMAP defined by Abseq. Single cells were seeded into culture medium supporting the formation of erythroid, megakaryocytic and distinct myeloid cell types. UMAPs highlight the ability of single cells to give rise to erythroid cells and neutrophils, colony size and total number of cell types per colony. Colony and total number of cell types per colony are also plotted against projected pseudotime. **e**, Analysis of cell type combinations in *n* = 397 colonies. For any combination of Erythroid (Ery), Neutrophil (Neutro), Monocytic (Mono), Eosinophil or Basophil (EoBaso), Lymphoid (Lympho), Megakaryocytic (Mk) and Dendritic (cDC1 and cDC2) potential, the scatter plot depicts the fraction of colonies containing this exact combination of cell types (*y* axis) and the theoretical fraction of colonies containing the same combination under the assumption that cell fates are independently realized with the same marginal probabilities (*x* axis). Significance from a binomial test is color-coded. n.s., not significant. These analyses do not exclude that other combinations of fates are not biologically selected as well; that is, absence of evidence does not constitute evidence for absence. **f**, Principal component analysis of colony compositions. PC, principal component. **g**, Distribution of colonies with frequent combinations of cells types in the projected UMAP space. Erythromyeloid, exclusively EoBaso, Mk and/or Ery cells; Lymphomyeloid, all other combinations.

**Extended Data Fig. 10:**
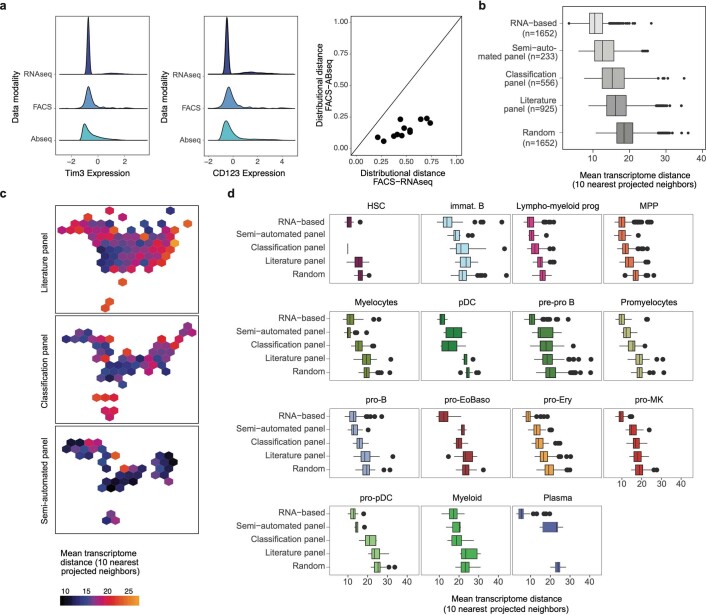
Projection and classification of cytometry data using a single-cell proteo-genomic reference. Related to Fig. 7. **a**. Distribution of normalized, scaled expression values of Tim3 (left panel) and CD123 (central panel) measured by scRNA-seq, Abseq, and FACS. Right panel: Scatter plot depicts the dissimilarity between the distribution of expression values measured by FACS, and the distribution measured by scRNA-seq (x-axis) or Abseq (y-axis) as quantified using Kolmogorov-Smirnov distance. Data for all markers included in the panel from main Fig. 6f is shown. **b**–**d**. Comparison of data integration strategies. Smart-seq2 data and Abseq data were integrated with five different strategies. RNA-based: Integration by Seurat v3, based on gene expression (transcriptome). Random: Random selection of ten nearest neighbors. Others: Surface marker-based integration using NRN, using defined sets of surface markers (Classification panel, Semi-automated panel: see Supplementary Table 6. Literature panel: CD34, CD38, CD45RA, CD90, CD10, CD135/*FLT3*, CD49f). For every cell projected on the UMAP, the ten nearest neighbors in projected UMAP space were identified. Subsequently, the mean Euclidean distance between their location in a gene expression-based PCA space (Smart-seq2) was computed. Sample size n = 1652. **b**. Boxplot summarizing the distance across data integration strategies. See figure for sample size. See Methods, section ‘*Data visualization* for a definition of boxplot elements’. **c**. Hexagonal plot summarizing the projection accuracy for different regions of the UMAP. **d**. Boxplots stratified by cell type demonstrate that projection using the semiautomated panel performs close to an RNA-based integration in most cases. See panel b for sample size.

While classical FACS gating strategies are of great use for the prospective isolation and characterization of populations, single-cell genomics studies revealed that differentiation processes, including the first steps of hematopoiesis, are represented most accurately by a continuous process^9,18,20,27,39^. To complement the approach based on discrete gates, we propose here that high-dimensional flow cytometry data can be used to place single cells into the continuous space of hematopoietic differentiation spanned by single-cell proteo-genomics exploiting shared surface markers (Fig. 7a). Based on the observation that surface marker expressions in flow cytometry and Abseq follow similar distributions (Extended Data Fig. 10a), we developed a new projection algorithm termed nearest rank neighbors (NRN https://git.embl.de/triana/nrn/; see Methods). Given an identical starting population, NRN employs sample ranks to transform surface marker expression of FACS and Abseq data to the same scale, followed by k-nearest neighbors-based projection into a space defined by the proteo-genomic single-cell data. We tested NRN on FACS-indexed Smart-seq2 datasets using the classification panel developed in Fig. 6 (12 markers) and a semiautomated panel based on our Abseq data to better resolve erythromyeloid lineages (11 markers; Supplementary Note 8). We evaluated the performance of NRN using a variety of methods. First, cell types molecularly defined by Smart-seq2 were placed correctly on the Abseq uniform manifold approximation and projection (UMAP) (Fig. 7b). For most molecularly defined cell types, the accuracy of the projection using the flow cytometry data was close to the performance of data integration using whole transcriptome data with a state-of-the-art algorithm (Extended Data Fig. 10b–d). Most importantly, the projections closely reflected the gradual progression of cells through pseudotime, as confirmed by the expression dynamics of key lineage genes from our FACS-indexed Smart-seq2 data (Fig. 7c). This suggests that NRN, in combination with high-quality reference datasets, can be used to study the continuous nature of cellular differentiation processes by flow cytometry.

A key limitation of single-cell genomics remains the lack of insight into functional differentiation capacities of cells. We therefore evaluated whether NRN can be used to interpret functional single-cell data in the context of single-cell genomic reference maps. For this purpose, we performed single-cell culture assays, while recording surface markers of our data-defined gating scheme from Fig. 6, followed by data integration using our Abseq data via NRN. As expected, cells with the highest proliferative capacity and lineage potency were placed in the phenotypic HSC and MPP compartments, and HSPCs placed along the transcriptomically defined differentiation trajectories continuously increased the relative generation of cells of the respective lineage (Fig. 7d). Functionally unipotent progenitors cells were observed along the respective transcriptomic trajectories, but were also present in the phenotypic HSC/MPP compartment (Fig. 7d,g), in line with previous findings on early lineage commitment of HSPCs^9,10,21^. By contrast, oligopotent cells with distinct combinations of cell fates were enriched specifically in the HSC/MPP compartment (Fig. 7d,g). Some of these fate combinations, in particular combinations of erythroid, megakaryocytic and eosinophilic/basophilic fates, and combinations of lymphoid, neutrophilic, monocytic and dendritic fates, co-occurred more frequently than expected by chance (Fig. 7e,f), in line with most recent findings on routes of lineage segregation^9,18,40,41^. Despite strong associations between surface phenotype, transcriptome and function, cells with a highly similar phenotype can give rise to different combinations of lineages (Fig. 7g). This observation suggests a role of stochasticity in the process of lineage commitment, or hints towards layers of cell fate regulation not observed in the transcriptome. Together, our observations confirm that hematopoietic lineage commitment occurs predominantly continuously along the routes predicted by the transcriptome, with an early primary erythromyeloid versus lymphomyeloid split^9,10,18,21,40,41^ and might help reconciling discrepancies in the interpretation of previous studies.

In summary, our data resource, alongside the NRN algorithm, enables accurate integration of flow data with single-cell genomics data. This permits the charting of continuous processes by flow cytometry and the mapping of single-cell functional data into the single-cell genomics space.

## Discussion

In this study, we have demonstrated the power of single-cell proteo-genomic reference maps for the design and analysis of cytometry experiments. We have introduced a map of human blood and BM spanning the expression of 97–197 surface markers across 45 cell types and stages of HSC differentiation, healthy ageing and leukemia. Our dataset is carefully annotated and will serve as a key resource for hematology and immunology.

While cytometry experiments remain the workhorse of immunology, stem-cell biology and hematology, recent single-cell atlas projects have revealed that current cytometry setups do not accurately reflect the full complexity of biological systems^10,42^. For the first time, we have exploited single-cell proteo-genomic data to systematically design and interpret flow cytometry experiments that mirror most accurately the cellular heterogeneity observed by single-cell transcriptomics. Unlike approaches based on index sorting^9,10,43,44^, single-cell proteo-genomics has a sufficient throughput to enable the profiling of entire tissues or organs, and at the same time covers up to several hundred surface markers. Unlike single-cell RNA-seq data, antibody tag counts reflect the true distribution of surface marker expression, enabling a quantitative integration of cell atlas data with FACS. Building on these unique properties of our reference map, we have automated the design of gating schemes for the isolation of rare cell types, devised a gating strategy that reflects the molecular routes of HSC differentiation and demonstrated the direct interpretation of flow cytometry data in the context of our reference.

These advances enable a functional characterization of molecularly defined cell states and thereby directly affect HSC research. There is a growing consensus in the field that lineage commitment occurs early from primed HSCs, that not all progenitor cells in the classical megakaryocyte-erythrocyte progenitor/granulocyte-macrophage progenitor (MEP/GMP) gates are functionally oligopotent and that the main branches of the hematopoietic system are a *GATA2*-positive branch of erythroid, megakaryocytic and eosinophil/basophil/mast cell progenitors, as well as a *GATA2*-negative branch of lymphomyeloid progenitors, including the progenitors of monocytes, neutrophils and dendritic cells^9,18,19,27,40,41,45^. Due to a lack of better alternatives, many functional studies still use the classical gating scheme alongside the outdated concept of ‘common myeloid progenitors’^15,16,28^. Here, we introduce and validate a flow cytometry scheme that allows the prospective isolation of molecularly homogeneous progenitor populations. We have used this scheme to show that transcriptional lineage priming impacts on cellular fate in vitro^9,21^, thereby contributing further evidence for the revised model of hematopoiesis. In the future, a wider use of this scheme has the potential to avoid conflicting results stemming from imprecisely defined populations.

Furthermore, these advances enable the rapid profiling of blood formation and other BM phenotypes while offering a resolution comparable with that of single-cell genomics. Recently, BM phenotypes of disease, ranging from sickle cell disease^46^ to leukemia^47^ have been investigated using scRNA-seq. However, due to economic and experimental hurdles, the throughput of these studies has remained restricted to maximally tens of patients. Accordingly, the ability to associate patient genotypes with phenotypes is thereby highly limited, and these assays have not been translated to diagnostic routines. Our new gating schemes and analytical strategies are widely applicable to profile aberrations encountered in disease, both in research and, ultimately, in clinical diagnostics.

Although we have demonstrated the implementation of data-driven design and analysis strategies for cytometry assays in the context of BM, conceptually the approach presented here can be applied to any organ of interest. Thereby, it has the potential to enable the precise isolation and routine profiling of myriad cell types discovered by recent single-cell atlas projects.

## Methods

All reagents and antibodies used are listed in Supplementary Tables 1 (primers for targeted transcriptomics), 2 (Abseq antibodies) and 6 (all other reagents, oligonucleotides, equipment and software).

### Human samples

BM samples from healthy and diseased donors were obtained at the University clinics in Heidelberg and Mannheim after informed written consent using ethic application numbers S480/2011 and S-693/2018. For demographic characteristics on sample donors, see Supplementary Table 3. BM aspirates were collected from iliac crest. Healthy BM donors received financial compensation in some cases. For BM, mononuclear cells were isolated by Ficoll (GE Healthcare) density gradient centrifugation and stored in liquid nitrogen until further use. All experiments involving human samples were approved by the ethics committee of the University Hospital Heidelberg and were in accordance with the Declaration of Helsinki.

### Cell sorting for Abseq

Human BM samples were thawed in a water bath at 37 °C and transferred dropwise into RPMI-1640 10% FCS. Cells were centrifuged for 5 min at 350 and washed once with RPMI-1640 10% FCS. Cells were resuspended in FACS buffer (FB) (PBS 5% FCS 0.5 mM EDTA) containing CD34-PE and CD3 PE-Cy7 and FcR blocking reagent (Miltenyi) and incubated for 15 min at 4 °C. Cells were washed with FB and resuspended in 1 ml FB, followed by addition of 1 µl CellEvent Caspase-3/7 Green (ThermoFisher) and 1 µl 4,6-diamidino-2-phenylindole (DAPI) (ThermoFisher) to the cell suspension. After 3 min incubation at room temperature, cells were filtered through a 40 µm cell strainer. Singlet, CaspaseGreen^−^ DAPI^−^ total BM and singlet, CaspaseGreen^−^ DAPI^−^ CD34^+^ (HSPCs) as well as singlet, CaspaseGreen^−^ DAPI^−^ CD3^+^ (T cells) cells were sorted on an Aria Fusion II cell sorter (BD). In general, the entire CD34^+^ fraction from one thawed vial was sorted (~2 × 10^4^) and combined with 1 × 10^5^ CD34^−^ total BM cells (see also Extended Data Fig. 2). In CD3^+^ T cell-enriched AML samples, 2 × 10^4^ CD3^+^ T cells were mixed with the CD34^+^ HSPC fraction and combined with 1 × 10^5^ CD34^−^ total BM cells. For the generation of the AML query datasets, 2 × 10^4^ live total BM cells from each of 12 different AML samples were sorted. In case of the CD34^+^ immature HSPCs enrichment experiment, healthy adult human BM cells were stained with anti-human CD34, CD38, CD45RA, CD10 and fixable viability dye efluor506 and 5 × 10^3^ were sorted from each of four different gates (CD34^+^CD38^+^CD45RA^−^, CD34^+^CD38^+^CD45RA^+^, CD34^+^CD38^−^CD45RA^−^, CD34^+^CD38^−^CD45RA^+^). In cases where different biological samples or sorted populations were combined in the same run, cells of interest were sorted and labeled by cell hashing antibodies before surface labeling and single-cell capture as described in Abseq surface labeling, single-cell capture and library preparation.

### Cell sorting for gene expression analysis and flow cytometry

Human BM samples were thawed as described above. For dead cell exclusion and blocking of nonspecific binding, fixable viability dye efluor506 (ThermoFisher) and FcR blocking reagent (Miltenyi) were used in all staining solutions. Cells were generally stained for 15 min at 4 °C and then washed once with FB, resuspended in 1 ml FB and filtered through a 40 µm cell strainer. For cytotoxic CD4^+^ T cell sorting, cells were stained in FB containing anti-CD3, CD4, CD7, CD28, CD45RA, CD45 and CD127 surface antibodies. Singlet, live, CD45^+^, CD3^+^ cells were gated and CD4^+^CD28^−^ or CD4^+^CD28^+^ cells were sorted and processed as described below. For MSC gene expression analysis, cells were stained in FB containing anti-CD10, CD11a, CD13, CD26, CD31, CD45, CD49a, CD90, CD105, CD146 and CD271 surface antibodies. Singlet, live, CD11a^−^CD13^+^ MSCs or all cells outside this gate were sorted. Cells were sorted on either FACSAria Fusion or FACSAria II equipped with 100 µm nozzles, respectively.

For flow cytometric analysis, human BM samples were processed as described above. For analysis of cytotoxic CD4 T cells across hematopoietic malignancies, cells were stained with anti-CD3, CD4, CD7, CD25, CD28, CD45RA, CD45, CD69 and CD127 surface antibodies. For analysis of CD98 expression in hematopoietic stem and progenitors, cells were stained with anti-human CD4, CD10, CD11a, CD34, CD38, CD45RA, CD49f, CD90, CD98, CD133 and Tim3 antibodies. For analysis of CD326 surface expression in comparison with CD71 and CD41, healthy adult human BM was stained with anti-human CD34, CD38, CD41, CD44, CD45RA, CD49b, CD49d, CD71, CD90 and CD123 antibodies. All experiments were measured on BD FACSFortessa flow cytometers, equipped with five lasers.

### Panel design for targeted transcriptomics

Panel design is described in Supplementary Note 1. In short, we used a human cell atlas reference and followed the method described by Schraivogel et al. for target gene selection^49^.

### Abseq surface labeling, single-cell capture and library preparation

Abseq surface antibody libraries (Supplementary Table 2) were pipetted 24 h before experiments. For most antibodies, 1 µl was used for surface library preparation. Antibodies recognizing epitopes with well-known high surface expression were further diluted in PBS and 1 µl was added to the surface library (for example HLA ABC, CD45, CD11a). Sorted cells (around 1.2 × 10^5^–1.4 × 10^5^; described in Cell sorting for Abseq) were centrifuged 5 min at 350*g* and resuspended in the surface library mix (around 100 µl for the 97 Ab panel, 200 µl for the 197 Ab panel). In cases where different biological samples or sorted populations were combined in the same run, sorted cells were labeled individually with oligonucleotide coupled cell hashing antibodies (BD single-cell multiplexing kit) for 25 min on ice, washed three times in all, each followed by 5 min centrifugation at 350*g* and then pooled and then subjected to Abseq cell surface labeling. Cells were then labeled for 30 min at 4 °C and washed three times in all, each followed by 5 min centrifugation at 350*g*. Cells were resuspended in sample buffer (BD Rhapsody Cartridge reagent kit) and between 1 × 10^4^ and 2 × 10^4^ cells were captured with the BD Rhapsody single-cell system following the manufacturer’s instructions^50^. Antibody tag libraries, multiplexing libraries and targeted mRNA gene expression libraries were generated following manufacturer instructions. For mRNA libraries, the targeted panel (Supplementary Table 1) or the whole transcriptome analysis library preparation protocol was used according to the manufacturer’s instructions (BD). Resulting libraries were quality checked by Qubit and Bioanalyzer, pooled and sequenced using NextSeq500 or Illumina Novaseq S2 (Illumina; high-output mode).

### Single-cell index cell cultures

Two days before index sorting, irradiated MS-5 feeder cells were plated at a density of 1 × 10^4^ cells per well into 96-well flat-bottom cell culture plates in αlpha-minimal essential medium with ribo-and deoxynucleosides (ThermoFisher) containing 10% FCS (Gibco), glutamine (2 mM) (ThermoFisher), penicillin/streptomycin (100 U ml^−1^) (ThermoFisher) and sodium pyruvate (2 mM) (Gibco). Several hours before index sorting, the medium was replaced by 100 µl H5100 medium (StemCell Technologies) containing glutamine (2 mM) (ThermoFisher), penicillin/streptomycin (100 U ml^−1^) (ThermoFisher), hydrocortisone (1 nM) (StemCell Technologies), SCF (20 ng ml^−1^), FLT3-L (100 ng ml^−1^), TPO (50 ng ml^−1^), IL-3 (20 ng ml^−1^), IL-5 (20 ng ml^−1^), IL-6 (20 ng ml^−1^), IL-7 (20 ng ml^−1^), IL-11 (20 ng ml^−1^), G-CSF (20 ng ml^−1^), GM-CSF (20 ng ml^−1^), M-CSF (20 ng ml^−1^) (all Preprotech) and EPO (3 U ml^−1^) (R&DSystems). Two BM samples from the same donor were thawed and washed as described above. The first sample was subsequently resuspended in 100 µl FB containing anti-human CD4, CD10 (BioLegend), CD11a, CD11c, CD19, CD33, CD34, CD38, CD61, CD123, CD133 and Tim3 antibodies (Classification panel), whereas the second sample was stained with anti-human CD11a, CD33, CD34 (Biolegend), CD38, CD49b, CD61, CD71, CD123, CD133, CD326 and FcεR1A (eBioscience) (Semiautomated panel). In another experiment, cells were labeled with anti-human CD11a, CD71, CD45RA, CD44, CD135, Tim3 (Biolegend), CD90, CD326, CD41 (BioLegend), CD123 (ThermoFisher), CD10, CD38 and CD34 (BioLegend) antibodies (Consensus panel). All antibody clones for flow cytometry matched clones from Abseq experiments and were purchased from BD, except otherwise indicated. For dead cell exclusion and blocking of nonspecific binding, fixable viability dye efluor506 (ThermoFisher) and FcR blocking reagent (Miltenyi) were included in both staining solutions. After staining for 15 min at 4 °C, cells were washed with FB, resuspended in 1 ml FB and filtered through a 40 µm cell strainer. For both assays, 480 single, live CD34^+^ cells were FACS indexed and sorted into the feeder cell containing 96-well plates as described above. Cells were incubated at 37 °C, 5% CO_2_ for 16–19 days. To analyze clonal output, cells were harvested and transferred to 96-well V bottom plates, washed with FB and resuspended in 10 µl FB containing anti-human CD1c (Biolegend), CD14, CD19 (Biolegend), CD34 (Biolegend), CD41a (Biolegend), CD45, CD56, CD66b, CD123, CD235a, CD303, CD141, CD370 (Biolegend) and FcεR1a (eBioscience). For dead cell exclusion and blocking of nonspecific binding, fixable viability dye efluor506 (ThermoFisher) and FcR blocking reagent (Miltenyi) were included in the staining solution. After staining for 15 min at 4 °C, cells were washed with FB and resuspended in 100 µl FB and filtered through a 40 µm cell strainer. Cells were analyzed on a LSRII (BD) flow cytometer. Erythroid lineage output was determined via CD235^+^ expression, which was concomitant with the downregulation of CD45 expression (CD45^−^CD235^+^). Myeloid lineages were defined via CD66b and CD14 antibodies (CD235^−^CD45^+^CD66b^+^ or CD235^−^CD45^+^CD14^+^). Dendritic cell lineages were defined via CD1c, CD141, CD370, CD303 and CD123 expression. Lymphoid cell lineages were defined via CD19 and CD56 expression. Megakaryocyte output was determined via CD41a expression, Eosinophil/basophil output was determined via FcεR1a expression. Generally, only wells that contained more than ten CD45^+^CD235^−^ or CD45^+^CD235^+^ or CD45^+^CD235^−^ cells were considered during analysis if not stated otherwise. For calculation of erythroid ratios, the count of all generated erythroid cells was divided by the sum of all other generated cells. Myeloid ratios were determined by dividing the sum of generated myeloid and dendritic cells by the sum of all other generated cells.

### Single-cell index RNA-sequencing

For single-cell index RNA-sequencing, cells from the same samples that were prepared for single-cell cell index cultures were used. Hardshell 96-well polymerase chain reaction (PCR) plates (Bio-Rad) were prefilled with 4 µl lysis buffer containing 1 µl RNase inhibitor (40 U ml^−1^, Takara), 1.9 µl Triton X-100 (0.2%, Sigma), 1 µl oligo dT_30_VN (10 µM, Sigma) and dNTPs (10 mM, ThermoFisher). Cells were FACS indexed, sorted into lysis buffer and snap frozen on dry ice. For cell lysis, plates were incubated for 5 min at 10 °C, followed by incubation for 3 min at 72 °C in a thermocycler (PCRMax). For reverse transcription, 0.25 µl RNase inhibitor (40 U ml^−1^, Takara), 0.5 µl DTT (20 mM, Takara) 0.2 µl template switching oligonucleotides (50 µM, IDT), 1.05 µl H_2_O (Ambion), 2 µl Smartscribe buffer (5×, Takara) and 1 µl Smartscribe (100 U ml^−1^, Takara) was added to each well. Reverse transcription was performed by incubating plates for 90 min at 42 °C, followed by ten cycles of 2 min at 50 °C, 2 min 42 °C, followed by 10 min at 72 °C followed by 4 °C storage. To amplify cDNA, 12.5 µl KAPA HiFi HotStart (Roche), 0.25 µl ISPCR primer (10 µM, Sigma) and 2.25 µl H_2_O was added to each well. Plates were incubated for 3 min at 98 °C, 23 cycles of 20 s at 98 °C, 15 s at 67 °C, 6 min at 72 °C followed by one stage for 5 min at 72 °C, followed by final storage at 4 °C. cDNA was then cleaned up using an equal volume (25 µl) of SPRIselect beads (Beckman) and tagmented using homemade Tn5^51^. Resulting libraries were quality checked by Qubit and Bioanalyzer, pooled and sequenced using all lanes in an Illlumina Hiseq 4000.

### Real-time-quantitative PCR

For real-time-quantitative PCR (RT-qPCR) analysis, cells of interest were sorted directly into RNA lysis buffer (Arcturus PicoPure RNA Isolation Kit, Life Technologies, Invitrogen), snap frozen and stored at −80 °C or processed directly for cDNA synthesis using SuperScript VILO cDNA synthesis kit (Invitrogen) according to the manufacturer’s instructions. Depending on the sorted cell number, cDNA was further diluted 1:5–1:10 in RNase-free water and 6 µl was mixed in technical triplicates in 384-well plates with 0.5 µl of forward and reverse primer (10 µM) and 7 µl PowerUP SybrGreen Mastermix (Thermo Fisher). Program: 50 °C for 2 min, 95 °C for 10 min and 40 cycles of 95 °C for 15 s, 60 °C 1 min. Primers were designed to be intron spanning whenever possible using PrimerBlast (National Center for Biotechnology Information) and purchased from Sigma Aldrich (purification: desalting). Experiments were performed on the ViiA7 System (Applied Biosystems) and analysis of gene amplification curves was performed using the Quant StudioTM Real-Time PCR Software v.1.3 (Applied Biosystems). RNA expression was normalized to the housekeepers glyceraldeyde-3-phosphate dehydrogenase and beta actin for gene expression analysis. Relative expression levels (2^−ΔCt^, ∆Ct = (geometric mean Housekeeper Ct)−(gene of interest Ct)) of replicates were log_10_ transformed and *z*-scored. Primers used in this study can be found in Supplementary Table 6.

### Analysis of Abseq data

Fastq files were processed via the standard Rhapsody analysis pipeline (BD Biosciences) on Seven Bridges (https://www.sevenbridges.com) according to the manufacturer’s recommendations. The resulting unique molecular identifier (UMI) count matrices were imported into R (v.3.6.2) and processed with the R package Seurat (v.3.1.3 and 3.2.0)^52^. To account for differences in sequencing depth across cells, both layers were normalized independently using Seurat defaults. RNA UMI counts were log-normalized, while antibody UMI counts were centered using log ratio normalization to account for unspecific binding background signal. Subsequently, both normalized matrices were concatenated and integration across patients was performed using Scanorama^53^. The resulting corrected counts were used for visualization and clustering analysis. Nonintegrated, raw counts were used for differential expression testing.

### Multiomics factor analysis integration, clustering and identification of cell type markers

Following integration, we removed genes and surface markers with variance near to zero using the caret package^54^ and used MOFA to perform data integration across modalities^55^. A total of 30 multiomics factor analysis (MOFA) factors were used as a starting point, with a drop factor threshold of 0.001. The resulting MOFA dimensions were used to construct a shared nearest neighbor graph and modularity-based clustering using the Louvain algorithm was performed. Finally, UMAP visualization was calculated using 30 neighboring points for the local approximation of the manifold structure. Marker genes and surface markers for every cell type were identified by comparing the expression of each in a given cluster against the rest of the cells using the receiver operating characteristic test. To evaluate, which genes classify a cell type, cell type specific markers were selected as those with the highest classification power defined by the area under the receiver operating characteristic curve.

### Processing of Smart-seq2 data

Count matrices were generated using pseudoalignment with Kallisto^56^ using the GRCh38 human reference genome as implemented in the Scater package v.1.14.6 (ref. ^57^). Gene level expression counts were imported into Seurat. Low-quality cells were removed on the basis of the percentage of mitochondrial RNA reads (>20%) and number of detected genes (<1,000). The remaining data were further processed using Seurat. Data was log-normalized and scaled. The top 2,000 highly variable genes were used for clustering and UMAP calculation. Cells were then annotated as described in Supplementary Note 8.

### Abseq App web application

Differential expression, data visualization and gating scheme calculation can be performed in the Abseq App shiny web application (https://abseqapp.shiny.embl.de/). The application was written in R and relies on the packages shiny and aws.s3. A demonstration video of the app is included as Supplementary Video 1.

### Pseudotime analysis

To reconstruct possible cell lineages from our single-cell gene expression data, data from individual samples were subset to include only the cell types from the CD34^+^ hematopoietic stem and progenitor compartment. MOFA–UMAP embedding was then used as input for pseudotime analysis by slingshot^58^. The HSC cluster was used as a start cluster, and myelocytes, class switched memory B cells, late erythroid progenitors, megakaryocyte progenitors and conventional dendritic cell compartments as the end clusters. The genes that significantly changed through pseudotime were determined by fitting a generalized additive model (GAM) for each gene, using the TradeSeq package^59^.

### Modeling variance in surface marker expression

To attribute the variance in surface marker expression to biological processes, we used the variancePartition package^60^ on log-transformed antibody read count data. As covariates, we used cell type annotation (for all cells except CD34^+^ HSPCs), splines with three degrees of freedom fitted through pseudotime (for CD34^+^ HSPCs, Pseudotime analysis), cell cycle scores (calculated using Seurat package defaults), scores for cytotoxicity and stemness (calculated using the gene lists in Supplementary Table 7 and the Seurat function AddModuleScore()), as well as technical covariates (number of genes observed, number of surface markers observed, reads on surface markers, reads on genes). To also account for variance explained by any hypothetical processes not in this predefined list, we additionally performed a factor analysis of the entire dataset (RNA plus surface markers) while accounting for the known covariates using ZiNB-WAVE^61^. We ran ZiNB-WAVE with four unknown factors on the concatenated mRNA and surface marker expression matrices while using a gene level-covariate specifying whether each row in the matrix is an mRNA or surface marker. The unknown factors explained only a very small part of the variance, and appeared to capture mostly differentiation processes not optimally explained by the pseudotime. Of note, markers with low absolute expression are more strongly subject to stochastic expression or measurement noise, while markers that are expressed by many different cell types are more strongly subject to technical effects, such as differences in single-cell library quality, likely due to the absence of true biological variability for these markers (Extended Data Fig. 5a). Other covariates are not affected by the expression level of the markers.

### Projection on a reference atlas

The projection on the reference dataset is described in Supplementary Note 7. In short, we used scMAP to calculate nearest neighbors and thereby determined cell type label, MOFA–UMAP coordinates and pseudotime value.

### Differential expression testing between experimental groups and estimation of interpatient variability

For comparing surface protein abundance between young and aged healthy as well as leukemic individuals, antibody tag read counts were summed at the level of cell types for each experimental batch (that is, donor). Differential expression testing was then performed for these pseudobulks using DESeq2 (ref. ^62^), either separately for each cell type (Fig. 4i, Extended Data Fig. 7c and Supplementary Data 2), or jointly across all cells while accounting for cell type as a covariate (Fig. 4b). For cell-type-specific comparisons, only samples for which the respective cell type was covered with at least 20 cells were included. When comparing leukemic with healthy individuals, age and gender were used as additional covariates. Unlike single-cell specific methods, DESeq2 estimates the variance in gene expression between experimental replicates to separate signal from noise while using a negative binomial distribution that is sufficiently generic to capture the count-nature data of antibody-based pseudobulk expression values.

To estimate the degree of interpatient variability of surface marker abundance while accounting for cell state differences, we trained random forest classifiers to predict the experimental batch (that is, donor) from gene expression separately for each cell state. The feature importance score from these classifiers was then scaled from zero to one and used to estimate interpatient variability.

### Changes in cell type abundance between experimental groups

To identify cell types that change in abundance between young and aged individuals (Extended Data Fig. 7a), we considered the following: first, different amounts of CD34^+^, CD3^+^ and total BM cells were sorted. Hence, frequencies were always computed within the respective gate (for example, for CD8^+^ effector T cells, the frequency among CD3^+^ T cells was computed). We then compared the following statistical models of observed cell type frequency _*pi*_ in individual *i*:

$$M_0:p_i\sim {\mathrm{Binom}}\left( q \right)\;{{{\mathrm{with}}}}\;q\sim {\mathrm{Beta}}\left( {1,1} \right)$$

$$M_1:p_i\sim {\mathrm{Binom}}\left( {q_{C\left( i \right)}} \right)\;{{{\mathrm{with}}}}\;q_{C\left( i \right)}\sim {\mathrm{Beta}}\left( {1,1} \right)$$

Here *C*(*i*) indicates if individual *i* is young or old.

Finally, we sought to distinguish between a model where cell type frequencies change as a function of age, and a model where cell type frequencies are simply highly variable between individuals, with no relationship to age:

$$M_2:p_i\sim {\mathrm{Binom}}\left( {q_i} \right)\;{{{\mathrm{with}}}}\;q_i\sim {\mathrm{Beta}}\left( {1,1} \right)$$

We compared the M1 and M2 models to M0 using a Bayesian strategy termed leave-one-out information criterion^63^ to identify cell types with high evidence for between-group and interindividual variability, respectively.

### Thresholding of surface marker expression

For every sample separately, thresholds were calculated using the normalized antibody counts to distinguish marker-positive from marker-negative cells. For this we implemented the Otsu algorithm as described by Otsu^64^.

### Data-driven identification of gating schemes

To account for the CD34^+^ FACS enrichment of HSPCs performed in our samples, we divided the BM cells into CD34^+^ and CD34^−^ subsets. For individual cell type gating scheme calculation, we compared three different methods. The first two methods are based on a decision tree using either the continuous normalized surface marker expression matrix named ‘Tree continuous’, or a transformed Boolean matrix (‘Tree Otsu’). For the latter method, a cutoff for each antibody was calculated using the histogram-based Otsu algorithm as described above and the matrix was binarized accordingly. In both cases, the tree was determined using the package Rpart and, if needed, pruned to the maximum number of required surface markers. The third method is based on the Hypergate algorithm^34^. For this, we used the target population as the Hypergate gate vector input, calculated the predicted gating scheme and calculated the channel contributions of each surface marker using a beta of 1. Afterwards we used the contributions to optimize the predicted gating scheme to only include the maximum number of surface markers selected. Furthermore, we gated the samples using a canonical gating scheme (Expert) reported in the literature to predict gatings for some of the cell types present in the BM (Supplementary Table 5). For this, we used the Otsu threshold to split each population into marker-positive and negative populations. For each gate, the following metrics were calculated: first, the purity (Pr), that is, the proportion of target cells in the final gate and second, the recall (Rc), that is, the proportion of target cells gated from their original total population.

For the simultaneous gating calculation of all cells from the HSC and progenitor compartment, we selected the cells from BM (Young1) with a CD34 surface expression higher than 0.95. Subsequently, we downsampled cells to the same number of cells across populations. Subsequently, we calculated the decision tree with the Rpart package, using the ‘continuous’ approach defined above.

### The NRN algorithm for integrating FACS and single-cell genomics data

To project flow cytometric measurements of surface protein abundance from CD34^+^ cells onto the single-cell reference, we initially subset the single-cell reference to exclude CD34^−^ cells, and flow cytometry data was transformed using the “logicle” transform using FlowJo (v.10.7.1). Subsequently, the expression of each surface marker was normalized separately both in the flow cytometry and in the Abseq dataset using a rank-based approach. In particular, sample ranks were computed and divided by the total number of samples, that is, data was mapped to a scale from 0 to 1 where 0 indicates lowest expression within the dataset, and 1 indicates highest expression. Within this normalized gene expression space, the cosine distance between any cell from the Abseq (reference) dataset and the FACS (query) dataset was computed, the four nearest reference neighbors of every query cell were identified and the average position of these neighbors in UMAP and pseudotime space was computed using scmap^65^. Subsequently, the average Euclidean distance of the reference neighbors in MOFA space was computed to identify cells with inconsistent mapping results. These cells were later removed by applying a user-defined threshold (here, 8). In the case of the Smart-seq2 dataset, a total of 75 cells were thereby removed from the analyses.

### Data visualization for a definition of boxplot elements

All plots were generated using the ggplot2 (v.3.2.1) package in R 3.6.2, GraphPad Prism (v.8 and v.9.1 for MacOS) or FlowJo (v.10.7.1, BD). Boxplots are defined as follows: the middle line corresponds to the median; the lower and upper hinges correspond to first and third quartiles, respectively; the upper whisker extends from the hinge to the largest value no further than 1.5× the interquartile range (or the distance between the first and third quartiles) from the hinge and the lower whisker extends from the hinge to the smallest value at most 1.5× the interquartile range of the hinge. Data beyond the end of the whiskers are called ‘outlying’ points and are plotted individually.

### Reporting Summary

Further information on research design is available in the Nature Research Reporting Summary linked to this article.

## Online content

Any methods, additional references, Nature Research reporting summaries, source data, extended data, supplementary information, acknowledgements, peer review information; details of author contributions and competing interests; and statements of data and code availability are available at 10.1038/s41590-021-01059-0.

## Supplementary information

Supplementary Information Supplementary Notes 1–8 and corresponding Figs. 1–8.

Reporting Summary

Supplementary Tables Supplementary Tables 1–7.

Supplementary Data 1 Overview of antibodies and fraction of variance explained by different biological processes.

Supplementary Data 2 Differential expression of surface markers in ageing and disease.

Supplementary Video 1 Short 4-min introduction that explains the usage of the Abseq App containing the resources of the paper.

Peer Review Information

## Data Availability

Data is available for interactive browsing at https://abseqapp.shiny.embl.de. Datasets including raw and integrated gene expression data, cell type annotation, metadata and dimensionality reduction are available as Seurat v.3 objects through figshare: https://figshare.com/projects/Single-cell_proteo-genomic_reference_maps_of_the_human_hematopoietic_system/94469. FACS data are provided through figshare: https://figshare.com/projects/Supplementary_data_FACS_data_from_Single-cell_proteo-genomic_reference_maps_of_the_human_hematopoietic_system/122716. Fastq files are available from the European Genome-Phenome Archive under accession number EGAS00001005593. Source data are provided with this paper.

## Source data

Source Data Fig. 3 Source data of Fig. 3f,j,n.
Source Data Fig. 4 Sample sizes for the boxplots in panel c and h, and links to remaining source data for the figure.
Source Data Fig. 5 Source data of Fig. 5e,f,j,k.
Source Data Extended Data Fig. 6 Source data of Extended Data Fig. 6c,g,h.
Source Data Extended Data Fig. 7 Sample sizes for the boxplots in panel f, and links to remaining source data for the figure.
Source Data Extended Data Fig. 8 Source data of Extended Data Fig. 8e,f.

## Code availability

The implementation of the NRN algorithm and vignettes describing the workflow for projecting single-cell RNA-seq data on the reference are available at https://git.embl.de/triana/nrn.
