# Supplementary material for: Single-cell proteo-genomic reference maps of the hematopoietic system enable the purification and massive profiling of precisely defined cell states
Source: Nat Immunol. 2021 Nov 22;22(12):1577–89. doi: 10.1038/s41590-021-01059-0 (PMC8642243; doi:10.1038/s41590-021-01059-0)
Supplement: Supplementary file 2 — Reporting Summary [file 41590_2021_1059_MOESM2_ESM.pdf]

nature research

Corresponding author(s): Lars Velten
Simon Haas

Last updated by author(s): Sep 13, 2021

Reporting Summary

Nature Research wishes to improve the reproducibility of the work that we publish. This form provides structure for consistency and transparency in reporting. For further information on Nature Research policies, see our Editorial Policies and the Editorial Policy Checklist.

Statistics

For all statistical analyses, confirm that the following items are present in the figure legend, table legend, main text, or Methods section.

n/a	Confirmed	
☐	☒	The exact sample size (*n*) for each experimental group/condition, given as a discrete number and unit of measurement
☐	☒	A statement on whether measurements were taken from distinct samples or whether the same sample was measured repeatedly
☐	☒	The statistical test(s) used AND whether they are one- or two-sided<br>*Only common tests should be described solely by name; describe more complex techniques in the Methods section.*
☐	☒	A description of all covariates tested
☐	☒	A description of any assumptions or corrections, such as tests of normality and adjustment for multiple comparisons
☐	☒	A full description of the statistical parameters including central tendency (e.g. means) or other basic estimates (e.g. regression coefficient) AND variation (e.g. standard deviation) or associated estimates of uncertainty (e.g. confidence intervals)
☐	☒	For null hypothesis testing, the test statistic (e.g. *F*, *t*, *r*) with confidence intervals, effect sizes, degrees of freedom and *P* value noted<br>*Give P values as exact values whenever suitable.*
☐	☒	For Bayesian analysis, information on the choice of priors and Markov chain Monte Carlo settings
☐	☒	For hierarchical and complex designs, identification of the appropriate level for tests and full reporting of outcomes
☐	☒	Estimates of effect sizes (e.g. Cohen's *d*, Pearson's *r*), indicating how they were calculated

Our web collection on statistics for biologists contains articles on many of the points above.

Software and code

Policy information about availability of computer code

Data collection

Raw data was processed using the standard Rhapsody analysis pipeline (BD Biosciences) on Seven Bridges (https://www.sevenbridges.com) according to the manufacturer's recommendations. Described in detail in the Methods, section "Seven Bridges processing for Abseq data".
Smart-seq2 data was processed using Kallisto, see Methods section, "Processing of Smart-seq2 data"
For the acquisition of flow cytometry data, BD FACSDiva was used. For the acqusition of qPCR data, a ViiA7 System (Applied Biosystems) was used.

Data analysis

Data were analyzed using R v.3.6.2 , Seurat v. 3.1.3 and v.3.2.0, MOFA v.1.3.1, caret v. 6.0-84, zinbwave v.1.8.0, scater 1.14.6, slingshot v.1.4.0, variancePartition v1.16.1, DESeq2 v1.26.0, randomForest_4.6-14, rpart_4.1-15, BIOMOD 1.1-7.04, Hypergate 0.8.3, scmap 1.8.0, Tradeseq 1.6.0 , ggplot2 v3.2.1 and rstatix 0.7.0.999. Python package scanorama v.16. A full description of data analysis is contained in the Methods, all sections starting with "Data analysis of Abseq data". Custom code is available at https://git.embl.de/triana/nrn
A custom web app was built additionally using packages shiny 1.6.0 and aws.S3 0.3.21 and was also used for data analysis (https://abseqapp.shiny.embl.de/)
Flow cytometry data was analyzed using FlowJo v.10.7.1.
ViiA7 System and corresponding software (v1.6.1) was used to analyze RT-qPCR data.
In some cases, GraphPad Prism v8 and v9.1 was used for graphical representations and statistics.
Microsoft Excel for mac v 16.16.27 or R Studio Server 1.2.5033 were used for data analysis in some cases.

For manuscripts utilizing custom algorithms or software that are central to the research but not yet described in published literature, software must be made available to editors and reviewers. We strongly encourage code deposition in a community repository (e.g. GitHub). See the Nature Research guidelines for submitting code & software for further information.

Data

Policy information about availability of data

All manuscripts must include a data availability statement. This statement should provide the following information, where applicable:

- Accession codes, unique identifiers, or web links for publicly available datasets
- A list of figures that have associated raw data
- A description of any restrictions on data availability

Data is available for interactive browsing at https://abseqapp.shiny.embl.de . Datasets including raw and integrated gene expression data, cell type annotation, metadata and dimensionality reduction are available as Seurat v3 objects through figshare: https://figshare.com/projects/Single-cell_proteo-genomic_reference_maps_of_the_human_hematopoietic_system/94469
Relevant flow cytometry and cell sorting FCS files are available through figshare:https://figshare.com/projects/Supplementary_data_FACS_data_from_Single-cell_proteo-genomic_reference_maps_of_the_human_hematopoietic_system/122716. Raw data from analyzed single cell index cultures are available upon request. Fastq files are available under accession number EGAS00001005593

Field-specific reporting

Please select the one below that is the best fit for your research. If you are not sure, read the appropriate sections before making your selection.

☒ Life sciences ☐ Behavioural & social sciences ☐ Ecological, evolutionary & environmental sciences

For a reference copy of the document with all sections, see nature.com/documents/nr-reporting-summary-flat.pdf

Life sciences study design

All studies must disclose on these points even when the disclosure is negative.

Sample size	No statistical methods were used to pre-determine sample size, but an approximate calculation based on experience suggested that typically, 50 cells are enough to robustly define a cell type and hence 60,000 CD34- and 11,000 CD34+ cells in the main reference dataset would be sufficient to identify populations present at <0.1% of total or <0.5% of CD34+ BM, respectively. In practice, obtained sample size was also affected by the fraction of high quality cells that passed quality metrics and in the case of CD34+ cells the availability of biological material. In practice, the smallest population we identified in the main dataset was the Mesenchymal cell 2 population, which was covered with 11 cells in the final dataset (0.02% of total BM); the smallest CD34+ hematopoietic population were the putative NK cell progenitors, covered with 30 cells (0.2% of CD34+). Hence in practice given our dataset size (n=70017 cells), very small populations can robustly be identified.
Data exclusions	a) Abseq: For targeted scRNA-seq no filtering was performed for the WTA sample cells with < 500 detected genes and >30% mitochondrial counts were discarded. Such quality control steps are customary in the field and were set after inspection of the data, and not pre-established.<br>b) indexed Smart-seq2: All cells with mitochondrial reads > 20% were excluded. Second, we limited the acceptable numbers of detected genes. Cells with < 1000 detected genes were discarded. Such quality control steps are customary in the field and were pre-established.<br>c) single-cell index culture readouts: generally, only wells that contained more than 10 CD45+ CD235- or CD45+ CD235+ or CD45+ CD235- cells were considered during analysis.
Replication	3 independent bone marrow donors were assayed per experimental group (healthy young, healthy aged, AML). All findings on healthy young and healthy old bone marrow donors were successfully replicated. 12 more individual AML/APL patients were used to make in depth statements about disease states. Sample metadata can be found in Supplementary Table 3.<br>For RT-qPCR sorts, cells from at least three individual human BM donors were sorted individually and gene expression was analyzed in technical triplicates. RT-qPCR experiments were only performed once, due to sample availability. For FACS based index sorting, both index single-cell cultures and index RNA-seq was performed with cells from the same donor. A second single-cell index culture experiment (Figure S9 e,f) succesfully replicated data shown in Figure 3 (Figure 3 f,j,n).
Randomization	Not relevant (no treatment groups)
Blinding	Blinding during single cell data generation was not performed as the same standard Abseq protocol and library preparation protocol was followed for each of the bone marrow samples. FACS analysis required grouping of data and sample identities could not be blinded.

Reporting for specific materials, systems and methods

We require information from authors about some types of materials, experimental systems and methods used in many studies. Here, indicate whether each material, system or method listed is relevant to your study. If you are not sure if a list item applies to your research, read the appropriate section before selecting a response.

Materials & experimental systems

n/a	Involved in the study
☐	☒ Antibodies
☒	☐ Eukaryotic cell lines
☒	☐ Palaeontology and archaeology
☒	☐ Animals and other organisms
☐	☒ Human research participants
☒	☐ Clinical data
☒	☐ Dual use research of concern

Methods

n/a	Involved in the study
☒	☐ ChIP-seq
☐	☒ Flow cytometry
☒	☐ MRI-based neuroimaging

Antibodies

Antibodies used

For a list of oligo coupled antibodies, see Supplementary Table S2

FACS antibodies (Epitope+Fluorochrome, Vendor + Catalog number, RRID, clone, dilution):

ABseq cell sorting
Anti-CD34 PE BD Biosciences Cat# 555822, RRID:AB_396151 ,1:30
DAPI BD Biosciences Cat# 564907, RRID:AB_2869624 ,1:1000
Caspase 3/7 probe Thermo Fisher Scientific Cat# C10423 ,1:500
Anti-CD3 PE-Cy7 BD Biosciences Cat# 560910, RRID:AB_10563409 ,SK7 ,1:50
Anti-CD45RA BB515 BD Biosciences Cat# 564552, RRID:AB_2738841 ,HI100 ,1:50
Anti-CD38 APC-R700 BD Biosciences Cat# 564979, RRID:AB_2744373 ,HIT2 ,1:200
Anti-CD10 BV421 BioLegend Cat# 312218, RRID:AB_2561833 ,HI10a ,1:50
Fixable viability dye efluor506 Thermo Fisher Scientific Cat# 65-0866-14 ,1:1000

Classification panel
Anti-CD4 BV421 BD Biosciences Cat# 565997, RRID:AB_2739448 ,SK3 ,1:20
Anti-CD11a BUV395 BD Biosciences Cat# 745986, RRID:AB_2743392 ,HI111 ,1:100
Anti-CD33 BV785 BD Biosciences Cat# 740974, RRID:AB_2740599 ,WM53 ,1:50
Anti-CD123 BV650 BD Biosciences Cat# 563405, RRID:AB_2738185 ,7G3 ,1:50
Anti-CD19 BB700 BD Biosciences Cat# 566396, RRID:AB_2744310 ,SJ25C1 ,1:50
Anti-CD61 BB515 BD Biosciences Cat# 565123, RRID:AB_2739075 ,VI-PL2 ,1:50
Anti-CD10 APC BioLegend Cat# 312209, RRID:AB_314920 ,HI10a ,1:50
Anti-CD38 APC-R700 BD Biosciences Cat# 564979, RRID:AB_2744373 ,HIT2 ,1:200
Anti-CD34 APC-Cy7 BioLegend Cat# 343514, RRID:AB_1877168 ,581 ,1:30
Anti-CD133 PE BD Biosciences Cat# 566593, RRID:AB_2744281 ,W6B3C1 ,1:30
Anti-CD11c PE-Cy7 BD Biosciences Cat# 561356, RRID:AB_10611859 ,B-ly6 ,1:50
Anti-Tim-3 PE-CF594 BD Biosciences Cat# 565560, RRID:AB_2744371 ,7D3 ,1:30
Fixable viability dye efluor506 Thermo Fisher Scientific Cat# 65-0866-14 ,1:500

Semi-automated panel
Anti-CD49b BV421 BD Biosciences Cat# 743201, RRID:AB_2871492 ,12F1 ,1:50
Anti-CD326 PE-CF594 BD Biosciences Cat# 565399, RRID:AB_2739219 ,EBA-1 ,1:50
Anti-CD71 APC BioLegend Cat# 334107, RRID:AB_10916388 ,CY1G4 ,1:200
Anti- FcER1A PE-Cy7 Thermo Fisher Scientific Cat# 25-5899-42, RRID:AB_2573495 ,AER-37 ,1:50
Anti-CD11a BUV395 BD Biosciences Cat# 745986, RRID:AB_2743392 ,HI111 ,1:100
Anti-CD33 BV785 BD Biosciences Cat# 740974, RRID:AB_2740599 ,WM53 ,1:50
Anti-CD123 BV650 BD Biosciences Cat# 563405, RRID:AB_2738185 ,7G3 ,1:50
Anti-CD61 BB515 BD Biosciences Cat# 565123, RRID:AB_2739075 ,VI-PL2 ,1:50
Anti-CD38 APC-R700 BD Biosciences Cat# 564979, RRID:AB_2744373 ,HIT2 ,1:200
Anti-CD34 APC-Cy7 BioLegend Cat# 343514, RRID:AB_1877168 ,581 ,1:30
Anti-CD133 PE BD Biosciences Cat# 566593, RRID:AB_2744281 ,W6B3C1 ,1:30
Fixable viability dye efluor506 Thermo Fisher Scientific Cat# 65-0866-14 ,1:1000

Stem and progenitor panel
Anti-CD4 BV421 BD Biosciences Cat# 565997, RRID:AB_2739448 ,SK3 ,1:20
Anti-CD45RA BB515 BD Biosciences Cat# 564552, RRID:AB_2738841 ,HI100 ,1:50
Anti-CD98 BB700 BD Biosciences Cat# 746147, RRID:AB_2743507 ,UM7F8 ,1:50
Anti-CD90 PE BD Biosciences Cat# 555596, RRID:AB_395970 ,5E10 ,1:30
Anti-CD49f PE-Cy7 Thermo Fisher Scientific Cat# 12-0495-82, RRID:AB_891474 ,eBioGoH3 ,1:50
Anti-CD38 APC-R700 BD Biosciences Cat# 564979, RRID:AB_2744373 ,HIT2 ,1:200
Anti-CD34 APC-Cy7 BioLegend Cat# 343514, RRID:AB_1877168 ,581 ,1:30
Anti-CD133 BV650 BD Biosciences Cat# 747642, RRID:AB_2744206 ,W6B3C1 ,1:30
Fixable viability dye efluor506 Thermo Fisher Scientific Cat# 65-0866-14 ,1:1000
Anti-CD11a BUV395 BD Biosciences Cat# 745986, RRID:AB_2743392 ,HI111 ,1:100
Anti-Tim-3 PE-CF594 BD Biosciences Cat# 565560, RRID:AB_2744371 ,7D3 ,1:30
Anti-CD10 APC BioLegend Cat# 312209, RRID:AB_314920 ,HI10a ,1:50

sc-index culture readouts

Anti-CD303 BUV395 BD Biosciences Cat# 747999, RRID:AB_2872460 ,V24-785 ,1:200
Anti-CD14 BUV805 BD Biosciences Cat# 612902, RRID:AB_2870189 ,M5E2 ,1:150
Anti-CD141 BV421 BioLegend Cat# 344114, RRID:AB_2563858 ,M80 ,1:200
Anti-CD19 BV785 BioLegend Cat# 302240, RRID:AB_2563442 ,HIB19 ,1:200
Anti-CD56 BV785 BD Biosciences Cat# 564058, RRID:AB_2738569 ,NCAM16.2 ,1:200
Anti-CD41a FITC Thermo Fisher Scientific Cat# 11-0419-42, RRID:AB_10718234 ,HIP8 ,1:200
Anti-CD66b PerCP-Cy5.5 BioLegend Cat# 305107, RRID:AB_2077856 ,G10F5 ,1:200
Anti-CD370 PE BioLegend Cat# 353804, RRID:AB_10965546 ,8F9 ,1:200
Anti-CD1c PE-Dazzle 594 BioLegend Cat# 331532, RRID:AB_2565293 ,L161 ,1:200
Anti-CD235a APC Thermo Fisher Scientific Cat# 17-9987-42, RRID:AB_2043823 ,HIR2 ,1:200
Anti CD45 APC-R700 BD Biosciences Cat# 566041, RRID:AB_2744399 ,HI30 ,1:200
Anti-CD123 BV650 BD Biosciences Cat# 563405, RRID:AB_2738185 ,7G3 ,1:200
Fixable viability dye efluor506 Thermo Fisher Scientific Cat# 65-0866-14 ,1:1000
Anti-CD34 APC-Cy7 BioLegend Cat# 343514, RRID:AB_1877168 ,581 ,1:200
Anti- FcER1A PE-Cy7 Thermo Fisher Scientific Cat# 25-5899-42, RRID:AB_2573495 ,AER-37 ,1:200
Anti- CD11b BUV615 BD Biosciences Cat# 751140, RRID:AB_2875166 ,M1/70 ,1:200
Anti- CD1c APC-Cy7 BioLegend Cat# 331520, RRID:AB_10644008 ,L161 ,1:200
Anti- CD56 PE-Dazzle BioLegend Cat# 362544, RRID:AB_2565922 ,5.1H11 ,1:200

Cytotoxic CD4+ T cell analysis
Anti-CD3 BUV395 BD Biosciences Cat# 563546, RRID:AB_2744387 ,UCHT1 ,1:50
Anti-CD25 BUV737 D Biosciences Cat# 564385, RRID:AB_2744342 ,2A3 ,1:50
Anti-CD4 BUV805 BD Biosciences Cat# 612887, RRID:AB_2870176 ,SK3 ,1:50
Anti-CD197 Pacific Blue BioLegend Cat# 353210, RRID:AB_10918984 ,G043H7 ,1:50
Anti-CD7 BV711 BD Biosciences Cat# 564018, RRID:AB_2738544 ,M-T701 ,1:50
Anti-CD45RO FITC BioLegend Cat# 304242, RRID:AB_2564159 ,UCHL1 ,1:50
Anti-CD28 PE-CF594 BD Biosciences Cat# 562323, RRID:AB_11153681 ,CD28.2 ,1:50
Anti-CD127 PE-Cy7 Thermo Fisher Scientific Cat# 25-1278-42, RRID:AB_1659672 ,eBioRDR5 ,1:50
Anti-CD45RA APC Thermo Fisher Scientific Cat# 17-0458-41, RRID:AB_1944379 ,HI100 ,1:50
Anti-CD45 Alexa Fluor 700 BioLegend Cat# 304024, RRID:AB_493761 ,HI30 ,1:50
Anti-CD69 APC-Cy7 BD Biosciences Cat# 560912, RRID:AB_10563414 ,FN50 ,1:100
Fixable viability dye efluor506 Thermo Fisher Scientific Cat# 65-0866-14 ,1:1000

MSC analysis
Anti-CD10 BV421 BioLegend Cat# 312209, RRID:AB_314920 ,HI10a ,1:50
Anti-CD146 BV785 BD Biosciences Cat# 743303, RRID:AB_274141 ,P1H12 ,1:50
Anti-CD105 FITC BioLegend Cat# 323204, RRID:AB_755956 ,43A3 ,1:50
Anti-CD31 BB700 BD Biosciences Cat# 566563, RRID:AB_2744362 ,WM59 ,1:100
Anti-CD49a PE BD Biosciences Cat# 559596, RRID:AB_397288 ,SR84 ,1:20
Anti-CD13 PE-Dazzle 594 BioLegend Cat# 301719, RRID:AB_2616763 ,WM15 ,1:50
Anti-CD271 PEVio770 Miltenyi Biotec Cat# 130-113-422, RRID:AB_2733220 ,ME20.4-1.H4 ,1:100
Anti-CD26 APC BD Biosciences Cat# 563670, RRID:AB_2738363 ,M-A261 ,1:50
Anti-CD45 APC R700 BD Biosciences Cat# 566041, RRID:AB_2744399 ,HI30 ,1:100
Anti-CD90 APC-Cy7 BioLegend Cat# 328132, RRID:AB_2566341 ,5E10 ,1:100
Fixable viability dye efluor506 Thermo Fisher Scientific Cat# 65-0866-14 ,1:50
Anti-CD11a BUV395 BD Biosciences Cat# 745986, RRID:AB_2743392 ,HI111 ,1:100

Erythroid/Megakaryocyte differentiation panel
Anti-CD38 BUV563 BD Biosciences Cat# 741446, RRID:AB_2870920 ,HB7 ,1:200
Anti-CD71 BUV805 BD Biosciences Cat# 749294, RRID:AB_2873669 ,M-A712 ,1:200
Anti-CD49b BV421 BD Biosciences Cat# 743201, RRID:AB_2871492 ,12F1 ,1:50
Fixable viability dye efluor506 Thermo Fisher Scientific Cat# 65-0866-14 ,1:1000
Anti-CD44 BV650 BD Biosciences Cat# 743665, RRID:AB_2871540 ,L178 ,1:200
Anti-CD49d BV711 BD Biosciences Cat# 563177, RRID:AB_2738049 ,9F10 ,1:50
Anti-CD45RA BB515 BD Biosciences Cat# 564552, RRID:AB_2738841 ,HI100 ,1:100
Anti-CD90 PE BD Biosciences Cat# 555596, RRID:AB_395970 ,5E10 ,1:30
Anti-CD326 PE-CF594 BD Biosciences Cat# 565399, RRID:AB_2739219 ,EBA-1 ,1:50
Anti-CD123 PE-Cy7 Thermo Fisher Scientific Cat# 25-1239-42, RRID:AB_1257136 ,6H6 ,1:50
Anti-CD41 APC Thermo Fisher Scientific Cat# 17-0419-42, RRID:AB_2573144 ,HIP8 ,1:50
Anti-CD34 APC-Cy7 BioLegend Cat# 343514, RRID:AB_1877168 ,581 ,1:50

Consensus panel
Anti-CD11a BUV395 BD Biosciences Cat# 745986, RRID:AB_2743392 ,HI111 ,1:50
Anti-CD71 BUV805 BD Biosciences Cat# 749294, RRID:AB_2873669 ,M-A712 ,1:400
Anti-CD45RA BV421 BD Biosciences Cat# 562885, RRID:AB_2737864 ,HI100 ,1:50
Fixable viability dye efluor506 Thermo Fisher Scientific Cat# 65-0866-14 ,1:1000
Anti-CD44 BV650 BD Biosciences Cat# 743665, RRID:AB_2871540 ,L178 ,1:300
Anti-CD135 BV711 BD Biosciences Cat# 563908, RRID:AB_2738479 ,4G8 ,1:20
Anti-Tim3 FITC BioLegend Cat# 345021, RRID:AB_2563936 ,F38-2E2 ,1:30
Anti-CD90 PE BD Biosciences Cat# 555596, RRID:AB_395970 ,5E10 ,1:30
Anti-CD326 PE-CF594 BD Biosciences Cat# 565399, RRID:AB_2739219 ,EBA-1 ,1:50
Anti-CD41 PE-Cy5 BioLegend Cat# 303708, RRID:AB_314378 ,HIP8 ,1:50
Anti-CD123 PE-Cy7 Thermo Fisher Scientific Cat# 25-1239-42, RRID:AB_1257136 ,6H6 ,1:30
Anti-CD10 APC BioLegend Cat# 312209, RRID:AB_314920 ,HI10a ,1:30
Anti-CD38 APC-R700 BD Biosciences Cat# 564979, RRID:AB_2744373 ,HIT2 ,1:150
Anti-CD34 APC-Cy7 BioLegend Cat# 343514, RRID:AB_1877168 ,581 ,1:50

Validation	All antibodies used are commercially available, broadly established and validated by the respecitve manufacturers as indicated on the websites (See RRIDS above for respective websites for each antibody). In addition, used antibodies are used routinely in our laborarotory with reproducible results.

Human research participants

Policy information about studies involving human research participants

Population characteristics	The age of young healthy donors ranged between 25 and 34. Aged healthy donors ranged between 59 and 69 years. AML patients ranged between 44 and 78 years. APL patients ranged between 32 and 70 years. Both female and male BM donors were included in every group.<br>Healthy young and aged patients showed normal BM cellularity and were devoid of any known mutations.<br>AML/APL patient samples used in this study were taken at initial diagnosis. AML Patients had normal karyotypes. Following list states diseased patient metadata in detail:<br>AML1 FLT3-ITD,NPM1-mut<br>AML2 FLT3-wt,NPM1-mut<br>AML3 FLT3-ITD,NPM1-mut<br>AML Q4 FLT3-ITD,NPM1-mut<br>AML Q1 FLT3-ITD,NPM1-mut<br>AML Q3 FLT3-wt,NPM1-mut<br>AML Q6 FLT3-wt,NPM1-mut<br>AML Q2 FLT3-wt,NPM1-mut<br>AML Q5 FLT3-wt,NPM1-mut<br>APL Q5 APL t(15;17)<br>APL Q3 APL t(15;17)<br>APL Q6 APL t(15;17)<br>APL Q4 APL t(15;17)<br>APL Q2 APL t(15;17)<br>APL Q1 APL t(15;17)
Recruitment	Young healthy donors and aged healthy donor BM samples were obtained from patients that are without any clinical signs of disease. AML patient samples were obtained at initial diagnosis and had normal karyotypes. Rare samples of healthy young and aged donors were included with respect to sample availability.
Ethics oversight	Bone marrow (BM) samples from healthy and diseased donors were obtained at the University clinics in Heidelberg and Mannheim after informed written consent using ethic application numbers S480/2011 and S-693/2018. All experiments involving human samples were approved by the ethics committee of the University Hospital Heidelberg and was in accordance with the Declaration of Helsinki.

Note that full information on the approval of the study protocol must also be provided in the manuscript.

Flow Cytometry

Plots

Confirm that:

☒ The axis labels state the marker and fluorochrome used (e.g. CD4-FITC).

☒ The axis scales are clearly visible. Include numbers along axes only for bottom left plot of group (a 'group' is an analysis of identical markers).

☒ All plots are contour plots with outliers or pseudocolor plots.

☒ A numerical value for number of cells or percentage (with statistics) is provided.

Methodology

Sample preparation	After obtaining informed written consent, healthy or diseased bone marrow samples were collected from iliac crest bone marrow aspirations. Peripheral blood was obtained from venipuncture. Mononuclear cells from bone marrow and blood were isolated by density gradient centrifugation, frozen and stored in liquid nitrogen until further use.<br>For sample preparation, samples were thawed in a water bath at 37°C and transferred dropwise into RPMI-1640 10% FCS. Cells were centrifuged for 5 min at 350g and washed once with RPMI-1640 10% FCS. Cells were then resuspended in FACS buffer (FB, PBS 5% FCS 0.5 mM EDTA) containing fluorochrome conjugated antibodies, dead cell exclusion dye and Fc-receptor blocking solution. Cell suspensions were incubated for 15 min at 4°C in the dark. Cell suspensions were then washed with FB and resuspended in 0,2- 1 ml FB and were inserted into the respective analyzer or cell sorter.
Instrument	All flow cytometric analyses were performed using BD Fortessa or LSRII flow cytometers. Cell sorting was done using BD Aria Aria II and BD Aria Fusion sorters equipped with 100 µm or 130 µm nozzle and sorting was performed in 4-way purity or single cell purity modes.

Software	BD FACSDiva and FlowJo v10.7.1 were used throughout the study. In some cases, logicle transformed FCS data using a built-in FlowJo function was exported and plotted in R using the ggplot2 v3.2.1. package.
Cell population abundance	Purity in post sort fractions was not directly determined. Post-sort, single-cell RNA seq and RT-qPCRs were performed, which gave detailed insights into the biology of sorted cell populations.
Gating strategy	FSC-SSC gates were set so that FSC low and SSC high cells were excluded, following by singlet gating using FSC-A vs. FSC-H. After doublet exlcusion, dead cells, which are efluor506 high were excluded according to the manufacters's instructions and the indicated gating strategies (see respective figures that show flow cytometry data, i.e Fig. 5 and Fig.6) were followed. During Abseq cell sorts, a combination of Caspase 3/7 and DAPI was used for dead cell exclusion and is shown in Supplemental Figure 2.

☒ Tick this box to confirm that a figure exemplifying the gating strategy is provided in the Supplementary Information.
