# Supplementary material for: Single-cell proteo-genomic reference maps of the hematopoietic system enable the purification and massive profiling of precisely defined cell states
Source: Nat Immunol. 2021 Nov 22;22(12):1577–89. doi: 10.1038/s41590-021-01059-0 (PMC8642243; doi:10.1038/s41590-021-01059-0)
Supplement: Supplementary file 7 — Peer Review Information [file 41590_2021_1059_MOESM7_ESM.pdf]

Peer Review Information

Journal: Nature Immunology
Manuscript Title: Single-cell proteo-genomic reference maps of the hematopoietic system enable the purification and massive profiling of precisely defined cell states
Corresponding author name(s): Lars Velten and Simon Haas

Editorial Notes:

**Redactions – transferred manuscripts (mention of the other journal)**	This manuscript has been previously reviewed at another journal. This document only contains reviewer comments, rebuttal and decision letters for versions considered at Nature Immunology. Mentions of the other journal have been redacted.

Reviewer Comments & Decisions:

Decision Letter, initial version:

Subject: Decision on Nature Immunology submission NI-RS31634-T
Message: 26th Feb 2021

Dear Simon and Lars,

Thank you for transferring your manuscript from [REDACTED] and our earlier chat about the data. I've now discussed this with my colleagues and we'd be willing to consider the manuscript further. Please prepare an experimental revision taking into account all the issues of the three Referees. If you feel some points are beyond the scope please include discussion addressing this. Bear in mind the primarily immunological audience and that the technical advancements are perhaps secondary to the utility of the dataset.

If you choose to revise your manuscript taking into account all reviewer and editor comments, please highlight all changes in the manuscript text file .

We are committed to providing a fair and constructive peer-review process. Do not hesitate to contact us if there are specific requests from the reviewers that you believe are technically impossible or unlikely to yield a meaningful outcome.

If revising your manuscript:

* Include a “Response to referees” document detailing, point-by-point, how you addressed each referee comment. If no action was taken to address a point, you must provide a compelling argument. This response will be sent back to the referees along with the revised manuscript.

* If you have not done so already please begin to revise your manuscript so that it conforms to our Resource format instructions at http://www.nature.com/ni/authors/index.html. Refer also to any guidelines provided in this letter.

* Include a revised version of any required reporting checklist. It will be available to referees (and, potentially, statisticians) to aid in their evaluation if the manuscript goes back for peer review. A revised checklist is essential for re-review of the paper.

The Reporting Summary can be found here:
https://www.nature.com/documents/nr-reporting-summary.pdf

When submitting the revised version of your manuscript, please pay close attention to our href="https://www.nature.com/nature-research/editorial-policies/image-integrity">Digital Image Integrity Guidelines. and to the following points below:

-- that unprocessed scans are clearly labelled and match the gels and western blots presented in figures.
-- that control panels for gels and western blots are appropriately described as loading on sample processing controls
-- all images in the paper are checked for duplication of panels and for splicing of gel lanes.

Finally, please ensure that you retain unprocessed data and metadata files after publication, ideally archiving data in perpetuity, as these may be requested during the peer review and production process or after publication if any issues arise.

You may use the link below to submit your revised manuscript and related files:
[REDACTED]

Note: This URL links to your confidential home page and associated information about manuscripts you may have submitted, or that you are reviewing for us. If you wish to forward this email to co-authors, please delete the link to your homepage.

If you wish to submit a suitably revised manuscript we would hope to receive it within 6 months. If you cannot send it within this time, please let us know. We will be happy to consider your revision so long as nothing similar has been accepted for publication at Nature Immunology or published elsewhere.

Nature Immunology is committed to improving transparency in authorship. As part of our efforts in this direction, we are now requesting that all authors identified as ‘corresponding author’ on published papers create and link their Open Researcher and Contributor Identifier (ORCID) with their account on the Manuscript Tracking System (MTS), prior to

acceptance. ORCID helps the scientific community achieve unambiguous attribution of all scholarly contributions. You can create and link your ORCID from the home page of the MTS by clicking on 'Modify my Springer Nature account'. For more information please visit please visit www.springernature.com/orcid.

Please do not hesitate to contact me if you have any questions or would like to discuss the required revisions further.

Thank you for the opportunity to review your work.

Sincerely,

Zoltan Fehervari, Ph.D.
Senior Editor
Nature Immunology

The Macmillan Building
4 Crinan Street
Tel: 212-726-9207
Fax: 212-696-9752
z.fehervari@nature.com

Author Rebuttal to Initial comments

Point-by-point response *Triana, Vonficht, Jopp-Saile et al*

Reviewer #1

Remarks to the Author:

The manuscript by Triana et al. uses coupled highly multiplexed cell surface labelling and transcriptome analysis to generate a correlative map between transcriptional and immunophenotypic cell states in normal, aged and pathological hematopoiesis. They use these data to identify new marker to isolate functionally relevant T-cell and MSC subsets. Such a resource, in conjunction with the analytical tools generated, could be valuable to the field. However, some issues should be addressed:

1. The authors quite rightly state that there is debate regarding the presence of multipotent cell types as intermediaries in hematopoietic lineage specification. However, they fail to cite key publications that provide support of this notion (Belluschi 2018, PMID: 30291229; Karamitros 2019, PMID: 29167569). One of the cited papers (Drissen, 2019) also argues in favour of this. Generally, there seems to be a correlation between the quality of the functional progenitor analysis and the ability to detect such cells: in studies where such oligopotent cells were not readily detected (Notta, 2016; Velten, 2017) readouts were less detailed and comprehensive. A more inclusive presentation of this issue should therefore be considered. Also, the term "HSC

commitment" seems to be used to represent the entire process of differentiation – it would normally be understood to involve the discrete step where HSCs commit to a non-self-renewing state.

We thank this reviewer for the valuable comments. We have now included a more balanced discussion on the debate regarding the presence of multipotent cell types as intermediaries in hematopoietic lineage specification. We would like to emphasize that in our understanding the manuscripts of Velten et al., 2017 and Notta et al., 2016 do not argue against the existence of oligopontent intermediaries, but rather suggested that in adult BM they are less abundant than expected by the classical model and that only a subset of cell types in immunophenotypically defined oligopotent FACS gates behave functionally oligopotent. We agree with the reviewer's comment that the actual culture conditions might also impact on the readout of potency. In our study, we have used state-of-the art culture conditions containing a cocktail of 12 growth factors and cytokines, and feeder cells to support cell growth of several lineages, including erythroid, megakaryocyte, monocyte, neutrophil, eosinophil/basophil, dendritic cell, and, to a lesser degree, lymphoid lineages. In the initial version of the manuscript, we had observed that phenotypically immature cells (HSCs/MPPs) give rise to many different combinations of cell types, whereas phenotypically more mature cells (classical MEPs/GMPs, etc.) give predominantly rise to single lineages. To address the point of the reviewer, we have now performed a detailed statistical analysis of the combinations of cell types produced from single cells in the culture data. This analysis revealed that combinations of Ery/Mk/Eo/Baso as well as combinations of Lympho/DC/Neutro/Mono fates are more frequently realized together than expected under a null model where fates are stochastically realized independently from each other (new Figure 7e, f, and Response Figure 1a-b). These results are in line with the transcriptomic cell state trajectories, where we observe two primary branches corresponding to Ery/Mk/Eo/Baso versus Lympho/DC/Neutro/Mono lineages, which subsequently sub-segregate into the individual lineages. However, phenotypically highly similar HPSCs frequently give rise to heterogeneous functional outputs and cells reminiscent of highly immature or early primed HSPCs often show unipotent functional outputs (new Figure 7g, and Response Figure 1c). These findings suggest that oligopotency might be a highly transitory state and that stochastic processes might play a role in determining the functional output of a cell with a defined transcriptome, or they hint towards layers of cell fate regulation not observed in the transcriptome. Ordering of HSPCs according to their phenotypically approximated pseudotime confirmed the highly transient nature of functional oligo / multipotency (added to Figure 7d, see Response Figure 1d). In summary, our data is both in line with most recent findings on routes of lineage-commitment (Drissen et al. 2019, Belluschi et al. 2018, Goergens et al. 2015), as well as with manuscripts demonstrating that oligopotent cell states are less abundant than expected by the classical model (Notta et al. 2016, Velten et al. 2017, Paul et al. 2015), and might help reconciling discrepancies in the interpretation of previous studies. The new analyses and points have been incorporated into the revised version of the manuscript (line number 396-409)

We also thank the reviewer for the comment regarding the specific definition of the term 'HSC lineage commitment', which we have now consistently replaced by 'lineage commitment' to avoid confusion.

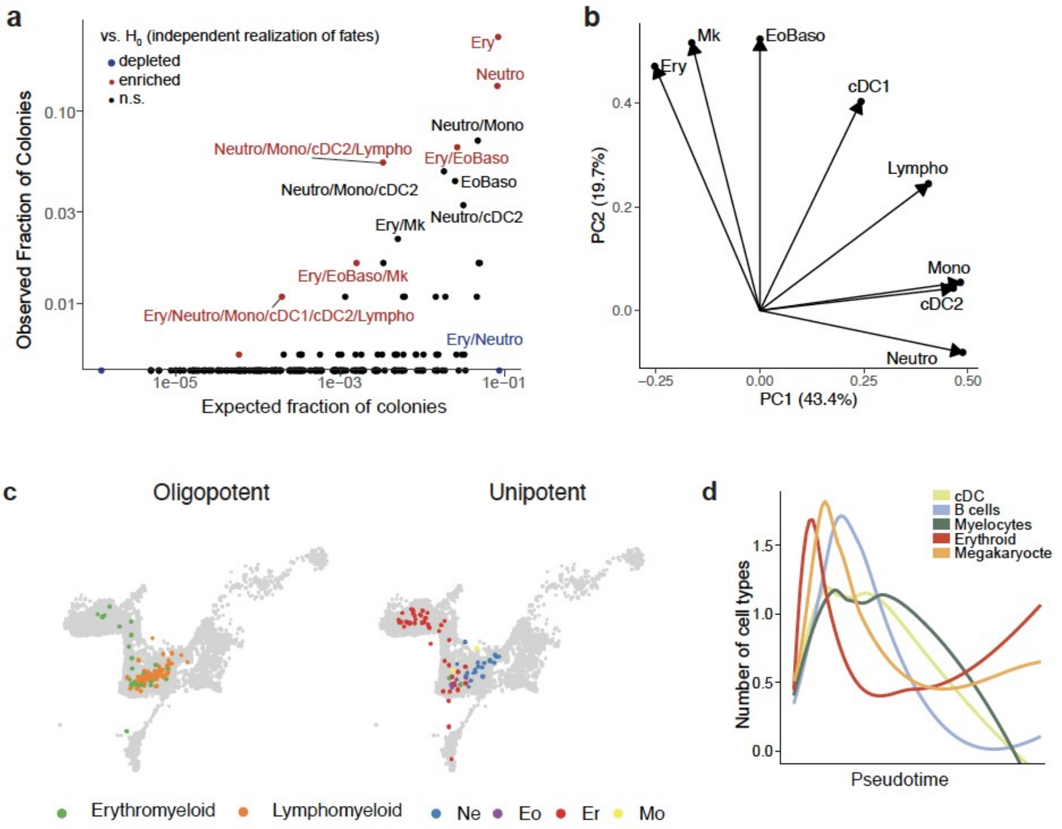

***Response Figure 1, corresponding to new Figures 7e-g, d**: **a.** Analysis of cell type combinations. For any combination of Erythroid (Ery), Neutrophil (Neutro), Monocytic (Mono), Eosinophil or Basophil (EoBaso), Lymphoid (Lympho), Megakaryocytic (Mk) and Dendritic (cDC1 and cDC2) potential, the scatter plot depicts the fraction of colonies containing this exact combination of cell types (y-axis) and the theoretical fraction of colonies containing this exact combination of cell types under the assumption that cell fates are independently realized with the same marginal probabilities (x-axis). Significance was calculated from a binomial test and is color-coded. **b.** PCA analysis of colony compositions. **c.** Distribution of colonies with frequent combinations of cell types in the projected UMAP space. Erythromyeloid: Only containing Eo/Baso (Eo), Mk and/or Ery (Er) cells; Lymphomyeloid: All other combinations; other abbreviations: Mo = Monocytic, Ne = Neutrophil. **d.** The total number of cell types per colony are highlighted both on the on projected pseudotime, see main Figure 7 legend for details.*

2. Along the same lines, when evaluating whether the platform generated provides an advance over the state-of-the-art (Figure 6) using the Doulatov gating scheme as the baseline

does not provide an accurate picture. This is not the prevailing definition of the populations mentioned, and as noted, several papers have improved on this classification (see point 1, also Psaila 2016, PMID: 27142433). It would be appropriate to use the state-of-the-art as the baseline, rather than a reference point known to be outdated, if added value is to be demonstrated.

We do agree with this reviewer that a more comprehensive benchmarking of the performance of our gating scheme is of importance. We would like to emphasize that the main goal of our approach was to identify a mathematically optimal FACS gating scheme that would describe the full transcriptomic complexity of the entire HSPC compartment as adequately as possible with a restricted number of surface markers (Figure 6c and see below). Therefore, we initially compared this approach against other global FACS gating schemes that describe the entire HSPC compartment (Doulatov et al., 2010, Figure 6d and Response Figure 2a) and not to specialized schemes that describe sub-branches of the hematopoietic hierarchy. We have now compared our global gating to an extended global gating scheme focusing on lympho-myeloid differentiation (Karamitros et al., 2018, new Figure S9a and Response Figure 2b). In order to create a 'Consensus' representative for state-of-the-art gating schemes in the field, we combined the scheme from Doulatov et al., Karamitros et al. in silico with the gating from Psaila et al. 2016, focusing on erythroid/megakaryocytic differentiation (new Figure S9b and Response Figure 2c). The performance of our data-defined gating scheme outperforms all of these expert-defined schemes (updated Figure 6e and Response Figure 2d,e). The new analysis is included in the main text (line 341-346). Importantly, our data resource and online platform constitute a powerful framework for the community to create individualized highly precise, specialized gating schemes that complement the global gating scheme introduced here.

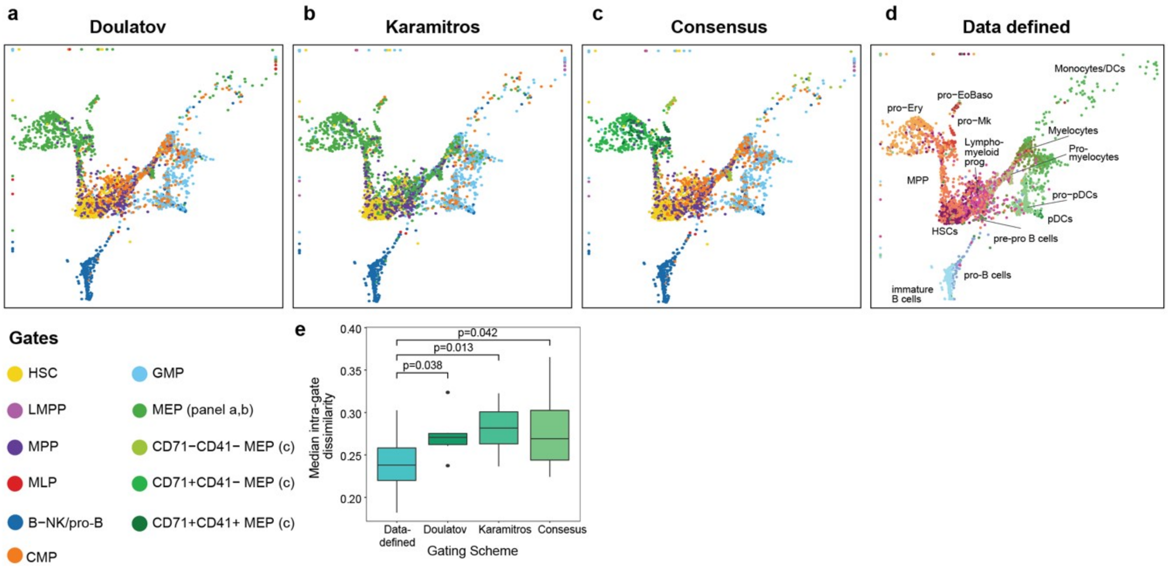

Response Figure 2: Comparison of different gating schemes. Corresponds to main Figure 6c-e and Supplementary Figure 9a, b. a. *UMAP highlighting classification obtained from the gating scheme described by from Doulatov et al., 2010, i.e. HSC: CD34+CD38-CD45RA-CD90+; MPP: CD34+CD38-CD45RA-CD90-; MLP: CD34+CD38-CD45RA+; CMP: CD34+CD38+CD10-CD45RA-Flt3+; MEP: CD34+CD38+CD10-CD45RA-Flt3-; GMP: CD34+CD38+CD10-CD45RA+Flt3+; pro-B: CD34+CD38+CD10+.* ***b.*** *UMAP highlighting classification obtained from the gating scheme described by Karamitros et al., 2018, i.e. HSC: CD34+CD38-CD10-CD45RA-CD90+; MPP: CD34+CD38-CD10-CD45RA-CD90-; LMPP:CD34+CD38-CD10-CD45RA+; MLP: CD34+CD38-CD10+; MEP: CD34+CD38+CD10-CD45RA-CD123-; CMP: CD34+CD38+CD10-CD45RA-CD123+; GMP: CD34+CD38+CD10CD45RA+CD123+; B-NK: CD34+CD38+CD10+.* ***c.*** *UMAP highlighting classification obtained from a consensus scheme combining the schemes of Doulatov et al., Karamitros et al. and Psaila et al., HSC: CD34+CD38-CD10-CD45RA-CD90+; MPP:CD34+CD38-CD10-CD45RA-CD90-; LMPP:CD34+CD38-CD10-CD45RA+; MLP: CD34+CD38-CD10+; CD71-CD41- MEP: CD34+CD38+CD10-CD45RA-FLT3-ITGA2B-TFRC-; CD71+CD41- MEP: CD34+CD38+CD10-CD45RA-FLT3-ITGA2B-TFRC+; CD71+CD41+ MEP: CD34+CD38+CD10-CD45RA-FLT3ITGA2B+; CMP: CD34+CD38+CD10-CD45RA-FLT3+; GMP: CD34+CD38+CD10-CD45RA+; B-NK: CD34+CD38+CD10+. The marker CD135, CD41, CD71 were not part of the 97 Abseq panel. The expression of the corresponding genes, FLT3, ITGA2B and TFRC, were smoothened using MAGIC (van Dijk et al., 2018).* ***d.*** *UMAP highlighting cell type classification obtained from the decision tree (see also Figure 6).* ***e.*** *Boxplot depicting the median intra-gate dissimilarity for each of the classification schemes shown. Intra-gate dissimilarity is defined as one minus the average Pearson correlation of normalized gene and surface antigen expression values of all cells within each gate.*

3. While the data sets from total bone marrow populations have considerable depth in terms of cell numbers, the CD34+ data set may be too small to detect relevant cell populations – for example LMPPs constitute about 0.1% of CD34+ cells. There seems to be a notable overidentification of these cells in panel g in particular. Generally, signature-based identification will identify the cell most like one sought after, but if the threshold is set too low it will find the closest alternative. These limitations need to be clearly stated, and it would be worth considering the generation of a larger data set.

We thank the reviewer for the comment and apologize for the imprecise use of terminology for immature cell subsets. The classical flow cytometry scheme by Doulatov et al., 2010 identifies a rare population of so called multilymphoid progenitors (MLPs). Using CD10 marker expression, this population was later split into LMPPs (CD10-) and MLPs (CD10+) (Karamitros et al. 2018). By contrast, single cell transcriptomic studies have identified immature cells with lympho-myeloid priming, which are mostly part of the immunophenotypic MPPs (Figure 1b, Figure 6d). We had originally termed those cells LMPPs due to their lympho-myeloid priming. In order to avoid confusion, we now strictly use the term LMPP and MLP for populations defined by surface markers. The term 'lympho-myeloid progenitors' is now used for populations defined by Abseq or Smart-seq2 populations.

Importantly, our single-cell proteo-genomic data enables us to annotate populations both by immunophenotype and by unsupervised clustering. Indeed, immunophenotypic LMPPs and

MLPs, which constitute extremely rare subpopulations, have been covered only by 53 cells (0.3% of CD34+) and 81 cells (0.5% of CD34+), respectively in our original data set. Nonetheless, these cells mapped precisely to the anticipated branching point between lymphoid and monocyte/neutrophil/DC lineages, demonstrating that even extreme rare populations are identified and mapped correctly in our resource (Response Figure 3a, b). In order to increase the number of these rare progenitors, we performed an additional Abseq experiment of the CD34+ compartment of a healthy young BM donor, with additional enrichment of CD38- cells. This enabled us to capture 113 and 150 additional immunophenotypic LMPPs and MLP, respectively, which again mapped precisely to the anticipated locations in the UMAP space (Response Figure 3c, d, now included in the manuscript as new Figure S9 c, d).

We would also like to thank the reviewer for bringing the 'over-identification' of LMPPs in former Figure 6g) to our attention. Importantly, this panel is not representing our main single-cell proteogenomics dataset, but our Smart-seq2 validation dataset that we generated to confirm our findings. We have refined the clustering and cell type annotation. Also, in this dataset we have now refrained from using the term LMPPs to avoid confusion with immunophenotypic gates. We have now outlined the limitations of signature-based cell type identification in this context (line 353-355).

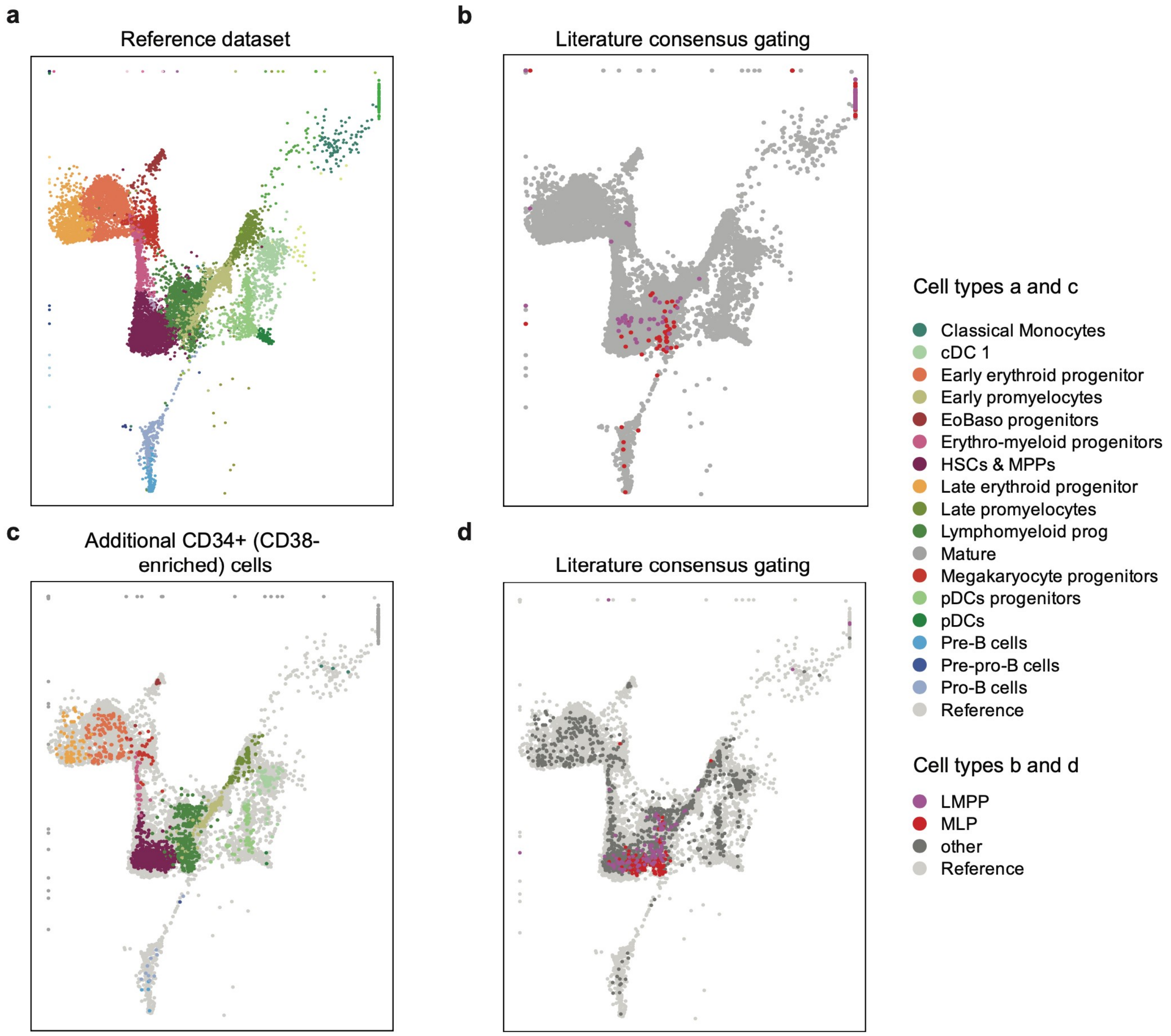

Response Figure 3: Comparison of molecular and immunophenotypic LMPP and MLP populations. *Panels c and d are included in the manuscript as figure S9 c, d. UMAP of all healthy samples displaying **a.** Molecularly defined populations. **b.** Immunophenotypic classification obtained from a consensus scheme recapitulating the scheme of Karamitros et al and Doulatov et al, see Response Figure 2 for details. **c.** UMAP of a new experiment of CD34+ cells with CD38- enrichment, projected on the original coordinate system. **d.** Same as (b) but for the projected cells.*

4. In Figure 3 the pseudotime analysis identified a single myeloid commitment pathway – as the authors themselves point out in the discussion there is now considerable support for two myeloid trajectories, generating distinct subsets of myeloid cell types. It seems possible that only the neutrophil/monocyte trajectory is identified in this analysis. This should be investigated and discussed (and if so, the nomenclature adjusted accordingly).

*We thank the reviewer for raising this point and would like to clarify. We do observe differentiation into all described lineages and sub-lineages of the hematopoietic system (Figure 1b). Indeed, in line with recent studies (Drissen et al., 2019, Science Immunology, Görgens et al., 2013, Cell reports), eosinophil/basophil/mast cell progenitors appear to have common progenitors with erythroid and megakaryocytic lineages, whereas monocytes and neutrophils emerge from the same branch as dendritic cell subsets and lymphoid cell types (Figure 1b). In Figure 3, we had termed the monocyte/neutrophil branch as 'myelocyte' branch as these cells enter a 'myelocyte' cell stage. We do understand that this may cause confusion and have now re-termed this branch into 'monocyte', and explicitly mentioned in the main text that this branch includes neutrophil progenitor stages as well: "*Of note, the monocyte trajectory also includes neutrophil progenitor stages, but mature neutrophils are not included in the datasets due to the use of Ficoll density gradient centrifugation of samples." *(line 203). In total, we have quantitatively investigated five differentiation trajectories: Erythroid, Megakaryocytic, Lymphoid, Dendritic and Neutrophil/Monocyte differentiation (Figure 2, 3 and S6). In principle, it is possible to compute pseudotime trajectories from stem cells into all branches, including pDC and Eosinophil/Basophil differentiation (Response Figure 4). Due to the lower number of cells representing intermediate states observed for the pDC and Eo/Baso lineages, these analyses come with uncertainties, with respect to exact branch-points. We therefore did not include them in the manuscript. We have now more explicitly mentioned this limitation in the results (line 205-207).*

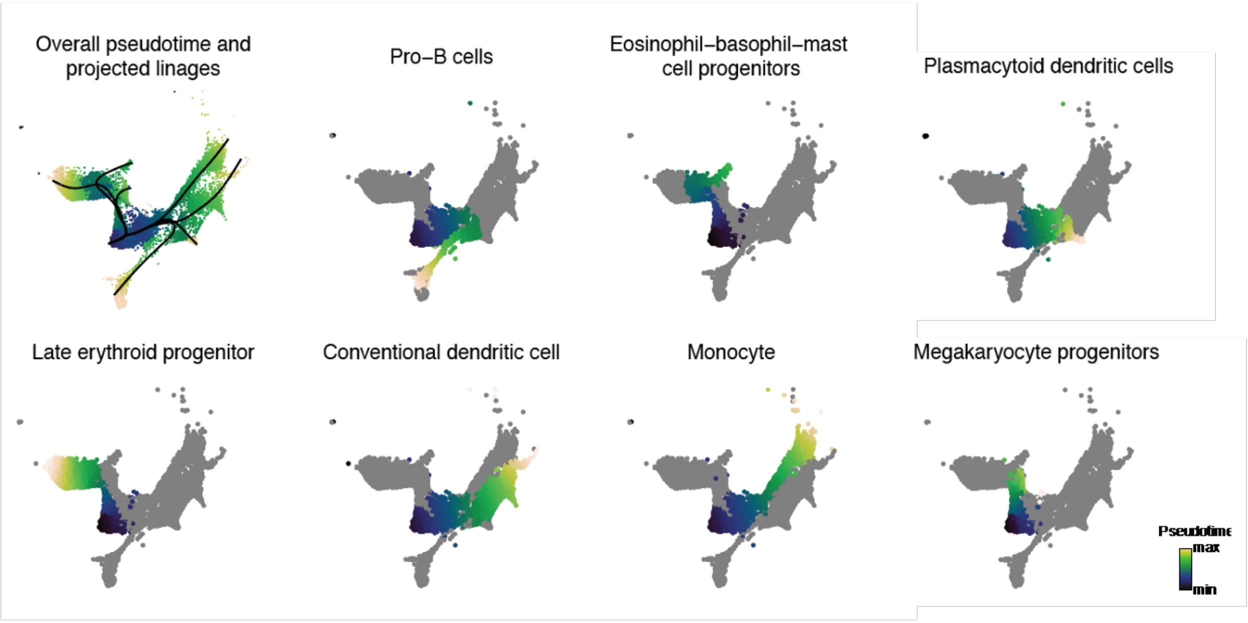

Response Figure 4: Pseudotime trajectories of the CD34+ HSPCs. *UMAPs are depicting CD34+ HSPCs and the overall pseudotime scores (top left) or the score of the respective lineage. In the manuscript Erythroid, Megakaryocytic,*

Lymphoid, Dendritic and Neutrophil/Monocyte differentiation were quantitatively investigated. Trajectories of pDC and Eosinophil/Basophil differentiation were excluded due to low cell numbers of intermediate cell states which results in uncertainties of exact branching points.

5. In the same Figure 3 the evaluation of the identified myeloid and erythroid surface markers is not entirely convincing – the data (panel g) do not show that CD326 is superior to CD71hi as a marker of erythroid commitment (and in fact it seems to miss a number of cells with that fate). Neither CD236 or CD11a are entirely convincing as new useful markers. A better analysis of their potential added value (e.g. in combination with already known markers) could be considered.

We would like to thank the reviewer for this comment. We have now performed additional experiments and analyses to further demonstrate the added value of CD326, CD11a and Tim3. CD326: We have suggested CD326 as a marker for erythroid lineage commitment. Accordingly, we do not expect CD326 expression on all HSPCs with erythroid potential, but specifically on those that show unipotent erythroid cell production. Indeed, index-culture data demonstrate that CD326 expression specifically enriches for colonies that exclusively generate erythroid cells, whereas CD71 enriches for both uni- and oligopotent cells with erythroid potential (new Figure 3g and Response Figure 5a). In line with this, HSPCs with unipotent erythroid potential express significantly higher CD326 levels if compared to all other cells with or without erythroid potential (Response Figure 5b (not included in the main manuscript)). To further evaluate the utility of CD326, we performed flow cytometric analyses in combination with previously used markers (CD71, CD41 and CD44) for the dissection of the megakaryocyte-erythrocyte differentiation path (Psaila et al. 2016). In line with our functional data, flow cytometric and UMAP analyses revealed that CD71^{high} marks erythroid/megakaryocyte lineage restriction, while CD41 and CD326 mark counter-exclusive trajectories corresponding to megakaryocyte and erythrocyte commitment, respectively (Response Figure 5c, partly included in new Figure S6b). Accordingly, CD326 expression appears to be superior to all other marker combinations in identifying committed erythroid lineage-committed progenitors (Response Figure 5d, partly included in new Figure S6b).

CD11a/Tim3: We have identified both CD11a and Tim3 as reliable markers for pan-myeloid hematopoietic differentiation. To further evaluate the utility of those markers, we have compared them to the well-known myeloid differentiation marker CD33. Index-culture data demonstrate that both CD11a and Tim3 expression specifically enriches for colonies that exclusively generate myeloid cells (new Figure 3k, o and Response Figure 5e). In comparison to CD11a and Tim3, CD33 expression is also observed on an increased fraction of HSPCs that have exclusively erythroid potential and a good proportion of cells that show pan myeloid potential don't express CD33. Therefore, gating either on CD11a+ or Tim3+ cells yields higher enrichment of HSPCs with pan myeloid potential than gating on CD33+ alone. In line with this, HSPCs that show

myeloid potential, be it mixed myeloid and erythroid or exclusively myeloid potential, express significantly more CD11a than cells with unipotent erythroid potential, whereas CD33 fails to distinguish HSPCs with unipotent erythroid potential compared to HSPCs with mixed erythroid and myeloid potential (new Figure S6c and Response Figure 5f). In summary, both CD11a and Tim3 represent useful additions for studies of myeloid lineage commitment of HSPCs and can be used as bona fide lineage markers for identification of myeloid or exclusion of erythroid commitment.

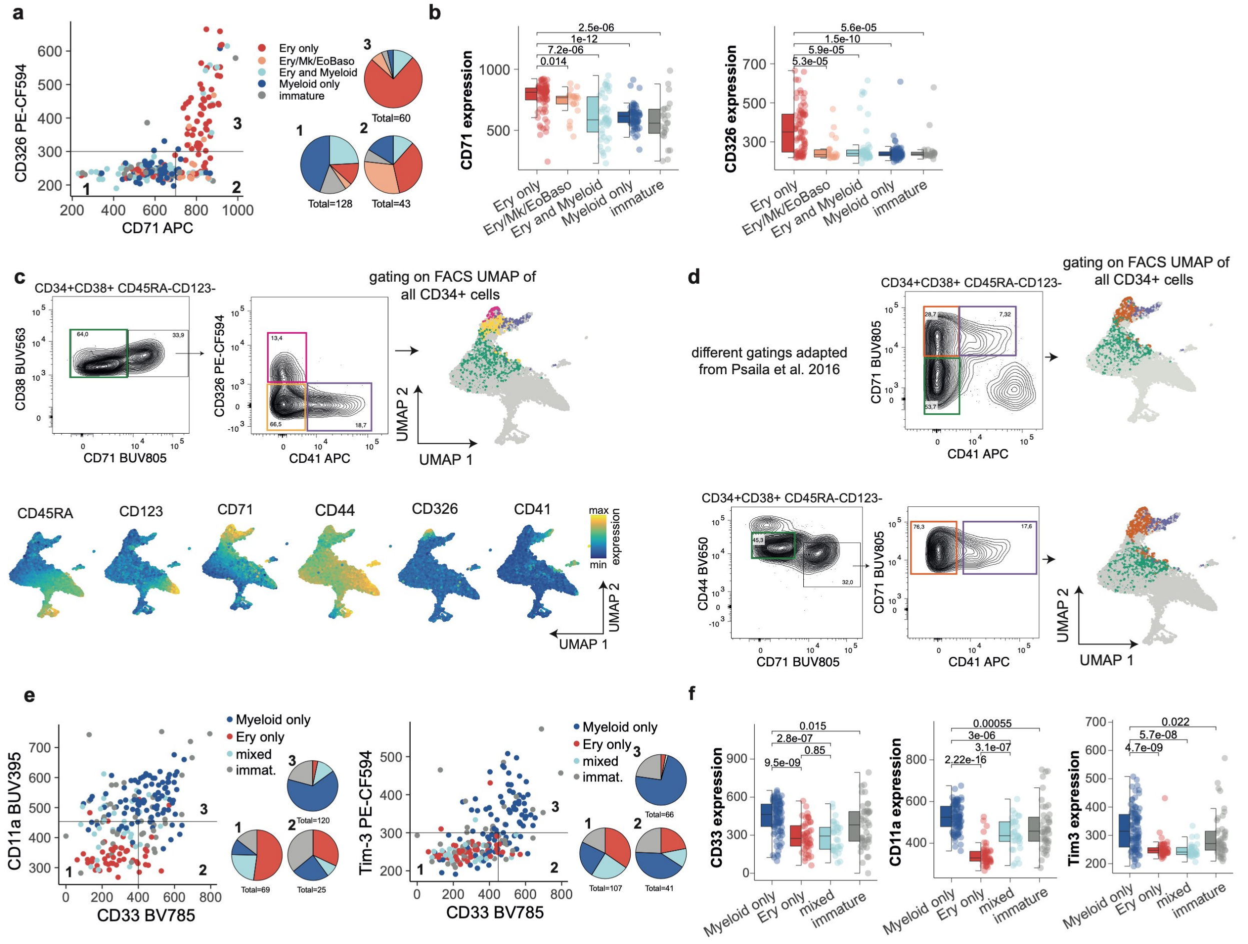

Response Figure 5: Comparison of novel markers CD326, CD11a and Tim3 with known differentiation markers for human hematopoiesis. a. *Single index sorted CD34+ cells are plotted according to their CD71 and CD326 expression and colored by their potential to generate different lineages. The founder cell potential was categorized by their ability to give rise to 1) erythroid only progeny, 2) erythroid, megakaryocytic and eosinophil/basophil progeny, 3) a mix of erythroid and myeloid progeny 4) only myeloid progeny 5) remaining cells with immature phenotypes. Founder cells were subset according to their CD326 and CD71 expression status and the respective culture outcome of individual subsets is shown as pie charts.* ***b.*** *Culture outcome categories described in (a) were analyzed in regard to their CD71 or CD326 surface expression. Wilcoxon rank sum test was used for comparison of individual groups and*

significance levels between groups are depicted. ***c.*** *Gating strategy for subsetting CD71+ erythroid/megakaryocytic HSPCs into CD41+ megakaryocyte progenitors and CD326+ erythroid progenitors. Surface expression values from all CD34+ cells in this experiment were used as input for UMAP dimensionality reduction. Feature plots of CD71, CD326 and CD41 expression highlight the bifurcation within CD71+ HSPCs.* ***d.*** *Gating schemes from Psaila et al., 2016 were applied to the same data as shown in (c) and are unable to demarcate CD326+ erythroid progenitors.* ***e.*** *Similar to (a), single index sorted CD34+ cells are plotted according to their CD33 and CD11a or Tim3 expression and colored by their potential to generate different lineages. The founder cell potential was categorized by their ability to give rise to 1) erythroid only progeny, 2) a mix of erythroid, myeloid, megakaryocytic, eosinophil/basophil progeny 3) only myeloid progeny 4) remaining cells. Founder cells were subset according to their CD33, CD11a or Tim3 expression status and the respective culture outcome of individual subsets is shown as pie charts.* ***f.*** *Culture outcome categories described in (e) were analyzed in regard to their CD33, CD11a or Tim3 surface expression. Wilcoxon rank sum test was used for comparison of individual groups and significance levels between groups are depicted.*

Reviewer #2

Remarks to the Author:

Triana et al report new single-cell proteo-genomic reference maps of the hematopoietic system, and explore how those datasets enable the purification and profiling of more precisely defined cell states**.** The paper comes from one of the internationally leading groupings in single cell genomics analysis of the blood system. The quality of the data looks good, although there are some specific questions (see below). I do not have the required expertise to evaluate the potential novelty of the computational approach of combined interrogation of FACS and CITESEQ data; as a result, comments on this section of the paper will be more limited. There are also not many comments on the analysis of blood cells by progenitor culturing, largely because I think that this aspect of the paper is not developed into any major new biological messages.

Overall, the paper represents an amalgamation of different components, namely an effort to generate a single cell resource, a new computational method for FACS/CITESEQ integration, and some analysis of newly defined blood populations. It remains in my view a judgement call, as to whether this amalgamation has resulted in a “package” of overall high impact.

Specific Comments:

1) I would argue that the authors’ use of the term “surface proteomic map” etc (line 123) is misleading. Proteomic implies the application of proteomics, which is a true “discovery” approach, that explores the entire proteome. The authors however interrogate a pre-fixed set of 97 (or 190 depending on part of paper) set of proteins

We fully agree with the reviewer and now avoid the term 'proteomic' in the context of our data and consistently use the term 'single-cell proteo-genomics', which is now used by community to describe combined transcriptome and surface marker analyses by CITE-seq, AbSeq or similar technologies (see, for example, DOI:10.1158/2643-3230.BCD-21-0046 and https://www.biolegend.com/en-us/totalseq-ebook).

2) Related to point 1, the authors make the point a number of times claiming that this study represents the most extensive analysis of surface proteins by sequencing (the more widely used method for this is the CITESEQ approach, which is similar to the commercial platform used here). The problem with statements claiming 'largest', 'biggest' etc is that they are of course always open to being challenged/outdated. It is clear that at this point, the number of proteins analysed here will not be a stand-out feature of this paper, because there are plenty of papers on biorxiv that have looked at ~200 surface markers (for examplehttps://www.biorxiv.org/content/10.1101/2020.10.12.335331v1.abstract orhttps://www.medrxiv.org/content/10.1101/2021.01.13.21249725v1)

We agree with the reviewer that claims about the 'largest' dataset can be quickly outdated and have removed the respective statement from the manuscript.

3) Following on from point two, the two papers listed there also profiled more total cells. The motivation behind those papers and the paper under review here are different, so the total number of cells profiled is not really the factor by which one should judge the individual papers, but it is still relevant to bear in mind when the authors are trying to sell the paper here as a major reference for the field.

We thank the reviewer for the comment and would like to clarify the key novelties of our data resource and manuscript. Our data reference encompasses a carefully sampled and compiled reference of all major immune cell subsets from bone marrow, including all hematopoietic stem and progenitor cell subsets, of healthy young and aged individuals and leukemia patients. The studies mentioned by this reviewer have profiled peripheral blood mononuclear cells and therefore cannot serve as a reference to the large group of people studying hematopoiesis, B cell differentiation, myeloid leukemias, or other bone marrow specific processes. To further increase the usefulness of our reference to the community, we have now added additional high-parametric single-cell proteo-genomic data from 12 AML patients (see also the reviewer's next point). Beyond our data resource, the key novelty of our manuscript is the comprehensive integration of flow cytometry and single cell RNA-seq, which enables the automated design of gating schemes and interpretation of functional data in the context of single-cell multi-omics landscapes.

4) Coming back to the resource aspect: It is a bit unclear what looking at 3 AML patients adds to this paper, other than concluding that there is large heterogeneity, and one may need to look at dozens of samples to learn anything insightful. Moreover, the fact that the 3 patients generate such different patterns was somewhat surprising, as it seemed that they were chosen to be molecularly similar. There may be very important lessons to be learned from this type of analysis, but like already stated, it probably requires analysis of dozens of patient samples.

To make the AML part of our study more useful for the community, we have now profiled 12 additional AML patients. Together with our original data from 3 initial patients, our study now includes 6 patients with t(15;17) translocated acute promyelocytic leukemia (APL) and 9 normal karyotype AMLs with NPM1 mutations out of which 4 patients carry an additional Flt3 internal tandem duplication (ITD). All 15 AML samples were projected on the healthy reference atlas and the surface marker expression of leukemic cells were contrasted with healthy cells from the corresponding differentiation stage. Inter-patient variability of markers was systematically computed in a cell-state specific fashion. This enabled to (see new Figures 4 and S7):

- *Fine-map the exact developmental stage of leukemia cells across patients (Figure 4d, f)*
- *Perform an unsupervised categorization of phenotypic disease states (Figure 4e, f)*
- *Identify surface markers specific for distinct AML states (Figure 4g)*
- *Identify surface markers that distinguish AML states from their matching healthy cell state (Figure 4i, S7c)*
- *Systematically evaluate the role of patient-to-patient variability in this context. In particular, we show that many previously described, putative leukemia stem cell specific markers display high patient-to-patient variability (Figure 4i)*
- *Demonstrate that the differentiation state of the AML blasts is an important determinant for the presentation of immunoregulatory molecules (Figure 4h)*

Together, this demonstrates that our resource provides a highly useful framework for interpreting hematological malignancies.

5) The genes/primers in Supplementary table 1 should indicate which "category" the gene was classified as.

This information has now been added to the Supplementary Table 1. As described in Supplementary Note 1, categories include genes used to identify mature cell types taken from the human cell atlas (Supplementary Note 1), cell cycle markers (Kowalczyk et al., 2015), genes corresponding to the Abseq antibodies, genes with high variability in single-cell datasets of AML patients (Velten et al., 2021), and stem and progenitor cell markers previously identified (Velten et al., 2017).

6) Can the authors clarify their explanation as to why there is a higher background of specific transcripts in the Abseq stained samples? The authors claim that there is a higher ambient RNA background due to the staining procedure; why would this occur and why is it specific to individual markers (eg HBB, HBD, IL7R and ITGA6)?

Background caused by cell-free 'ambient' RNA is a common phenomenon observed in scRNAseq experiments. The amount of cell-free RNA in a sample is determined by cell type abundance, transcript abundance and cellular stability. Accordingly, highly expressed genes in fragile cell types are typically the main source of ambient RNAs. For instance, hemoglobins (HBB, HBD) represent up to 96% of the RNA present in red blood cells and 80% of RNA in blood extracellular vesicles (DOI:10.21203/rs.2.14503/v1); RBCs are highly fragile (DOI: 10.1038/193884a0). Hence, cellular debris present in sample preps mostly contains extremely high mRNA levels of these transcripts, explaining the abundance of these specific genes in the non-specific background signal. Hemoglobins are consistently among the top hits of ambient RNAs in hematology-related samples (PMID: 33367645)

In our original analysis, we had investigated the correlation in total read counts (i.e. summed reads across all cells) (Response Figure 6a). We noticed that such an analysis is not optimal, since there is always minimal variation in the number of cells sequenced for each cell type, due to the random sampling of a finite set of cells. Hence, we now also investigate the correlation at the level of individual cell types, and found that HBB (and to a lesser extent HBD) are the only genes that differ between the two experiments in these analyses (see Response Figure 6b, c for representative examples). We further noticed a labeling error in the figures: HBB is more abundant in the non-stained experiment. We apologize for this mistake. The difference in observed ambient RNAs between Abseq stained and non-stained samples is likely due to an increased number of washing steps related to the staining procedure, which leads to a dilution of cell-free RNA from the sample. We have updated Supplementary Note 3 (line 87-106) and the associated Figure accordingly.

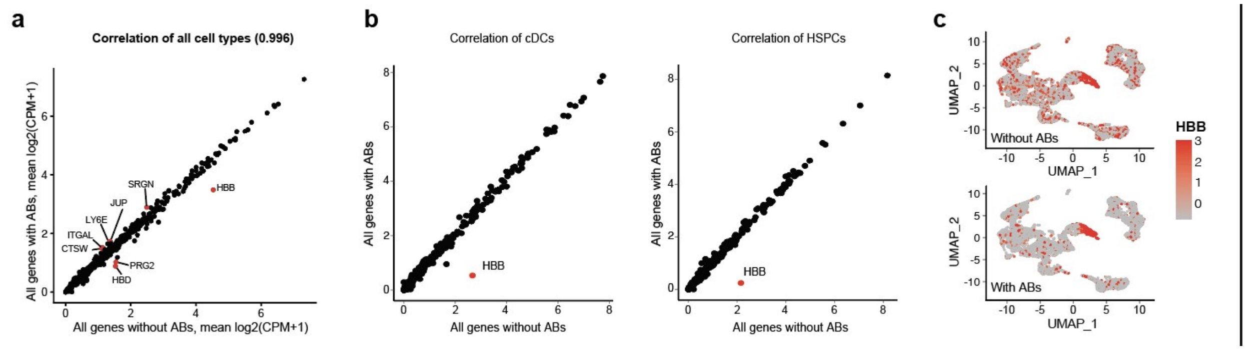

Response Figure 6, also included in Supplementary Note 3: *Comparison of healthy bone marrow samples incubated with and without Abseq antibodies.* ***a.*** *Correlation in the average expression of every gene between samples;*

*top differentially expressed genes are highlighted in red. **b.** Correlation in the average expression of every gene in the cell types cDCs and HSPCs are indicated as representative examples. **c.** UMAP visualization colored by the HBB expression between samples incubated with (lower panel) and without Abseq antibodies (upper panel).*

7) Supplementary note figure 2 shows staining for 97 antibodies not 197 as stated in the text.

Figure S4 (formerly Figure S2) is called in the context of "Besides our main reference dataset, we have generated 'query' single-cell proteo-genomic datasets which are displayed in the context of the main reference. These include, first, the analyses of healthy BM and matched peripheral blood (PB) samples using a 197 plex antibody panel to query the expression of additional surface markers in the context of our reference (Figure S4, Supplementary Table 2)" (lines 167-171). The figure does show a dot-plot of the expression of all 197 antibodies across cell types. Supplementary Note 3 (formerly Note 2) and the associated figure is called in the context of: "First, we performed matched Abseq experiments in the presence or absence of antibodies to ensure that highly multiplex antibody stains do not impact on the transcriptome of single cells (Supplementary Note 3)" (lines 146-148). These analyses use the 97 antibody panel.

8) Supplementary note figure 3 panel a – needs a legend or cell population labels on figure to be able to better compare between this and Figure 1 in main article. Although the cluster numbers should be kept to not confuse the comparison in panel b.

Whole transcriptome analysis (WTA) data has now been projected on the reference coordinate system to enable a comparison. The figure in Supplementary Note 2 (formerly Supplementary Note 3) has been updated accordingly.

9) Supplemental table 4 needs a description/legend.

A legend has been added.

10) It is not clear where the evidence is shown to indicate that there were ~7500 surface molecules per cell detected? The supp figure shows that there are ~7500 antibody reads per cell but not molecules. The authors need to state clearly whether they are using UMIs or not.

We apologize for being imprecise. The protocol is using UMIs (see methods, section: Sevenbridges processing for Abseq processing). We have now updated the figure legend of Figure S3. Panels Figure S3b-c specifies the number of reads. Panels Figure S3d-e specifies the number of UMIs.

11) Spelling mistake on the Abseq-App – following and conventional DCs in pseudotime demonstration (video).

We have fixed the spelling mistake in the Abseq-App.

12) Supple Figure 1 – Can the authors comment on the % of Ab reads; some antibodies appear to be occupying a high percentage of the reads (eg CD18) whereas others appear to be very lowly represented (eg CD20 and CD10). Is this due to the Antibody cocktail or the expression/presence of the markers in the samples? Some large CITESEQ panels are optimized by adding "cold competitor" for some antibodies. Was the same done here?

All antibodies used in this study have been pre-titrated on PBMCs to maximize the signal-to-noise ratio by BD biosciences. Based on further consultation with BD's R&D department, we have diluted antibodies recognizing epitopes with well-known high surface expression (HLA-ABC, CD45, CD11a) as described in the methods section, based on data generated on PBMCs. Accordingly, no 'cold competitor' was added.
The observed variation in reads per antibody is not correlated with the fraction of cells positive for the antibody (e.g. both CD18 and CD20 are expressed in 19% of cells, but CD18 has 63 times more reads), indicating that it is primarily related to the abundance of the antigen on the surface, properties of the antibody, or properties of the panel. If desired, the data displayed in Figure S1 can be useful to decide if and how much 'cold competitor' could be used in future experiments. We have therefore now added the fraction of total reads per antibody also to Supplementary Table S5. A brief discussion of this aspect has been added to Supplementary Note 4 (lines 163-171).

13) Is there an issue of batch between blood and bone marrow in sup fig 2 or it is the way the dots are plotted on the uMAP?

We would like to thank the reviewer for this comment. Indeed, in the display of the data used in the previous submission there was a small shift between bone marrow and blood samples. However, the blood and bone marrow samples have been processed and sequenced back-toback, and there is no indication for a technical batch-effect. The observed shift can be explained by gene expression patterns (particularly of CXCR4) related to homing to the bone marrow (Response Figure 7, top row and new Figure S4b). Based on the comment 25 of this reviewer, we have replaced this display with a projection of the same data on the UMAP coordinates used in Figure 1b (Response Figure 7, bottom row). This has the advantage that users can query the expression of any of the 197 antibodies in the same annotation- and coordinate space as used in the main reference.

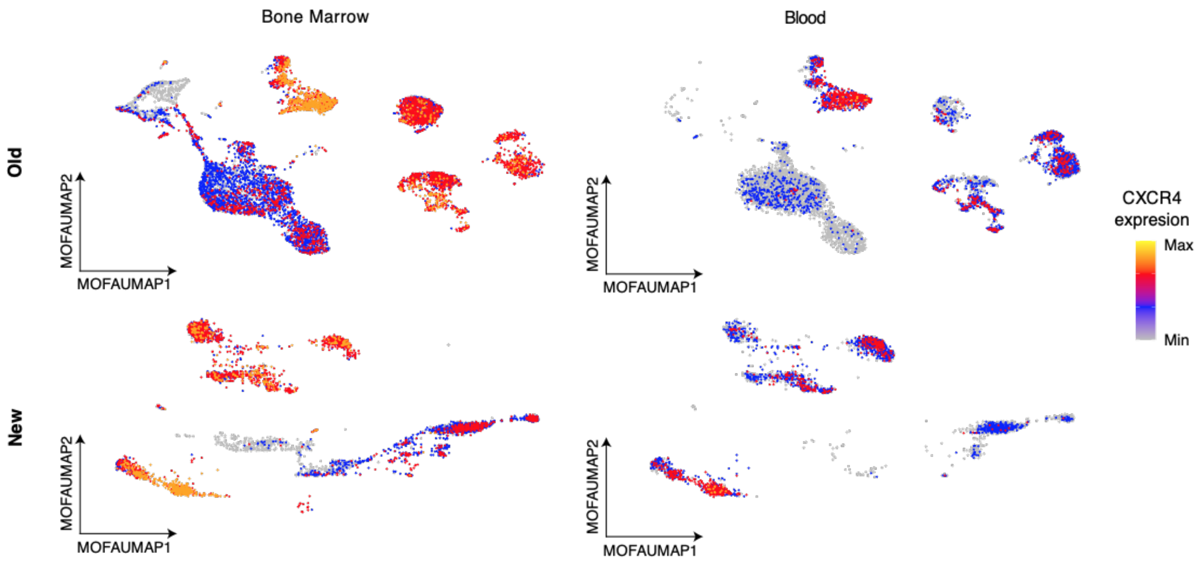

Response Figure 7: *UMAPs highlighting the expression of CXCR4 on blood and bone marrow samples. Top row: Unsupervised integration of bone marrow + blood WTA data. A similar display of the data was used in the original version of Figure S4. Bottom row: Supervised projection of bone marrow + blood WTA data on the main reference data set. This display is used in the revised version of Figure S4.*

14) For the model in Figure 2, are the covariants affected by the expression of the marker and the number of cells on which the marker is expressed; for example CD3 (in which it appears that CD3E, D and G have been pooled together) have a much higher representation within the dataset when compared to CD1a.

We have now systematically investigated if the fraction of variance explained by the covariates is affected by the expression of the marker and the number of cells on which the marker is expressed (Response Figure 8 and included in the Manuscript as Supplementary Figure 5a). We have summarized our results as follows in the methods (Methods line 234-238): "Of note, markers with low absolute expression are more strongly subject to stochastic expression or measurement noise, while markers that are expressed by many different cell types are more strongly subject to technical effects, such as differences in single-cell library quality, likely due to the absence of true biological variability for these markers (Figure S5a). Other covariates are not affected by the expression level of the markers."

And main text (line 191-192): "Non-technical covariates were not affected by marker expression level" (Figure S5a, Methods)

For CD3 we used an antibody clone recognizing CD3 epsilon, which we have clarified in the Supplementary Table S2.

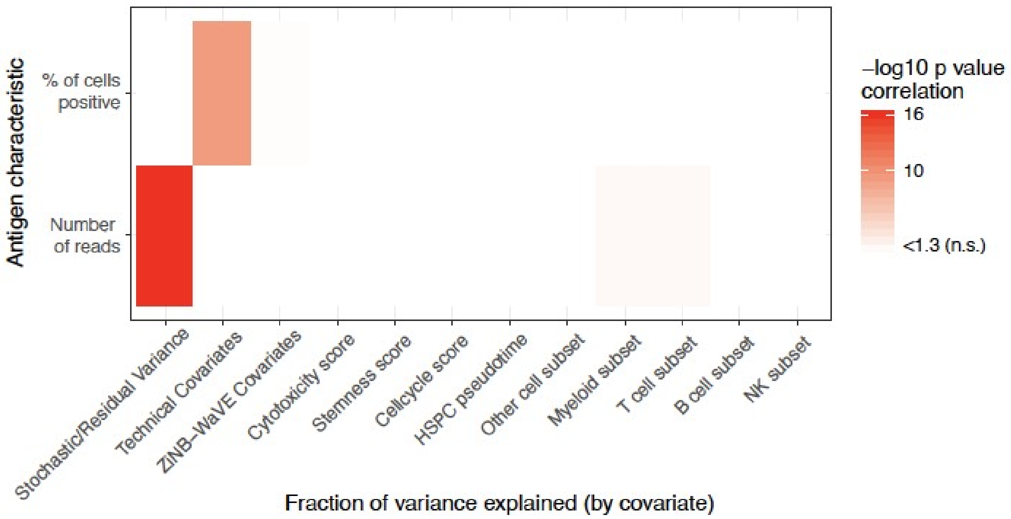

Response Figure 8, included as Supplementary Figure 5a: *Heatmap investigating the correlation between antigen characteristics (y axis) and the fraction of variance in antigen expression explained by different covariates.*

15) The authors mention in the text that they are able to identify novel markers, but then do not give actual examples, just direct the reader to the figure. These should be explicitly stated in the text if the authors are claiming to show previously unknown markers and novelty within their research.

In our manuscript we characterize known markers in-depth, and identify novel markers that have, to our knowledge, not previously been investigated to describe aspects of healthy human hematopoiesis. These include CD326, CD11a, Tim3 and CD98. In the manuscript we clearly state which markers are novel and direct the reader to respective figures (see lines 210-223): "Importantly, our analyses revealed novel surface markers that specifically demarcate distinct stages of lineage commitment, including CD326, CD11a and Tim3 (Figure 2d and 3)…". Figures 3 and S6b, c further characterize these markers. We have now further expanded our functional characterization for CD326, CD11a and Tim3 demonstrating their usefulness as erythroid and pan-myeloid commitment markers (Response Figure 9 and reply to the 5th point of Reviewer #1).

We now refrained from stating novelty or 'novel markers' in cases where no functional validation has been performed.

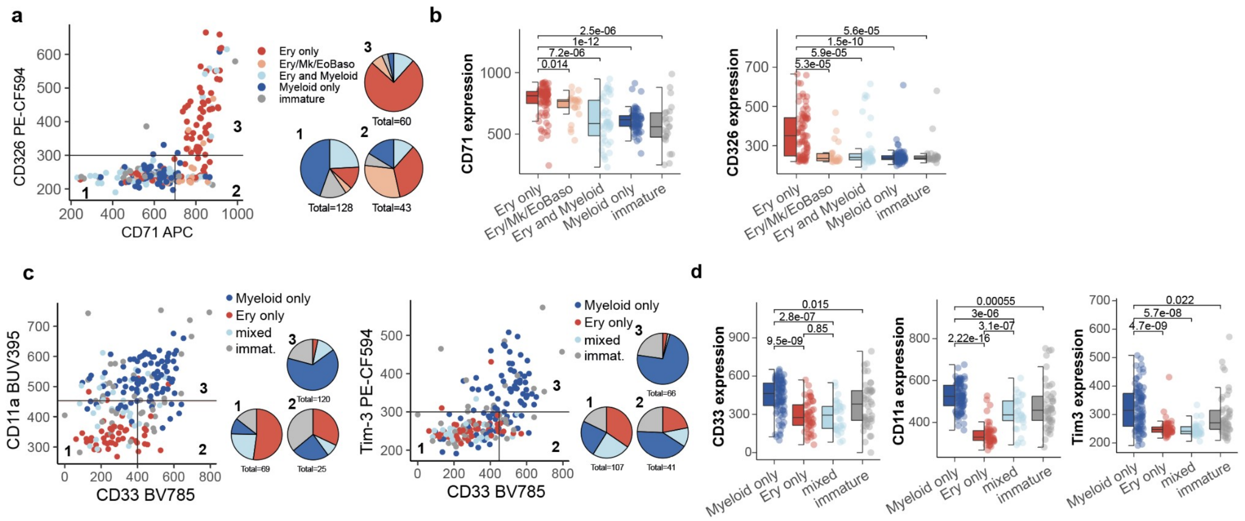

Response Figure 9. Comparison of novel markers CD326, CD11a and Tim3 with known differentiation markers for human hematopoiesis. a. *Single index sorted CD34+ cells are plotted according to their CD71 and CD326 expression and colored by their potential to generate different lineages. The founder cell potential was categorized by their ability to give rise to 1) erythroid only progeny, 2) erythroid, megakaryocytic and eosinophil/basophil progeny, 3) a mix of erythroid and myeloid progeny 4) only myeloid progeny 5) remaining cells. Founder cells were subset according to their CD326 and CD71 expression status and the respective culture outcome of individual subsets is shown as pie charts.* ***b.*** *Culture outcome categories described in* ***a*** *were analyzed in regard to their CD71 or CD326 surface expression. Wilcoxon rank sum test was used for comparison of individual groups and significance levels between groups are depicted.* ***c.*** *Similar to* ***a,*** *single index sorted CD34+ cells are plotted according to their CD33 and CD11a or Tim3 expression and colored by their potential to generate different lineages. The founder cell potential was categorized by their ability to give rise to 1) erythroid only progeny, 2) a mix of erythroid, myeloid, megakaryocytic, eosinophil/basophil progeny 3) only myeloid progeny 4) remaining cells. Founder cells were subset according to their CD33, CD11a or Tim3 expression status and the respective culture outcome of individual subsets is shown as pie charts.* ***d.*** *Culture outcome categories described in* ***c*** *were analyzed in regard to their CD33, CD11a or Tim3 surface expression. Wilcoxon rank sum test was used for comparison of individual groups and significance levels between groups are depicted.*

16) In all the dot plots showing marker expression would it be better to change the scale so that the negative values are blue and 0 is grey, the blue is very drawing of the eye and really what is this showing?

The color scheme in Figure S1 and Figure S4 (formerly Figure S2) has been updated.

17) Figure 3a – spelling mistake

The spelling mistake has been corrected in all the panels

18) The authors should stick to myelocytes or monocytes as the labels change between pseudotime, and uMAP.

We have removed this inconsistency, this differentiation trajectory is now consistently referred to as monocyte.

19) CD98 does not appear to be a specific marker to specifically demarcate distinct stages of HSPC lineage development, it looks very similar to CD38 which the authors have described as a pan-marker. This is clarified in the next statement of the text, but this needs to be clarified, either it is specific to demarcate lineages or it is isotropic.

We thank the reviewer for the comment and would like to clarify. Indeed, CD98 does not specifically demarcate individual lineages, but is 'isotropically' upregulated upon differentiation into all lineages. We therefore now state: "Finally, CD98 was identified as a novel pandifferentiation marker of HSCs, which we confirmed by classical flow cytometry (Figures 2d, and S6d-h)." (lines 219-220).

20) Suppl Figure spelling mistake in y -axis of all panels.

The spelling mistake has been corrected in all panels of figure S6.

21) Figure 4 d needs a legend for the expression of ab and mRNA genes in break-out panel for CD34, CD33, AZUL and DNTT expression.

We have revised the entire Figure 4 in response to the reviewer's 4th point (and a similar point raised by Reviewer #3). As part of these revisions, the cell state heterogeneity of immature-like blasts is now investigated systematically across patients using projection on the reference (see new Figures 4d and e). These plots intuitively make clear that some cells are more LMPP-like, and some cells are more GMP-like. We have therefore removed the former panel d, which used marker genes to make the same point, albeit just in a single patient.
Highlighting of markers originally used in the new coordinate space, demonstrates that they are expressed in the expected cell states, but also reveals extensive patient-to-patient heterogeneity (see Response Figure 10). Both points (cell state differences and patient-to-patient heterogeneity) are investigated much more systematically in the revised manuscript (Figures 4d, e, h and S7c). We therefore do not include this display in the revised manuscript.

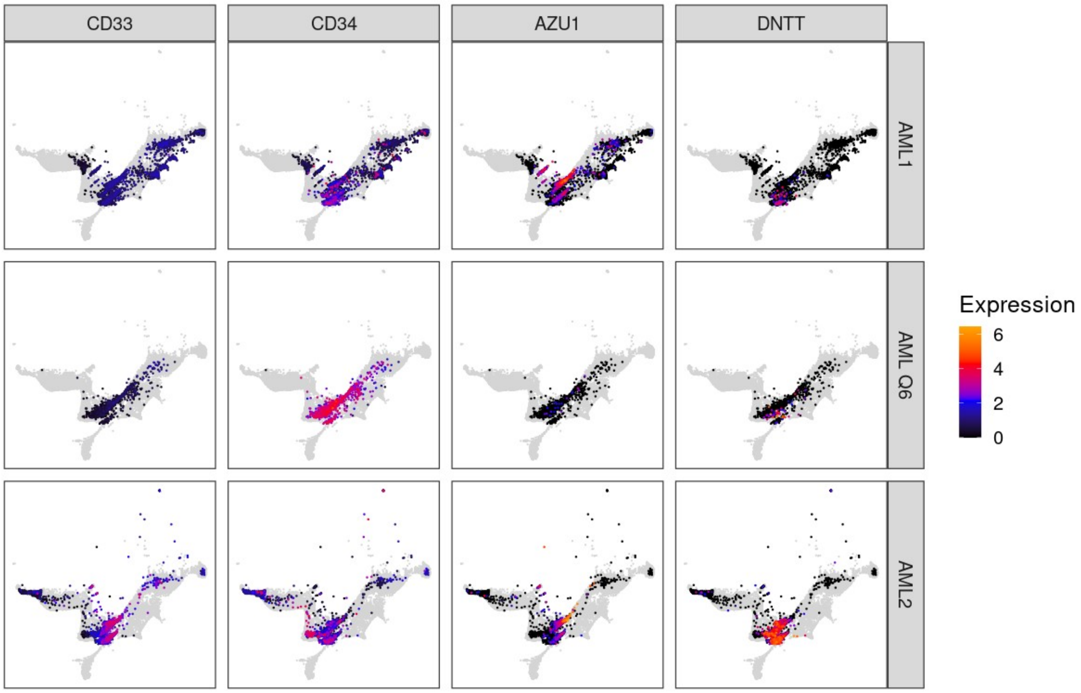

Response Figure 10: *Expression of important markers of myeloid and lymphoid differentiation on cells from leukemia patients with an immature blast phenotype.*

22) Are the changes seen in figure 4f similar across all 3 AML samples?

CD152 and CD274 showed highly similar expression trends across all 15 AML samples; by contrast, Tim3 varied substantially across patients, at least in some cell states (see Response Figure 11a, b, partly included in the new Supplementary Figure 7f).

Furthermore, analysis of our expanded dataset showed that Tim3 was more highly expressed on stem- and MPP-like cells from leukemia compared to healthy stem cells and MPPs (see Response Figure 11c and new Figure 4i), and is therefore not an appropriate example for a marker that closely follows the expression levels observed in healthy myeloid differentiation. We therefore have updated the main Figure 4h (formerly Figure 4f) to only include CD152 and CD274.

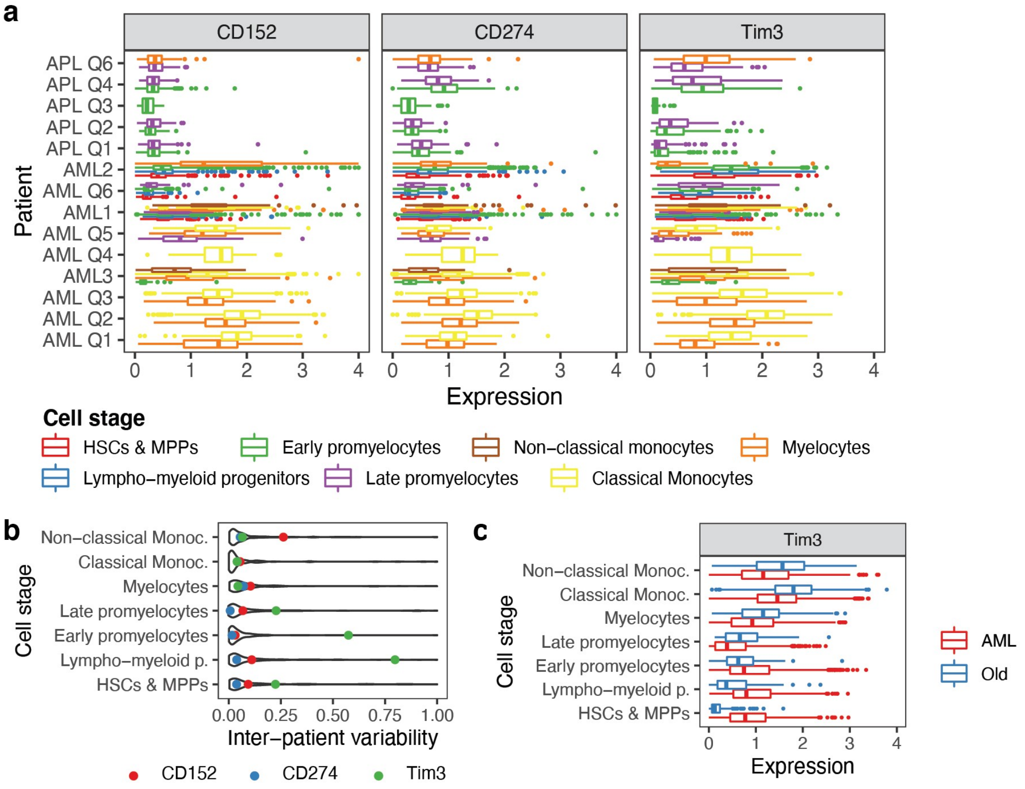

Response Figure 11: ***a.*** *Expression of immunotherapy targets in different cell stages (colors) from different patients (rows). Only cell states represented with at least 50 cells in a given patient are included.* ***b.*** *Violin plot of inter-patient variability scores for all 97 surface markers (background) highlighting the variability of immunotherapy targets (foreground). Tim3 displays highly elevated inter-patient variability in immature cell states.* ***c.*** *Box plot displaying the expression of Tim3 in various populations of old and leukemic individuals.*

23) The AML patients have been compared to young BM samples, should the comparison also not be done for the aged patients?

We thank the reviewer for highlighting the importance of age as a covariate in this comparison. Our expanded AML cohort has an age range of 32 - 78 (female:male ratio 8:7). We therefore decided to account for age and gender as covariates in differential expression testing, and now used the full healthy cohort (young and old, age range: 25-69 years, female:male ratio 2:4) for comparison. Figure 4 and S7 and Supplementary Table S6 have been updated accordingly.

24) PD-L1, CTLA4 and Tim3 (Figure 4f). Where is the evidence that these 3 markers are the only ones showing the block in differentiation?

We thank this reviewer for the comment and would like to clarify this point. We have identified many markers whose expression patterns strongly depend on the stage of the differentiation block of leukemic cells (see Figure S7d for some of the top hits). We have just highlighted the checkpoint inhibition receptors PD-L1 (CD274) and CTLA4 (CD152) due to their potential therapeutic relevance and state the following in the manuscript (lines 266-269): "This also translated into differential surface expression of potential drug targets, such as PD-L1 (CD274) and CTLA4 (CD152) (Figure 4h, S7f), suggesting that the myeloid differentiation program of the AML might be essential in the treatment choice of targeted immune therapies."
Tim3 has been removed from this analysis since analysis of the expanded dataset has shown that it is not an appropriate example of a surface marker retaining a cell state specific expression pattern reminiscent of healthy hematopoiesis.

25) Why is the main focus of the paper on the 97 ab panel? Also, if this is meant to be a reference, it was somewhat unclear how the two large datasets in this paper are integrated to form a single reference?

The core dataset presented here has been generated with 97 antibodies and targeted mRNA profiling across 70,017 cells from 9 healthy and AML individuals. The coverage of cells and donors enables an accurate identification and characterization of all common and rare cell populations. To facilitate an intuitive interpretation of the additional datasets in this study, we now consistently represent them in the coordinate system of this reference (new Figures 4, S4, S9, Supplementary Note 2), except in cases where a fully unsupervised analysis is needed for supporting certain statements. Our projection approach is described in a new Supplementary Note 7 (Supplementary Note lines 430-450). The additional data sets that expand our core reference are:

- *97 antibody & targeted mRNA profiling of CD34+CD38- compartment: Provides higher resolution in the immature HSPC compartment. (Figure S9c, d, newly added during revisions)*
- *97 antibodies & Whole Transcriptome Analysis (WTA): Enables to query any gene's expression in the space defined by our reference and serves to validate that the targeted mRNA sequencing approach does not lead to reduced resolution (discussed in detail in Supplementary Note 2)*
- *197 antibody & targeted mRNA profiling: Enables to query the expression of 100 additional antigens in the context of our reference (Supplementary Figure 4)*
- *12 AML patients profiled for 97 antibodies & targeted mRNA profiling: Illustrates how to interpret new data from diseased individuals in the context of the reference, which we believe will be a common use case for the reference dataset provided here (new Figure 4 and Supplementary Figure 7).*

26) There is no proof that the more defined cellular populations are the “true” functional populations. A more homogenous RNA-Seq signature is not a proxy for this, as this is a circular argument.

We fully agree with this reviewer that a homogenous transcriptomic signature is not necessarily a proxy for a pure functional outcome. One of our main goals was to derive flow cytometry schemes that accurately reflect the complex transcriptomic landscape of hematopoiesis. We have now more clearly stated that the gating scheme was designed to reflect “most adequately the transcriptomic states associated with hematopoietic stem cell differentiation” (lines 335-336), and more homogeneous RNA-seq signatures serve as a technical validation that we have achieved a “faithful identification and prospective isolation of transcritpomically defined progenitor states” (line 358). We have not intended to make any statements on ‘true functional populations’. In contrast, our data suggest that cells with highly similar immunophenotypes and transcriptome can behave functionally differently (Compare new Figure 7g) and see lines 402-409: “Despite strong associations between surface phenotype, transcriptome and function, cells with a highly similar phenotype can give rise to different combinations of lineages (Figure 7g).This observation suggests a role of stochasticity in the process of lineage commitment, or hints towards layers of cell fate regulation not observed in the transcriptome. Taken together, our observations confirm that hematopoietic lineage commitment predominantly occurs continuously along the routes predicted by the transcriptome, with an early primary erythro-myeloid versus lympho-myeloid split (Drissen et al., 2019; Görgens et al., 2014; Notta et al., 2016; Paul et al., 2015; Tusi et al., 2018; Velten et al., 2017) and might help reconciling discrepancies in the interpretation of previous studies.”

Reviewer #3:
Remarks to the Author:

In this manuscript, Triana et al. have put together a comprehensive dataset to compare single cell RNA and protein sequencing to provide insight on cell types and differentiation states. **The authors do a great job of extracting a lot of information and creating a useful database for human hematopoietic cells. The computational programs developed and used to make several conclusions in this manuscript are well developed.**

1) However, there is not a clear scientific advancement in the technique used or the findings. There is a vast compilation of datasets and analysis which may be useful. Comparisons such as young vs. old, or AML are quite powerful when presented with genetic models or with many replicates. – but here, the replicates are few, and the AML samples used are genetically very narrowly defined (and this could affect the dataset enormously). Furthermore, a number of the

proposed novel cell surface markers have been reported previously in other human and murine studies.

We thank this reviewer for the comment. We have now profiled 12 additional AML patient samples to increase replicate numbers and compare distinct genetic backgrounds (see detailed comment below). In this manuscript we have investigated various surface markers in different contexts i) in-depth characterization of known surface markers (Figure 2 and S6A), ii) data-driven selection of surface markers and gating schemes that most accurately describe the hematopoietic differentiation hierarchy (Figure 2, 5 and 6), iii) introduction and characterization of novel surface markers (Figure 3 and S6b-h). While markers in the category 1 have previously been described, markers of category 2 were selected to provide mathematically optimal gating strategies and include both known and novel markers, and markers in the category 3 have, to our knowledge, not been previously investigated in the context of healthy human hematopoiesis (CD98, CD326, Tim3, CD11a). We have now further expanded our functional characterization for CD326, CD11a and Tim-3, demonstrating their usefulness as erythroid and pan-myeloid commitment markers, see the reply to the 5th point of Reviewer #1 and Response Figure 5 for details.

2)There is not sufficient coverage in RNA or Ab reads per cell in some samples to make definitive high confidence statements about cell state, making the quality of the data a concern.

We thank the reviewer and would like to clarify this point. In comparison to whole-transcriptome single-cell RNAseq studies, our total read/UMI counts may appear low. However, with exception of the data discussed in Supplementary Note 2, our data has been generated using a targeted mRNA sequencing approach. For a targeted sequencing approach, our sequencing coverage is extremely high (see for example Schraivogel et al., Nature Methods 2020 for a detailed analysis of sequencing requirements in targeted single cell RNA-seq assays). To actually demonstrate that we have sufficient sequencing depth, we performed downsampling analysis (see Response Figure 12). Cells from all nine samples with an average UMI count of 10,126 per cell were downsampled to an average of 300 UMIs per cell (60 UMIs antibodies, 240 UMIs RNA). We thereby show that even a much lower number of reads is sufficient to distinguish cell types and their differentiation state. We have added the results from Response Figure 12 to Supplementary Note 4, where we also present more quantitative analyses (Supplementary Note lines 142-161). We have further updated Supplementary Figure 3 to use a log scale, which makes it easier to read the sequencing depth used for all samples.

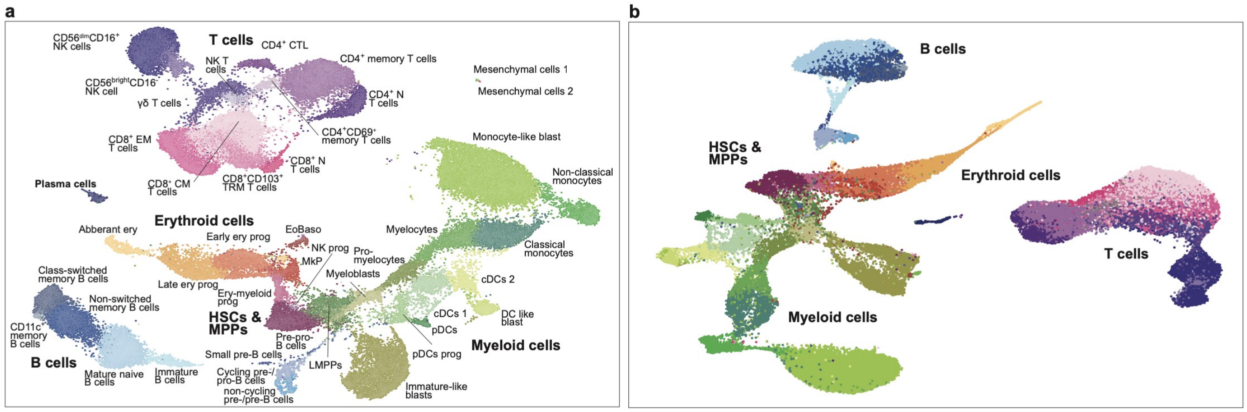

***Response Figure 12, included in Supplementary Note 4. Down-sampled UMIs maintain cell type and differentiation resolution**. **a.** UMAP display of single-cell multimodal RNA and surface protein data of nine human bone marrow with an average of 10126 UMIs per cell. **b.** Cells of all nine samples were down-sampled to 300 UMIs (60 UMIs antibodies, 240 UMIs RNA) and displayed in the UMAP using the clustering obtained from (a).*

3) Finally, the study was performed with frozen cells. Several studies have outlined changes to RNA and protein expression that can stochastically occur after thawing cells.

We thank this reviewer for raising this important point. We have now systematically evaluated the impact of freezing and thawing on our single-cell RNA and protein expression data (see details below).

Major Issues:

1) Cellular RNA and protein are very sensitive to being frozen. Can authors show evidence on whether freeze-thaw cycles affected their data?

In order to address the point of this reviewer, we have now performed extensive experimental analysis to evaluate the effect of freeze-thaw cycles on gene- and surface antigen expression in single-cell proteo-genomics assays. For this purpose, blood was drawn, subjected to Ficoll density gradient centrifugation and peripheral blood mononuclear cells (PBMCs) were frozen or left on ice for 6 hours; after this time interval, blood was drawn again from the same donor and subjected to Ficoll density gradient centrifugation (Response Figure 13a). All three samples (fresh, freeze-thaw, stored on ice) were then processed together and living cells were FACSsorted and stained with 97 surface antibodies before single cell capture. Importantly, global correlations in gene and surface antigen expression were consistently very high between all conditions (freeze-thaw vs fresh = 0.994, freeze-thaw vs on ice=0.994 and on ice vs fresh=0.997; Response Figure 13d, e). UMAP visualization of the data revealed that cell types were unaffected by freezing except for the monocytes, which showed a minor shift upon freeze-thawing (Response Figure 13b, c). As a result of the freeze-thaw process, monocytes upregulated the T

cell costimulatory gene CD275 (ICOS ligand) and the immediate early gene JUN, while downregulated the homing receptor CD62L (SELL), indicative of a stress response (Response Figure 13d). Global gene expression patterns remained unaffected. All in all, the freeze-thaw process had only a minor impact on our data. We have now integrated this analysis in the Supplementary Note 3 (lines 108-123).

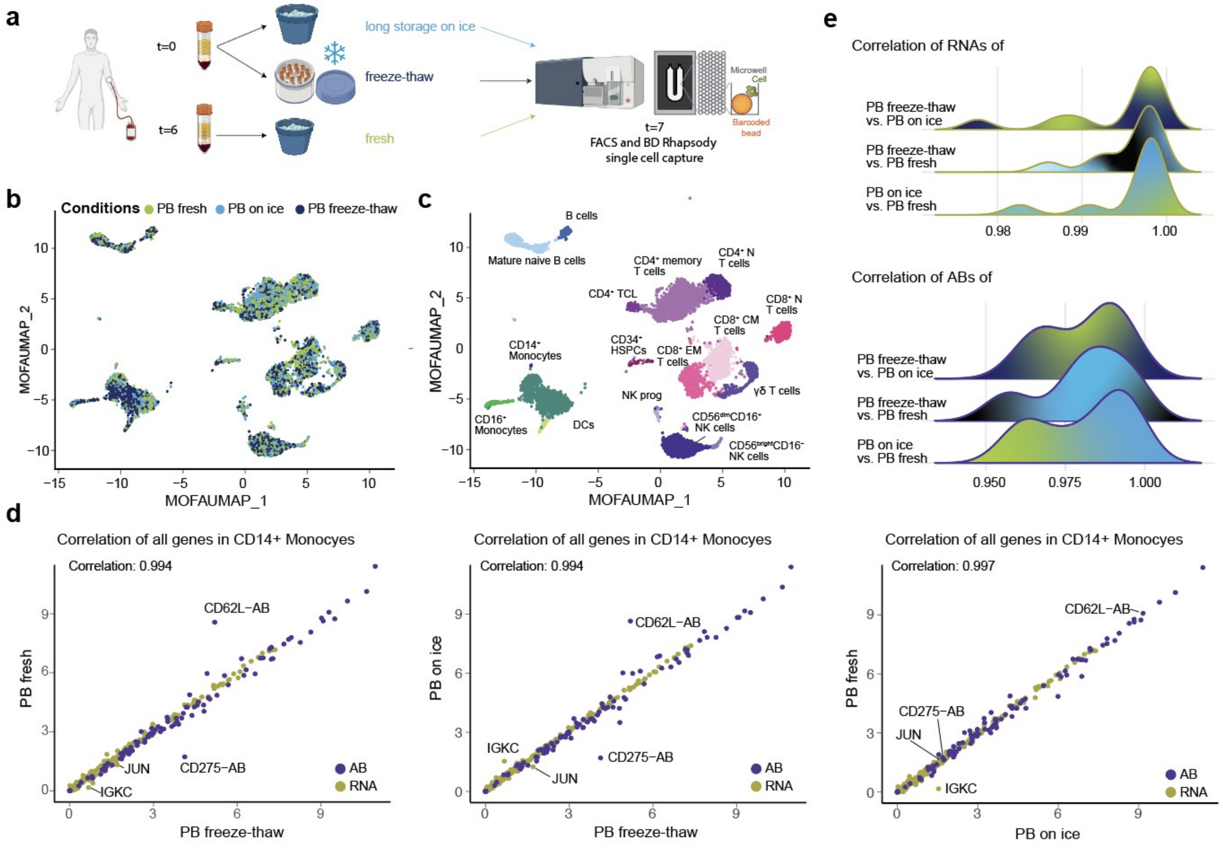

Response Figure 13, included in Supplementary Note 3: Comparison of freshly isolated, stored and frozen PBMC samples. a. *Overview of the experimental setup.* ***b, c.*** *UMAPs were generated on the fresh, on ice and freezethaw PBMC samples sequenced using our targeted scRNAseq and 97 ABs approach. The cells are colored by their condition (a) and cell type (b).* ***d.*** *Correlation between the average expression of each gene per condition. Average expression of JUN, IGKC, CD275-AB and CD62L-AB is highlighted.* ***e.*** *Ridge plots display the coefficient of correlations between the average expression of every gene per cell type and condition. Cell types which were represented by less than 50 cells were excluded from the analysis.*

2) Several of the novel markers identified for HSC and MPP have been published in stand-alone human and murine models.

As mentioned above, the markers CD98, CD326, Tim3, CD11a have, to our knowledge, not been described before for discriminating healthy human HSPC cell states. A detailed summary on the novelty of these markers is provided in the response to the first point of this reviewer. New experimental follow-up data demonstrating a comparison of novel markers to known markers is shown in response to the 5th point of reviewer 1.

3) Since the largest changes were observed in AML patient samples, if authors can add more AML samples with different genetic backgrounds, they may be able to make more general statements and comment on potential targets.

To make the AML part of our study more useful for the community, we have now profiled 12 additional AML patients. Together with our original data from 3 initial patients, our study now includes 6 patients with t(15;17) translocated acute promyelocytic leukemia (APL) and 9 normal karyotype AMLs with NPM1 mutations out of which 4 patients carry an additional Flt3 internal tandem duplication (ITD). All 15 AML samples were projected on the healthy reference atlas and the surface marker expression of leukemic cells were contrasted with healthy cells from the corresponding differentiation stage. Inter-patient variability of markers was systematically computed in a cell-state specific fashion. This enabled to (new Figures 4 and S7):

- *Fine-map the exact developmental stage of leukemia cells across patients (Figure 4d, f)*
- *Perform an unsupervised categorization of phenotypic disease states (Figure 4, f)*
- *Identify surface markers specific for distinct AML states (Figure 4g)*
- *Identify surface markers that distinguish AML states from their matching healthy cell state (Figure 4i, S7c)*
- *Systematically evaluate the role of patient-to-patient variability in this context. In particular, we show that many previously described, putative leukemia stem cell specific markers display high patient-to-patient variability (Figure 4i)*
- *Demonstrate that the differentiation state of the AML blasts is an important determinant for the presentation of immunoregulatory molecules (Figure 4h)*

Together, this demonstrates that our resource provides a highly useful framework for interpreting hematological malignancies (Figure 4e, f).

Minor Issues:

1) Is there evidence of clonal hematopoiesis in the marrow of aged individuals? Are there any mutations that can be attributed to some changes in transcriptomic or proteomic profiles?

We cannot derive information on clonal hematopoiesis. Very different experimental designs need to be employed to investigate this phenomenon using single cell genomics (see, for example, PMID: 32788668 and final figure of PMID: 32203468).

2) All AML patients have an NPM1 mutation. Is there anything novel that may be a prognostic factor for AML patients with these mutations? Also, compare FLT3-ITD WT vs 2 MUT.

Despite the increased number of sequencing AML patients, the identification of prognostic factors is beyond the scope of this study; a more controlled clinical study design, as well as a much larger patient cohort, would be required.

Both FLT3 genotypes are represented in the monocytic and the immature phenotypic class (Figure 4e). In line with this observation, a study that profiled a total of 179 AMLs with bulk RNAseq (van Galen et al., Cell 2019) has found that both FTL3_ITD, NPM1_mut AMLs and FLT3_wt, NPM1_mut AMLs are represented in two phenotypic classes (Progenitor-like and Monocyte-like). We did not identify genes that were systematically differentially expressed between these two genetically defined groups of AML after accounting for cell state. AML patients carrying NPM1 mutations (with or without FLT3_ITD) are extremely heterogeneous in terms of further cooccurring mutations, e.g. in genes such as TET2, DNMT3A, NRAS, etc. In contrast, when comparing the more homogenous APL genotype (PML-RARα fusion) to all other AMLs, we identified several differentially expressed surface markers after accounting for cell state (see Response Figure 14). These analyses identified several makers, including CD18 as expressed in AML, but not APL, in line with previous reports (PMID: 23086776).

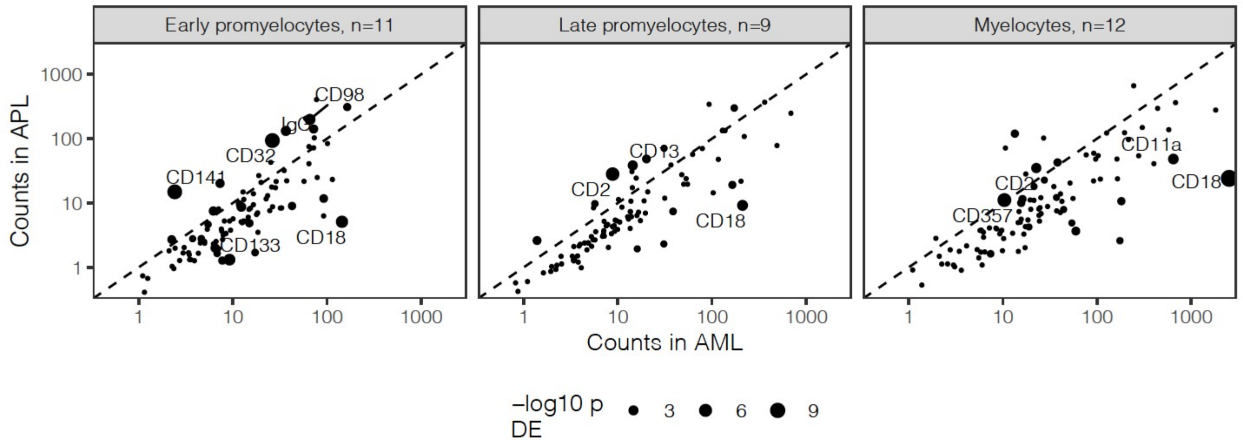

***Response Figure 14:** Expression of surface markers in cell states abundantly present in AML and APL. Point size indicates the p value from a DESeq2-based test for differential expression.*

3) Clarify what types of cells were used in each figure.

*We have now added the information on which types of cells were used to all figure legends, for example in Figure 4, line 655-656: "*For all experiments shown, bone marrow mononuclear cells from iliac crest bone marrow aspirations from healthy adult donors or AML/APL patients were used"

4) Observation that RNA was superior to surface markers in resolving cell states in HSPCs, while surface markers were better for B and myeloid cells is quite interesting. Could the authors comment on why that may be?

We have added the following discussion to Supplementary Note 6, line 410-424. "Our results imply that during stem cell differentiation, mRNA expression is a relatively early step in the process of commitment, compared to surface protein expression. In line with that, we and others have consistently observed lineage priming signatures in cells that surface phenotypically appear immature (see Figure 6d, and see also Velten et al., 2017, Paul et al., 2015). By contrast, in mature cell stages, cellular identity is firmly established and reflected both in the transcriptome and surface protein expression. In mature cell types, antigen expression adds information to mRNA expression alone for three reasons: First, especially in T cells, mRNA measurements are often noisy due to the low RNA content of the cells; Second, in T cells and B cells, the annotation of cell types has historically been performed by immunologists using surface antigens. Relatively similar cell states may have therefore been classified as functionally different based on the expression of a single marker, as in the case of class-switched vs. non-switched memory B cells, that mostly differ in the expression of surface immunoglobulins (IgM, IgD vs. IgG, IgA) while maintaining a very similar transcriptome. Finally, a technical reason for our observations may be that the antibody panel (97-197 antibodies selected based on availability) can be biased towards providing higher resolution in specific cell types, whereas the mRNA panel was designed systematically (see Supplementary Note 1)."

5) Could authors show flow cytometry plots of their CD34+ enrichments?

Please find the flow cytometry plots used for CD34+ enrichment below. We have included this into the manuscript (Figure S2).

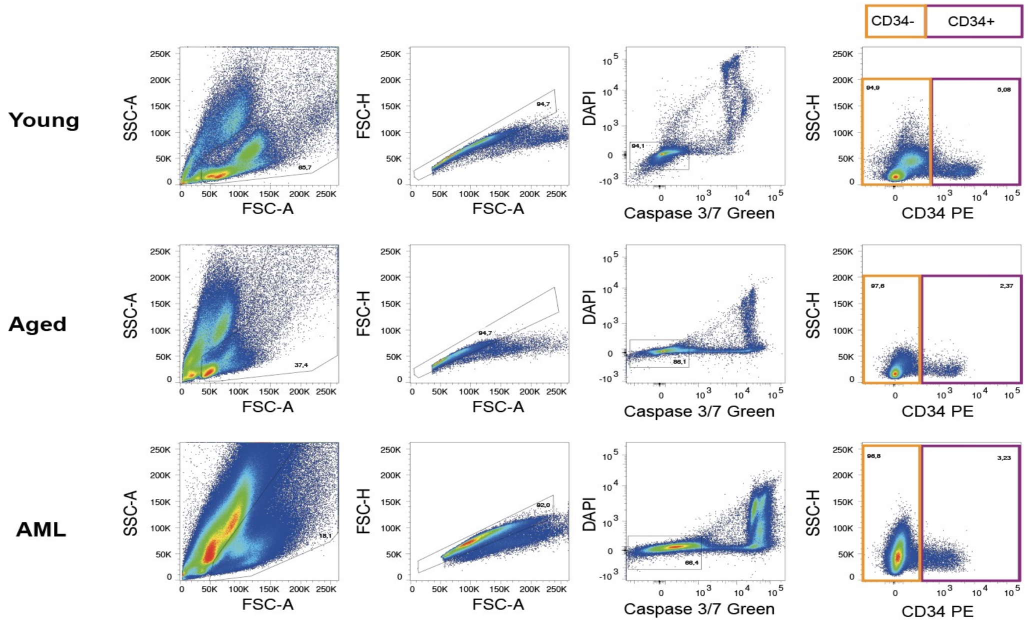

Response Figure 15: *representative gating strategies for different samples used during FACS prior to single-cell capture with indicated CD34+ enrichment gates and CD34- total bone marrow gates.*

Decision Letter, first revision:

Subject: Decision on Nature Immunology submission NI-RS31634A

Message: Dear Dr Haas,

Thank you for your response to the reviewers' comments on your manuscript "Single-cell proteo-genomic reference maps of the hematopoietic system enable the purification and massive profiling of precisely defined cell states". We are happy to inform you that if you revise your manuscript appropriately in response to the referees' comments and our editorial requirements your manuscript should be publishable in Nature Immunology.

It might be helpful for yourselves (and me) to jump onto a quick vid con where we can discuss the revision plan. Please let me know when would work - unfortunately today (23 July) is jammed but most days next week work for me. Please let me know.

Please revise your manuscript according with the reviewers' comments and as outlined in your letter. At resubmission, please include a point-by-point response to the referees' comments, noting the pages and lines where the changes can be found in the revision. Please highlight the changes in the revised manuscript as well.

We are trying to improve the quality and transparency of methods and statistics reporting in our papers (please see our editorial in the May 2013 issue). Please update the Life Sciences Reporting Summary, and supplements if applicable, with any information relevant to any new experiments and upload it (as a Related Manuscript File) along with the files for your revision. If nothing in the checklist has changed, please upload the current version again.

natureresearch

TRANSPARENT PEER REVIEW
Nature Immunology offers a transparent peer review option for new original research manuscripts submitted from 1st December 2019. We encourage increased transparency in peer review by publishing the reviewer comments, author rebuttal letters and editorial decision letters if the authors agree. Such peer review material is made available as a supplementary peer review file. Please state in the cover letter 'I wish to participate in transparent peer review' if you want to opt in, or 'I do not wish to participate in transparent peer review' if you don't. Failure to state your preference will result in delays in accepting your manuscript for publication.

Please note: we allow redactions to authors' rebuttal and reviewer comments in the interest of confidentiality. If you are concerned about the release of confidential data, please let us know specifically what information you would like to have removed. Please note that we cannot incorporate redactions for any other reasons. Reviewer names will be published in the peer review files if the reviewer signed the comments to authors, or if reviewers explicitly agree to release their name. For more information, please refer to our FAQ page.

ORCID

Nature Immunology is committed to improving transparency in authorship. As part of our efforts in this direction, we are now requesting that all authors identified as 'corresponding author' on published papers create and link their Open Researcher and Contributor Identifier (ORCID) with their account on the Manuscript Tracking System (MTS), prior to acceptance. ORCID helps the scientific community achieve unambiguous attribution of all scholarly contributions. For more information please visit www.springernature.com/orcid.

Before resubmitting the final version of the manuscript, if you are listed as a corresponding author on the manuscript, please follow the steps below to link your account on our MTS with your ORCID. If you don't have an ORCID yet, you will be able to create one in minutes. If you are not listed as a corresponding author, please ensure that the corresponding author(s) comply.

1. From the home page of the MTS click on 'Modify my Springer Nature account' under 'General tasks'.
2. In the 'Personal profile' tab, click on 'ORCID Create/link an Open Researcher Contributor ID(ORCID)'. This will re-direct you to the ORCID website.
3a. If you already have an ORCID account, enter your ORCID email and password and click on 'Authorize' to link your ORCID with your account on the MTS.
3b. If you don't yet have an ORCID, you can easily create one by providing the required information and then click on 'Authorize'. This will link your newly created ORCID with your account on the MTS.

IMPORTANT: All authors identified as 'corresponding authors' on the manuscript must follow these instructions. Non-corresponding authors do not have to link their ORCIDs, but please note that it will not be possible to add/modify ORCIDs at proof. Thus, if they wish to have their ORCID added to the paper, they must also follow the above

procedure prior to acceptance.

To support ORCID's aims, we only allow a single ORCID identifier to be attached to one account. If you have any issues attaching an ORCID identifier to your Manuscript Tracking System account, please contact the Platform Support Helpdesk.

We hope that you will support this initiative and supply the required information. Should you have any query or comments, please do not hesitate to contact me.

Nature Immunology has now transitioned to a unified Rights Collection system which will allow our Author Services team to quickly and easily collect the rights and permissions required to publish your work. Once your paper is accepted, you will receive an email in approximately 10 business days providing you with a link to complete the grant of rights. If you choose to publish Open Access, our Author Services team will also be in touch at that time regarding any additional information that may be required to arrange payment for your article.

Please note that you will not receive your proofs until the publishing agreement has been received through our system.

For information regarding our different publishing models please see our Transformative Journals page. If you have any questions about costs, Open Access requirements, or our legal forms, please contact ASJournals@springernature.com..

In recognition of the time and expertise our reviewers provide to Nature Immunology's editorial process, we would like to formally acknowledge their contribution to the external peer review of your manuscript entitled "Single-cell proteo-genomic reference maps of the hematopoietic system enable the purification and massive profiling of precisely defined cell states". For those reviewers who give their assent, we will be publishing their names alongside the published article.

When you are ready to submit your revised manuscript, please use the URL below to submit the revised version: [REDACTED]

We hope to receive your revised manuscript in the next two weeks. Please let us know if circumstances will delay submission beyond this time. If you have any questions please do not hesitate to contact me.

Sincerely,

Zoltan Fehervari, Ph.D.
Senior Editor
Nature Immunology

The Macmillan Building
4 Crinan Street
Tel: 212-726-9207
Fax: 212-696-9752

z.fehervari@nature.com

Reviewer #1 (Remarks to the Author):

The authors thoroughly answered all the comments in the first round of review.

Reviewer #2 (Remarks to the Author):

This manuscript has now been revised based on reviewers' comment. However, while the clarity of the manuscript has been improved, and the technical quality of the analysis is very high, concerns still exist as to whether the results presented provide significant technical or conceptual advance.

1. The authors state that their platform allows the identification of flow cytometric approaches that outperform the state-of-the-art. The case for this is still tentative. For example, the separation of myeloid and erythroid potentials within the CD34+ compartment using the identified markers is not particularly convincing in terms of the purity obtained. Furthermore, it is not obvious how efficiently the assays used read out megakaryocyte or lymphoid lineages, as positive controls for these potentials are not included. It is therefore not clear that this represents an advance compared to existing flow cytometric schemes for the identification of erythroid-restricted (e.g. Psaila et al. Genome Biol. 17:83 (2016)) or myeloid-restricted progenitors (e.g. Manz et al. PNAS 99:11872 (2002)).

2. The description of CD33 as a pan-myeloid marker is true in the sense that CD33 is expressed on myeloid progenitors and mature myeloid cells. However, it is well known that also more primitive HSPCs (including HSCs) express this marker (Taussig, Blood 106:4086 (2005)). CD33 is therefore not necessarily the gold standard for myeloid lineage restriction.

3. The use of gene expression variance as a measure of the accuracy of gating is an interesting concept. However, the genuine issue is whether the functional homogeneity of the cells identified is improved, which is not tested. The proposed conclusion also relies on the ability to accurately assign lineage potentials to cells based on their gene expression, which is difficult, at best. For example, in the present analysis cells that read out as oligo-potent and uni-potent myelo-erythroid progenitors map to the same molecular space (Response Figure 1c), and computational co-localization of lineage potentials will rely heavily on the thresholds used for the level of gene expression required to assign a potential.

4. It is pleasing that the additional analysis performed shows concordance with the more recently proposed branching schemes for myelo-erythroid vs. myelo-lymphoid differentiation, and potentially identifies oligo-potent cells at the appropriate branch points. Still, despite the computational elegance of the analysis, this does not provide any conceptual novelty. Also, performing a probabilistic assessment of the frequency with which lineage potentials are observed in the same cells is higher than expected from culture of total CD34+ cells may not be optimal (Fig 7e). Lineage potentials may be highly correlated in a small subset of progenitors, and the calculated significance of this would then depend on how many more restricted progenitors with the same potentials were included in the analysis. For example, no significant association of neutrophil and

monocyte potentials was observed, and these are co-localised at high frequency in GMPs.

Overall, a technically and computationally accomplished study, but one that may still need additional work to generate results that would have significant impact on the field.

Author Rebuttal, first revision:

Reviewer #2

This manuscript has now been revised based on reviewers' comment. However, while the clarity of the manuscript has been improved, and the technical quality of the analysis is very high, concerns still exist as to whether the results presented provide significant technical or conceptual advance.

1. The authors state that their platform allows the identification of flow cytometric approaches that outperform the state-of-the-art. The case for this is still tentative. For example, the separation of myeloid and erythroid potentials within the CD34+ compartment using the identified markers is not particularly convincing in terms of the purity obtained. Furthermore, it is not obvious how efficiently the assays used read out megakaryocyte or lymphoid lineages, as positive controls for these potentials are not included. It is therefore not clear that this represents an advance compared to existing flow cytometric schemes for the identification of erythroid-restricted (e.g. Psaila et al. Genome Biol. 17:83 (2016)) or myeloid-restricted progenitors (e.g. Manz et al. PNAS 99:11872 (2002)).

- The vast majority of previous publications do not provide any measures of purity in flow cytometry assays. We have systematically compared the purity of our gating strategies to a large number of published strategies for most cell types present in bone marrow, revealing that, without any doubt, the data-driven gating schemes proposed here outperform the vast majority of such historical gating schemes. We assume that the reviewer might has missed these in-depth comparisons, as they are hidden in the supplement (Figure S8). We will include a condensed version of this figure into main Figure 5, as panel a, to appropriately emphasize the extent of comparisons to existing flow cytometry schemes that we have performed, and the strong performance of data defined schemes in these comparisons.
- We have now precisely recapitulated the default gating scheme for the identification of erythroid-restricted and myeloid-restricted progenitors in a 'consensus gating scheme' (including Psaila et al. Genome Biol. 17:83 (2016) and Manz et al. PNAS 99:11872 (2002)), and assayed their functional capacities in a single cell culture assay. Individual populations from the data-defined scheme displayed a functional output comparable to populations of the 'consensus gating' scheme (Response Figure 1a,c), while the datadefined scheme overall provided a higher level of information on functional lineage commitment (Response Figure 1b). We have now included this analysis in to the manuscript (Figure S9e,f and line 357-359).

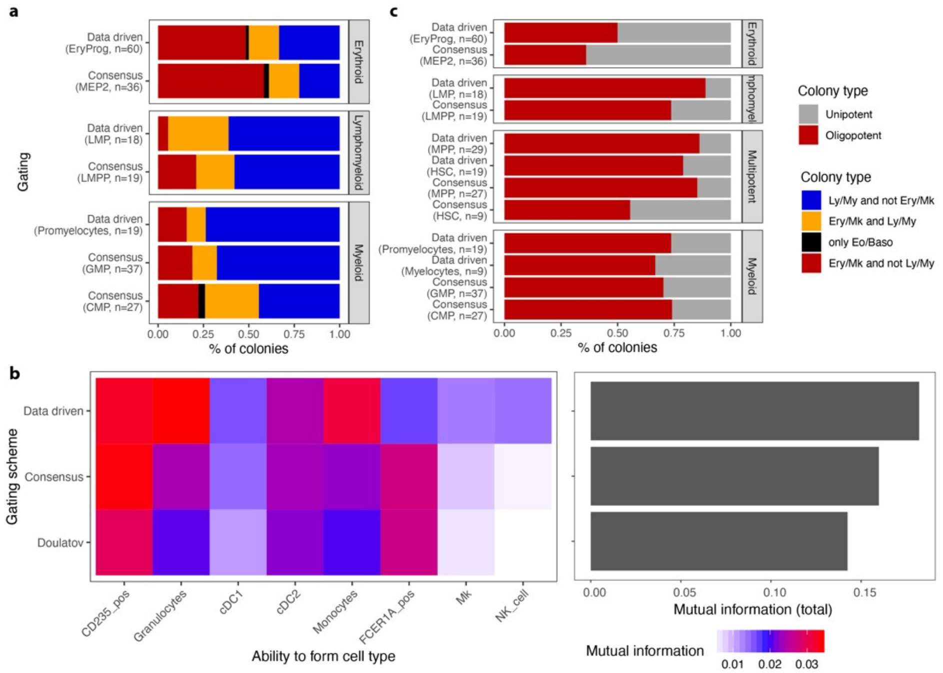

Response figure 1, now included as supplementary figure S9e, f : ***a.*** *Separation of erythroid and myeloid potential by the data driven and the literature consensus gating scheme. Single cells were sorted according to the two gating schemes and cultured for 19 days. Colonies were scored as Ery/Mk if they contained at least 5 erythroid or megakaryocytic cells, and as Ly/My if they contained at least 5 cells of types Neutrophil, cDC, Monocyte, or B/NK.* ***b.*** *Mutual information (in nats) between the gate identity and the ability to form any of the cell types, or the total mutual information across all cell types.* ***c.*** *Separation of unipotent and oligopotent cells by the data driven and the literature consensus scheme. Like a, except that here, cells were scored as unipotent if they gave rise to at least one lineage with >5 cells, or oligopotent if they gave rise to multiple lineages.*

2. The description of CD33 as a pan-myeloid marker is true in the sense that CD33 is expressed on myeloid progenitors and mature myeloid cells. However, it is well known that also more primitive HSPCs (including HSCs) express this marker (Taussig, Blood 106:4086 (2005)). CD33 is therefore not necessarily the gold standard for myeloid lineage restriction.

- We have used CD33 in our manuscript, since it constitutes the most widely used marker for myeloid priming. Our Figure 3 demonstrates that Tim-3 and CD11a are useful alternatives to CD33 as a marker for myeloid priming, as indeed several more primitive, functionally multipotent and even some erythroid-restricted cells express CD33. We have provided further analysis of the expression of all 97 surface markers on the monocyte lineage. This analysis revealed that indeed CD33 is already expressed at baseline levels in the HSC/progenitor compartment, but significantly increases throughout myeloid differentiation (Response Figure 2). Our newly suggested and validated alternatives for myeloid markers, Tim-3 and CD11a, are not expressed in HSCs, but effectively mark myeloid commitment of HSPCs and may therefore act as superior markers. We have now included this analysis in the manuscript as Figure S5g and added a cross-reference in line 203.

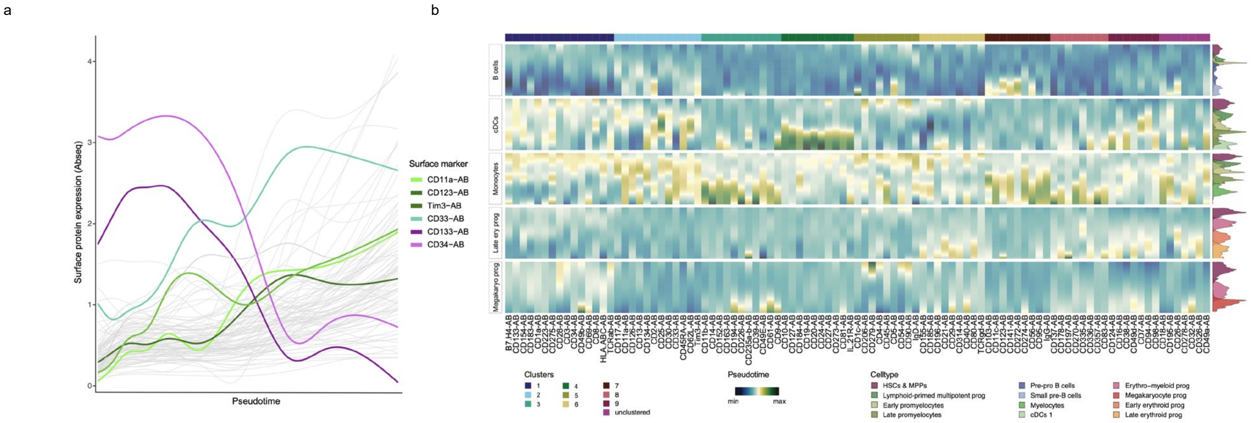

Response Figure 2: Pseudotime of surface proteins. ***a.*** *Line plot depicting the myeloid surface protein marker CD11a, CD33, CD123 and Tim-3 as well as early differentiation marker (CD34 and CD133) smoothened over the monocyte pseudotime trajectory. Gray lines represent the remaining surface protein marker.* ***b.*** *Pseudotime of all 97 surface proteins for the five trajectories (B cells, cDCs, Monocytes, Late erythroid progenitor and Megakaryocyte progenitor). Markers were clustered according to their expression pattern using tradeseq (van den Berge, 2020). The density plots indicate the differentiation stages along the pseudotime. This panel was added to Figure S5 as panel g.*

3. The use of gene expression variance as a measure of the accuracy of gating is an interesting concept. However, the genuine issue is whether the functional homogeneity of the cells identified is improved, which is not tested. The proposed conclusion also relies on the ability to accurately assign lineage potentials to cells based on their gene expression, which is difficult, at best. For example, in the present analysis cells that read out as oligo-potent and uni-potent myelo-erythroid progenitors map to the same molecular space (Response Figure 1c), and computational co-localization of lineage potentials will rely heavily on the thresholds used for the level of gene expression required to assign a potential.

- We have already clarified that our main goal was to derive gating schemes that reflect the **transcriptomic** landscape of the hematopoietic system as accurately as possible. We consider such analysis a major need in the field, since work using transcriptomics to define lineage relationships (e.g. Tusi et al., Nature 2018, Velten et al., Nature Cell Biology 2017, Giladi et al., Nature Cell Biology 2018) reaches partly different conclusions from work making use of functional assays (e.g. Notta et al., Science 2015, Pei et al., Nature 2017, Rodriguez-Fraticelli et al., Nature 2018).
- As correctly observed by the reviewer, functional oligopotency is poorly predicted by gene expression. Of note, the functional homogeneity of populations defined by classical gating schemes is very low, in particular unipotent cells are abundantly included in all so-called multipotent or oligopotent gates, as previously demonstrated (Notta et al., Science 2016, Velten et al., Nature Cell Biology 2017, Karamitros et al., Nature Immunology 2017). We now compared the functional purity of populations of the consensus and data defined scheme (see first point and Response Figure 1a,b). These analyses suggest that the data defined gating scheme and the literature consensus scheme do not display significant differences in their ability to separate functional unipotency and oligopotency (Response Figure 1c). We discuss in the manuscript that

‘This observation suggests a role of stochasticity in the process of lineage commitment, or hints towards layers of cell fate regulation not observed in the transcriptome.’ (lines 406-407).

4. It is pleasing that the additional analysis performed shows concordance with the more recently proposed branching schemes for myelo-erythroid vs. myelo-lymphoid differentiation, and potentially identifies oligo-potent cells at the appropriate branch points. Still, despite the computational elegance of the analysis, this does not provide any conceptual novelty. Also, performing a probabilistic assessment of the frequency with which lineage potentials are observed in the same cells is higher than expected from culture of total CD34+ cells may not be optimal (Fig 7e). Lineage potentials may be highly correlated in a small subset of progenitors, and the calculated significance of this would then depend on how many more restricted progenitors with the same potentials were included in the analysis. For example, no significant association of neutrophil and monocyte potentials was observed, and these are colocalised at high frequency in GMPs.

- We agree that the level of significance depends on the number of lineage restricted progenitors included. Still, this is the correct test statistics to use here, and statistical significance is always related to dataset size. To address the point of reviewer, we can provide an expanded dataset, that we already have hand (n=397 instead of previously n=183 colonies). Analyses on this expanded dataset, reveal that further combinations including Neutrophil/Monocyte and Erythroid/Megakaryocytic also appear significantly enriched as suggested by the reviewer, see figure included below. We have updated Figure 7e, f accordingly. We have made clear that absence of evidence does not constitute evidence for absence, meaning that our data cannot exclude that other oligopotent cell states exist, they just do not present evidence that these are particularly abundant (lines 779-781).

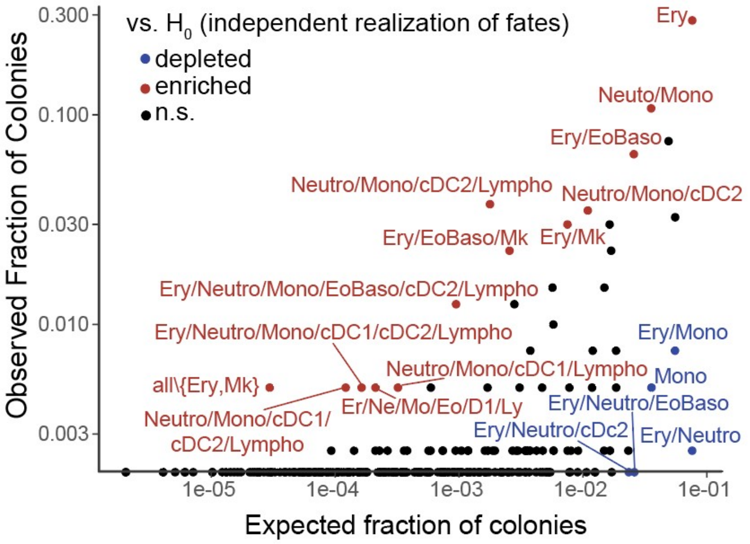

Response Figure 4: Analysis of lineage combinations using an expanded dataset. Replaces Figure 7e.

Overall, a technically and computationally accomplished study, but one that may still need additional work to generate results that would have significant impact on the field.

Decision Letter, second revision:

Subject: Your manuscript, NI-RS31634B

Message: Our ref: NI-RS31634B

1st Sep 2021

Dear Dr. Haas,

Thank you for your patience as we've prepared the guidelines for final submission of your Nature Immunology manuscript, "Single-cell proteo-genomic reference maps of the hematopoietic system enable the purification and massive profiling of precisely defined cell states" (NI-RS31634B). Please carefully follow the step-by-step instructions provided in the attached file, and add a response in each row of the table to indicate the changes that you have made. Please also check and comment on any additional marked-up edits we have proposed within the text. Ensuring that each point is addressed will help to ensure that your revised manuscript can be swiftly handed over to our production team.

We would like to start working on your revised paper, with all of the requested files and forms, as soon as possible (preferably within two weeks). Please get in contact with us if you anticipate delays.

When you upload your final materials, please include a point-by-point response to any remaining reviewer comments and please make sure to upload your checklist.

If you have not done so already, please alert us to any related manuscripts from your group that are under consideration or in press at other journals, or are being written up for submission to other journals (see: https://www.nature.com/nature-research/editorial-policies/plagiarism#policy-on-duplicate-publication for details).

In recognition of the time and expertise our reviewers provide to Nature Immunology's editorial process, we would like to formally acknowledge their contribution to the external peer review of your manuscript entitled "Single-cell proteo-genomic reference maps of the hematopoietic system enable the purification and massive profiling of precisely defined cell states". For those reviewers who give their assent, we will be publishing their names alongside the published article.

Nature Immunology offers a Transparent Peer Review option for new original research manuscripts submitted after December 1st, 2019. As part of this initiative, we encourage our authors to support increased transparency into the peer review process by agreeing to have the reviewer comments, author rebuttal letters, and editorial decision letters published as a Supplementary item. When you submit your final files please clearly state in your cover letter whether or not you would like to participate in this initiative. Please note that failure to state your preference will result in delays in accepting your manuscript for publication.

Cover suggestions

As you prepare your final files we encourage you to consider whether you have any images or illustrations that may be appropriate for use on the cover of Nature Immunology.

Covers should be both aesthetically appealing and scientifically relevant, and should be supplied at the best quality available. Due to the prominence of these images, we do not generally select images featuring faces, children, text, graphs, schematic drawings, or collages on our covers.

We accept TIFF, JPEG, PNG or PSD file formats (a layered PSD file would be ideal), and the image should be at least 300ppi resolution (preferably 600-1200 ppi), in CMYK colour mode.

If your image is selected, we may also use it on the journal website as a banner image, and may need to make artistic alterations to fit our journal style.

Please submit your suggestions, clearly labeled, along with your final files. We'll be in touch if more information is needed.

Nature Immunology has now transitioned to a unified Rights Collection system which will allow our Author Services team to quickly and easily collect the rights and permissions required to publish your work. Approximately 10 days after your paper is formally accepted, you will receive an email in providing you with a link to complete the grant of rights. If your paper is eligible for Open Access, our Author Services team will also be in touch regarding any additional information that may be required to arrange payment for your article.

You will not receive your proofs until the publishing agreement has been received through our system.

Please note that <i>Nature Immunology</i> is a Transformative Journal (TJ). Authors may publish their research with us through the traditional subscription access route or make their paper immediately open access through payment of an article-processing charge (APC). Authors will not be required to make a final decision about access to their article until it has been accepted. Find out more about Transformative Journals.

If you have any questions about costs, Open Access requirements, or our legal forms, please contact ASJournals@springernature.com.

<B>Authors may need to take specific actions to achieve compliance with funder and institutional open access mandates. For submissions from January 2021, if your research is supported by a funder that requires immediate open access (e.g. according to Plan S principles) then you should select the gold OA route, and we will direct you to the compliant route where possible. For authors selecting the subscription publication route our standard licensing terms will need to be accepted, including our <a

href=""https://www.springernature.com/gp/open-research/policies/journal-policies"">self-archiving policies. Those standard licensing terms will supersede any other terms that the author or any third party may assert apply to any version of the manuscript.

Please use the following link for uploading these materials: [REDACTED]

If you have any further questions, please feel free to contact me.

Best regards,

Elle Morris
Senior Editorial Assistant
Nature Immunology
Phone: 212 726 9207
Fax: 212 696 9752
E-mail: immunology@us.nature.com

On behalf of

Zoltan Fehervari, Ph.D.
Senior Editor
Nature Immunology

The Macmillan Building
4 Crinan Street
Tel: 212-726-9207
Fax: 212-696-9752
z.fehervari@nature.com

Final Decision Letter:

Subject: Decision on Nature Immunology submission NI-RS31634C

Message: In reply please quote: NI-RS31634C

Dear Dr. Haas,

I am delighted to accept your manuscript entitled "Single-cell proteo-genomic reference maps of the hematopoietic system enable the purification and massive profiling of precisely defined cell states" for publication in an upcoming issue of Nature Immunology.

The manuscript will now be copy-edited and prepared for the printer. Please check your calendar: if you will be unavailable to check the galley for some portion of the next month, we need the contact information of whom will be making corrections in your stead. When you receive your galleys, please examine them carefully to ensure that we have not inadvertently altered the sense of your text.

Acceptance is conditional on the data in the manuscript not being published elsewhere, or

announced in the print or electronic media, until the embargo/publication date. These restrictions are not intended to deter you from presenting your data at academic meetings and conferences, but any enquiries from the media about papers not yet scheduled for publication should be referred to us.

Please note that <i>Nature Immunology</i> is a Transformative Journal (TJ). Authors may publish their research with us through the traditional subscription access route or make their paper immediately open access through payment of an article-processing charge (APC). Authors will not be required to make a final decision about access to their article until it has been accepted. Find out more about Transformative Journals.

<B>Authors may need to take specific actions to achieve compliance with funder and institutional open access mandates. For submissions from January 2021, if your research is supported by a funder that requires immediate open access (e.g. according to Plan S principles) then you should select the gold OA route, and we will direct you to the compliant route where possible. For authors selecting the subscription publication route our standard licensing terms will need to be accepted, including our self-archiving policies. Those standard licensing terms will supersede any other terms that the author or any third party may assert apply to any version of the manuscript.

In approximately 10 business days you will receive an email with a link to choose the appropriate publishing options for your paper and our Author Services team will be in touch regarding any additional information that may be required.

You will not receive your proofs until the publishing agreement has been received through our system.

If you have any questions about our publishing options, costs, Open Access requirements, or our legal forms, please contact ASJournals@springernature.com

Once your manuscript is typeset and you have completed the appropriate grant of rights, you will receive a link to your electronic proof via email with a request to make any corrections within 48 hours. If, when you receive your proof, you cannot meet this deadline, please inform us at rjsproduction@springernature.com immediately. Once your paper has been scheduled for online publication, the Nature press office will be in touch to confirm the details.

Your paper will be published online soon after we receive your corrections and will appear in print in the next available issue. Content is published online weekly on Mondays and Thursdays, and the embargo is set at 16:00 London time (GMT)/11:00 am US Eastern time (EST) on the day of publication. Now is the time to inform your Public Relations or Press Office about your paper, as they might be interested in promoting its publication. This will allow them time to prepare an accurate and satisfactory press release. Include your manuscript tracking number (NI-RS31634C) and the name of the journal, which they will need when they contact our office.

About one week before your paper is published online, we shall be distributing a press release to news organizations worldwide, which may very well include details of your work. We are happy for your institution or funding agency to prepare its own press release, but it must mention the embargo date and Nature Immunology. Our Press Office will contact you closer to the time of publication, but if you or your Press Office have any enquiries in the meantime, please contact press@nature.com.

Also, if you have any spectacular or outstanding figures or graphics associated with your manuscript - though not necessarily included with your submission - we'd be delighted to consider them as candidates for our cover. Simply send an electronic version (accompanied by a hard copy) to us with a possible cover caption enclosed.

To assist our authors in disseminating their research to the broader community, our SharedIt initiative provides you with a unique shareable link that will allow anyone (with or without a subscription) to read the published article. Recipients of the link with a subscription will also be able to download and print the PDF.

As soon as your article is published, you will receive an automated email with your shareable link.

You can now use a single sign-on for all your accounts, view the status of all your manuscript submissions and reviews, access usage statistics for your published articles and download a record of your refereeing activity for the Nature journals.

If you have not already done so, we strongly recommend that you upload the step-by-step protocols used in this manuscript to the Protocol Exchange. Protocol Exchange is an open online resource that allows researchers to share their detailed experimental know-how. All uploaded protocols are made freely available, assigned DOIs for ease of citation and fully searchable through nature.com. Protocols can be linked to any publications in which they are used and will be linked to from your article. You can also establish a dedicated page to collect all your lab Protocols. By uploading your Protocols to Protocol Exchange, you are enabling researchers to more readily reproduce or adapt the methodology you use, as well as increasing the visibility of your protocols and papers. Upload your Protocols at www.nature.com/protocolexchange/. Further information can be found at www.nature.com/protocolexchange/about .

Please note that we encourage the authors to self-archive their manuscript (the accepted version before copy editing) in their institutional repository, and in their funders' archives, six months after publication. Nature Research recognizes the efforts of funding bodies to increase access of the research they fund, and strongly encourages authors to participate in such efforts. For information about our editorial policy, including license agreement and author copyright, please visit www.nature.com/ni/about/ed_policies/index.html

An online order form for reprints of your paper is available at https://www.nature.com/reprints/author-reprints.html. Please let your coauthors and your institutions' public affairs office know that they are also welcome to order reprints by this method.

Sincerely,

Zoltan Fehervari, Ph.D.
Senior Editor
Nature Immunology

The Macmillan Building
4 Crinan Street
Tel: 212-726-9207
Fax: 212-696-9752
z.fehervari@nature.com
